# Plakoglobin does not participate in endothelial barrier stabilization mediated by cAMP

**DOI:** 10.1038/s41598-025-93756-1

**Published:** 2025-03-16

**Authors:** Ibrahim Hamad, Sara Sepic, Sina Moztarzadeh, Alexander García-Ponce, Jens Waschke, Mariya Y. Radeva

**Affiliations:** Chair of Vegetative Anatomy, Institute of Anatomy, Faculty of Medicine, Ludwig-Maximilians-University (LMU), Pettenkoferstraße 11, 80336 Munich, Germany

**Keywords:** Adherens junctions, Cadherins, Tight junctions

## Abstract

Critical for maintenance of endothelial barrier is the remodeling of the actin cytoskeleton and the precise control of junctional integrity. Plakoglobin (PG) is a structural and signaling protein involved in vascular permeability regulation together with key signaling molecules such as cAMP, Rho GTPases and actin-binding proteins. Here, we investigated the role of PG in cAMP-mediated endothelial barrier stabilization by establishing myocardial endothelial cells derived from wild type (WT) and PG knock-out (PG-KO) mice. Under basal conditions, TEER measurements showed increased barrier function of PG-KO, an effect associated with enhanced protein levels and junctional VE-cadherin and β-catenin accumulation. PG-KO cells also displayed more PECAM-1 and VE-PTP-phosphatase and less phosphorylated VE-cadherin, typically linked with modulation of junctional integrity. PG ablation neither changed the composition of VE-cadherin/β-catenin complex nor activities of Rac1 and RhoA but decreased the basal intracellular cAMP concentration. Remarkably, cAMP augmentation led to enhanced Rac1 activity and TEER in both cell lines, but the effect was less prominent in PG-KO. The tighter barrier in WT was paralleled with more VE-cadherin, β-catenin and cortactin, an actin-binding protein, towards junctions. Surprisingly, PG phosphorylation at Ser665 was not required for cAMP-mediated endothelial barrier integrity, which is different to cardiomyocyte and keratinocyte cell adhesion.

**Supplementary Information:**

The online version contains supplementary material available at 10.1038/s41598-025-93756-1.

## Introduction

The endothelium, lining the inner surface of the blood vessels, is a selective semi-permeable barrier controlling the bidirectional exchange of solutes and water between blood vessels and surrounding tissues. Therefore, disturbance of the barrier integrity, mainly in postcapillary venules, is a hallmark of many pathological processes and life-threatening complications such as inflammation, edema or multiple organ failure. Maintenance of barrier integrity is regulated by fine-tuned opening and closing of mainly two types of endothelial cell-cell contacts: tight junctions (TJs), sealing the intercellular cleft between cells, and the mechanical strength-providing adherens junctions (AJs). The composition and organization of those junctions can vary in endothelial cells from different segments of the vascular tree. Thus, in capillaries and postcapillary venules the TJs are less abundant and loosely organized, making AJs the dominant regulator of junctional integrity^1,2^. Both types of intercellular contacts are composed of transmembrane proteins, namely, the TJ protein claudin-5 and the AJ protein VE-cadherin. These junctional markers are associated directly or indirectly with actin filaments^3 ^and thereby allowing actin remodeling and actin/myosin-mediated cellular machinery to take control over junctional regulation and vice versa. The highly dynamic interaction between transmembrane junctional proteins and actin filaments is achieved through several adaptor molecules such as TJ- related zonula occludens (ZO-1, 2, 3) and VE-cadherin associated members of the catenin family (120-, β- and γ-catenins (also known as plakoglobin (PG))^4,5^.

PG is a structural and functional homologue of β-catenin^6–8^. Both proteins contain a central armadillo repeat core, flanked by C- and N-terminal domains. The central core serves as a host/binding station for various molecules as often the protein partners for PG and β-catenin are shared^9–14^. Analogous to β-catenin, PG promotes cell-cell adhesion by its direct association with the highly conserved cytoplasmic domain of the classical cadherins^15^. The established cadherin/catenin complex is linked to the actin filament network. The anchorage is either direct through α-catenin or indirect via association of α-catenin with actin-binding proteins such as a-actinin, vinculin, ZO-1, spectrin etc., thereby controlling cytoskeleton function^16–22^. Additionally, catenins link VE-cadherin to plenty of signaling molecules (VEGFR2, TGF-βRII/I, Src, SHP-2, DEP-1)^4,23,24^. Importantly, the direct interaction between VE-cadherin and the vascular endothelial protein tyrosine phosphatase (VE-PTP) was also explored as the latter maintains VE-cadherin integrity through PG, which in turn regulates VE-PTP expression via a so far unknown mechanism^25–27^.

Despite the high similarities and shared protein partners between PG and β-catenin it is not yet clear whether they can compensate for each other’s expression. Furthermore, it was reported that the degree of cell confluency correlates with the PG mRNA expression levels^27,28^. Time-course studies also manifested that PG associates with endothelial intercellular junctions after β-catenin and that this junctional accumulation correlates with stable endothelial junctions^28,29^. Additionally, in human dermal microvascular endothelial cells (HDMEC-1), the formation of stable junctional interactions was associated with increased PG expression, followed by β-catenin displacement from cell-cell contact and barrier function promotion^30 ^often shown to be correlated with increased β-catenin signaling and enhanced cell proliferation^31–33^. PG also acts as a signaling molecule, which can translocate to the nucleus and modulate cell transcription or stability of proteins^13,34–37 ^in both a β-catenin-dependent and -independent manner^38–41^.

Furthermore, it is well accepted that cyclic adenosine monophosphate (cAMP), via governing the activity of Rho family GTPases, is a master regulator fine tuning the balance between cadherin-mediated adhesion and contraction and thereby controlling endothelial barrier function^42^. However, it is unclear whether in endothelial cells (ECs) PG participates in the modulation of Rac1 and RhoA activities. The only connection reported was a study showing that PG-deficient keratinocytes display Rho GTPases activation^43^. Moreover, recently was discovered the importance of PG phosphorylation at Ser665 for the cAMP-mediated cellular cohesion in cardiomyocytes and keratinocytes^44–46^ whereas the role of PG phosphorylation for cAMP-mediated endothelial barrier regulation is unknown.

In our current report, new microvascular endothelial cell lines were established to better dissect the role of PG and the effect of its phosphorylation at Ser665 on endothelial barrier integrity under resting conditions and after cAMP-elevation without the limitations commonly associated with siRNA-mediated transient transfection. Here, we show that despite regulating the intracellular cAMP concentration or VE-PTP expression, PG and its phosphorylation at Ser665 are not critical for cAMP -mediated endothelial barrier function.

## Materials and methods

### Mouse models and generation of endothelial cell lines

The mice used in this study were purchased from The Jackson Laboratory or generated by Polygene. The animals were hosted in our local facility. Care, handling, and the euthanasia technique used here were in agreement with the guidelines of the European commission and the regulations approved by the Ethics committee of the Regierung von Oberbayern, Germany (ROB − 55.2–2532.Vet02-19–172). The current study follows the ARRIVE guidelines (https://arriveguidelines.org). All methods were performed in accordance with the relevant guidelines and regulations. To establish endothelial specific plakoglobin knock-out mice (PG-KO), heterozygous floxed *JUP* ^tm1.1Glr^/J, (JUP^fl/+^); Strain #017575, possessing loxP sites flanking exon 1 of the *JUP* gene, were crossed with VEC-Cre transgenic positive mice (VEC-Cre^tg/?^), expressing Cre recombinase under the control of a Chd5 promoter (B6:129-Tg (Chd5-cre)1Spe/J; Strain #017968). The resulting heterozygous for *JUP* and VEC-Cre transgene positive offsprings (JUP^fl/+^VEC-Cre^tg/?^) were bred to obtain JUP^fl/fl^VEC-Cre^tg/?^, which will have an endothelial-specific disruption of both *JUP *alleles. Any mouse missing VEC-Cre was considered as a control animal. Additionally, for generation of PG phospho-deficient cells, where serine (Ser) amino acid at position 665 was substituted with alanine (Ala) (PGSer665Ala), a knock-in mouse model for JUP Ser665Ala was exploited. Detailed description of the transgenic mouse model created by Polygene can be found in a recent publication^44^. The genotype of all offsprings was verified by PCR.

Two to six days old JUP^fl/fl^VEC-Cre^tg/?^, PGSer665Ala and respective control animals were sacrificed by decapitation. The hearts were extracted to obtain myocardial endothelial cells (MyEnds). For easier understanding the cell line missing *JUP*, due to the possession of disordered *JUP* allele, will be called PG-KO and the respective control cell line, with intact *JUP *allele, will be listed as PG-WT. The PG phospho-deficient cells for Ser665 will be labeled as PgSer665Ala. All cells were isolated according to a previously published protocol^47^. Briefly, the entire procedure was performed at room temperature, under the laminar flow hood. The tissues were chopped into small fragments and digested with Trypsin-Collagenase A solution (0,05%/0,02%) for at least 2 h at 37 °C. The digestion was interrupted by adding an equal volume of ice-cold buffer with the following composition: 153 mM NaCl, 5.6 mM KCl, 2.3 mM CaCl_2_ × 2H_2_O, 2.6 mM MgCl_2_ × 6H_2_O, 15 mM HEPES, 1% BSA. The cells were pelleted and resuspended with Dulbecco’s Modified Eagle’s medium (DMEM, Gibco-Thermo Fisher, #41966-029), supplemented with 50 U/ml Penicillin G/Streptomycin (Sigma Aldrich Chemie GmbH, Taufkirchen, Germany) and 10% Fetal calf serum (FCS, Biochrom, #S0115/0247X). The cells were plated in gelatine-coated culture dishes and grown at 37 °C, 5% CO_2_, humidified incubator. One day after seeding, cells were transfected with Polyoma virus middle T antigen secreted by GP + E-86 Neo (GPENeo) fibroblast, resulting in preferential growth of endothelial over non-endothelial cells. After almost 6 weeks of culturing, a homogeneous monolayer of ECs was obtained. To verify the phenotype, specific markers such as von Willebrand Factor (vWF), PECAM-1 and VE-cadherin were tested. To confirm the lack or presence of plakoglobin, PCR, Western blot and immunostaining analyses were run in parallel (Supplementary Fig. 1). All experiments were performed in vitro.

### Transendothelial electrical resistance (TEER) measurements

TEER measurements are a quantitative technique to measure the dynamic of cell-cell contact integrity in cultured cells. The non-invasive assessment of the cellular function in real time was performed by using the ECIS Z Theta instrument (Applied Biophysics Inc, Troy, NYC, U.S.A.), as previously described^48^. Thus, gelatine-coated 8W10E gold microarray electrodes (Ibidi, Martinsried, Germany) were seeded with cells. Few hours prior to any run the medium was exchanged and the electrodes were equilibrated at 37 °C in a 5% CO_2_ humidified atmosphere for at least an hour. All measurements were run at Multifrequency (MTF) mode. Here, the ECIS data presented correspond to the frequency at 4000 Hz, determined previously as the best to detect differences in MyEnds.

### Test reagents and antibodies

Treatment with 5µM of an adenylyl cyclase (AC) activator- Forskolin (F), (Sigma Aldrich Chemie GmbH, F6886) and 10µM of a phosphodiesterase 4 (PDE4) inhibitor- Rolipram (R), (Sigma Aldrich Chemie GmbH, R6520) for one hour was applied to increase the intracellular cAMP levels. Since the compounds were dissolved in Dimethyl sulfoxide (DMSO), corresponding dilution of DMSO was used as a control (Vehicle). Prime activation of Epac1-signaling was achieved by application of 200 µM O-Me-cAMP, (Biolog, C041), solved in water.

The primary and the secondary antibodies used in this study as well as the dilution factor for each application are listed in Tables 1 and 2, respectively.

### Western blot

To prepare cell lysates, confluent monolayers of cells were first washed with ice-cold PBS and then lysed with SDS-lysis buffer, containing 25 mM HEPES, 2 mM EDTA, 25 mM NaF and 1% SDS, pH 7.6. To avoid protein degradation and dephosphorylation, the lysis buffer was upgraded with a combination of cOmplete™ protease inhibitor cocktail (Roche Diagnostics, #11697498001) and PhosStop EASYpack (Roche Diagnostics, #4906845001). Subsequently, cell lysates were sonicated, and the remaining cell debris were removed by centrifugation (1 min, 14. 000 rpm, at 4 °C). The cleared whole cell lysates were transferred to fresh, precooled Eppendorf tubes and determination of the protein concentration was accomplished by using Pierce BCA protein assay kit (Thermo Fischer Scientific, #23225). Prior to loading on SDS-polyacrylamide gel, samples were mixed with Laemmli buffer and boiled at 95 °C for 5 min^49^. Equal amount of protein was loaded on the gel. Proteins were separated by electrophoresis and consequently transferred to nitrocellulose membrane (0.45 μm pore size, Thermo Fischer Scientific, #LC2006). Membranes were blocked for one hour at room temperature with 5% bovine serum albumin (BSA) in Tris-Buffered Saline, containing 0,1% Tween (TBS-T). Then, membranes were incubated overnight at 4 °C with specific primary antibodies (Table 1). After three washing steps with TBS-T, a consequent incubation with secondary antibody (Table 2), conjugated with Horseradish Peroxidase (HRP) was done. After three final washes, the membranes were soaked in enhanced chemiluminescence (ECL) Western Blotting detection reagents. Protein of interest was visualized with Amersham Imager 600 (AI600, GE Healthcare, Munich, Germany). Quantification of the pixel’s intensity was achieved by using ImageJ program (NIH, Windows version 64-bit).

### Immunoprecipitation (IP)

Cells were lysed with NP-40 lysis buffer (10 mM HEPES, pH 7.9, 1.5 mM MgCl_2_ × 6H_2_O, 10 mM KCl, 5 mM EDTA, 2 mM EGTA and 1% NP-40) added with cOmplete™ and PhosStop EASYpack. After 30 min incubation on ice, samples were passed 12 times through a 20G needle. Cell debris were separated and removed by centrifugation at 10,000 rpm for 3 min at 4 °C. Protein concentration was estimated, and 1 mg of total protein amount was used for IP. To bypass variations, each tested sample was adjusted to a volume of 1 ml by adding extra lysis buffer. To avoid non-specific binding to A/G agarose beads (Santa Cruz, #SC-2003), samples were incubated and cleared with pre-washed beads for 1,5 h, at 4 °C. Beads were removed by centrifugation and the supernatant was processed further by mixing it either with VE-cadherin specific antibody or with respective IgG control (Cell Signaling Tech, #2729S). Incubation of the lysates with the respective antibody was performed at 4 °C, overnight on an overhead rotator. On the next day, pre-washed agarose beads were added to the samples as the latter were additionally rotated for 2 h at 4 °C. Beads were collected by centrifugation, mixed with Laemmli buffer, and boiled at 95 °C for 10 min. Lysates were cleared by final centrifugation and loaded on a gel. Western blotting was further proceeded.

**Table 1:** List of primary antibodies.

Antibody	Species	Cat Number	Dilution Rate/Purpose	Company
VE-cadherin	Rabbit	ab33168	1:1000-WB*	Abcam
VE-cadherin Tyr658	Rabbit	44-1144G	1:1000- WB* 1:100-IF#	Thermo Fisher Scientific
VE-PTP	Mouse	610180	1:500- WB*	BD Transduction Laboratories
VE-PTP	Mouse	NBP2-67087	1:50-IF#	Novus Biologica
α-Tubulin	Mouse	ab7291	1:1000- WB*	Abcam
ZO-1	Rabbit	617300	1:1000- WB* 1:100- IF#	Thermo Fisher Scientific
β-catenin	Mouse	61054	1:1000- WB* 1:100- IF#	BD Transduction Laboratories
Plakoglobin	Mouse	61005	1:1000- WB* 1:50- IF#	Progen
PG Ser665	Mouse	house made	Pure supernatant	^45^
vWF	Rabbit	A0082	1:1000- WB* 1:100- IF#	Dako
PECAM-1	Mouse	sc-37676	1:500- WB* 1:50- IF#	Santa Cruz
Rac1	Mouse	610651	1:1000- WB*	BD Transduction Laboratories
RhoA	Rabbit	10749-1-AP	1:1000- WB*	Proteintech
Occludin	Rabbit	40–4700	1:1000- WB*	Thermo Fisher Scientific
Cortactin	Mouse	BDL-868102 or 05-180-AF647	1:1000- WB* 1:100-IF#	Biozol or Millipore
Epac1	Rabbit	ab109415	1:1000- WB*	Abcam
VASP/p-Vasp 157	Rabbit	0012 − 02	1:1000- WB*	ImmunoGlobe
Claudin-1	Mouse	37–4900	1:500	Thermo Fisher Scientific

*WB-Western blot; #IF-Immunofluorescence

**Table 2:** List of secondary antibodies.

Antibody	Species	Cat Number	Dilution Rate/Purpose	Company
Cy3-AffiniPure	Goat Anti-Rabbit IgG (H + L)	111–165-003	1:600-IF	Dianova
Cy3-AffiniPure	Goat Anti-Mouse IgG	115–165-164	1:600-IF	Dianova
Peroxidase-AffiniPure	Goat Anti-Rabbit IgG (H + L)	11–035-003	1:10000-WB	Dianova
Peroxidase-AffiniPure	Goat Anti-Mouse IgG + IgM (H + L)	115-035-068	1:10000-WB	Dianova

## Immunofluorescence

To determine the protein localization pattern, immunostaining of the targets of interest was conducted. For this purpose, cells were grown on gelatine coated 12 mm glass coverslips until they reached confluency. Tight monolayers were first fixed with 4% paraformaldehyde, followed by cell permeabilization with 0.1% Triton-X-100/PBS. Unspecific binding of antibody was blocked by incubation with a mix of 1% BSA (VWR, #422351S) and 10% normal goat serum (TermoFisher Scientific, # 31872) for at least 30 min. Subsequently, an hour incubation with specific primary antibody was accomplished (Table 1). After several washing steps, species-specific secondary antibody was applied for an hour (Table 2). To visualized actin, Alexa Fluor^®^ 488 Phalloidin (Molecular Probes\ Life technologies, #A12379) was used. To stain the nucleus, DAPI, a 4’,6-diamino-2-phenylindole (Roche, #10236276001) was applied. After several washing steps, the cover slips were mounted on microscope glass slides with antifading mounting media (Propylgallate Sigma Aldrich, #3130-100G). All procedures were done at room temperature. The slides were imaged with a laser scanning confocal microscope (Leica SP5) equipped with a HCX PL APO Lambda blue 63 × 1.4 oil immersion objective (Leica).

In brief, to determine the intensity and distribution of the signal along the junctions, a perpendicular line across the junctions was drown, using straight-line tool. Five measurements per image were performed. Each single experiment consisted of at least 3 images per conditions. The data,collected from 3 to 4 experiments , are presented as a bell-shaped curve, where the peak symbolizes the highest signal intensity across thecell border spotted between 2 neighbouring cells. To generate the bell-shaped curve, an equal number of raw data points before and after the peak were utilized, as the more far from the peak points represent the intensity in the intracellular area.

### Polymerase chain reaction (PCR) analysis

To evaluate the mRNA abundance, PCR was performed with cDNA generated from the total RNA extracted either from WT or PG-KO cells. RNA extraction was conducted using RNeasy Plus Mini Kit (Qiagen, #74134). cDNA preparation was accomplished with the SuperScript™ II Reverse Transcriptase (Thermo Fisher, #18064014). Both procedures were run according to the manufacturer’s instructions. In addition to the polymerase specific buffer, dNTPs and the GoTag polymerase itself, primers detecting the targets of interest were extra added to the PCR mixture (see Table 3 for a list of primer pairs with their sequence and the size of the PCR product amplified^50,51^). PCR was also performed by using KAPA2G Fast DNA polymerase with respective buffer, containing dNTPs (Sigma Aldrich Chemie, K5621). Beta-2-microtubulin (B2M) was used to ensure equal loading. Overall, same PCR conditions were used (3 min of initial denaturation at 95 °C, 35 cycles of 30 s denaturation at 95 °C, 30 s primer annealing at 55°/60°C followed by 45 s of extension at 72 °C). After electrophoresis of pre-stained with HDGreen Plus DNA stain (INTAS Science imaging) agarose gel, imaging was done with iBright 1500 instrument (Invitrogen). Quantification of the pixel intensity from the bands was performed using ImageJ.

### Rac1 and RhoA GLISA activation assay

To determine the intracellular concentration of active, GTP-bound Rac1 and RhoA proteins, a colorimetric-based GLISAs for Rac1 (Cytoskeleton, #BK128) and RhoA (Cytoskeleton, #BK124) were used. The assays were conducted according to the manufacturer’s instructions. The absorbance measurements were done at 490 nm, using a TECAN, Infinite 200 PRO microplate reader (Tecan Deutschland GmbH).

### Determination of intracellular cAMP levels by ELISA

Cellular cAMP levels were determined following the manufacturer’s instructions of commercially available cAMP enzyme linked immunosorbent assay (Sigma Aldrich Chemie, #CA200-1KT). By using TECAN microplate reader, absorbance measurements at wavelength of 450 nm were performed.

**Table 3:** Primers used for RT-PCR.

Target analyzed	5’-> 3’	Amplicon size (bp)
Plakoglobin	FW: GTTCGGTTACTGAGTTGCTGCCTTGG REV: GGTATTCCAGGTCACCTTGGTTCTG	369
Cortactin	FW: GGAAGACTGAGAAGCATGCCT REV: CTGGGATTCGTGCTTCTCTGTC	247
VE-cadherin	FW: GAGTTCACCTTCTGTGAGGAGATG REV: CTTCTGCACCTGCGTGTACAC	329
β-catenin	FW: GAGGACCTACACTTATGAGAAGC REV: GGCAGTCCATAATGAAGGCG	492
Occludin	FW: CCTCCAATGGCAAAGTGAAT REV: CTCCCCACCTGTCGTGTAGT	248
ZO-1	FW: CCACCTCTGTCCAGCTCTTC REV: CACCGGAGTGATGGTTTTCT	248
VE-PTP	FW: TCATGGTGACCCAGTGTGTT REV: CTGTGGAGTCTTAGGTCATGCACTG	433
B2M	FW: CAAGTATACTCACGCCACCCAC REV: CATCATGATGCTTGATCACATGTCTC	292
Claudin-1	FW: CATCTACGAGGGACTTGTGGATGTC REV: GGTGTTGGGTAAGAGGTTGTTTTC	451
Claudin-5	FW: GATGTCGTGCGTGGTGCAGAGTAC REV: CTTGTCGTAATCGCCATTGGCCGTG	489
PECAM-1^52^	FW: ATGACCCAGCAACATTCACA REV: CACAGAGCACCGAAGTACCA	199
JAM-A^53^	FW: GGATGGAGAAGATACGAGCT REV: CCAAAGAGCTAGAAAGCCCT	170

### Analysis and statistic

To compare the difference between two analyzed groups, unpaired t-test was used. When three or more groups were investigated, two-way ANOVA, followed by Sidak multiple comparison test was applied. Data are presented as a mean ± SEM as p-values equal or less than 0.05 were considered to be statistically significant.

## Results

### Loss of PG is associated with enhanced endothelial barrier integrity

**Fig. 1:**
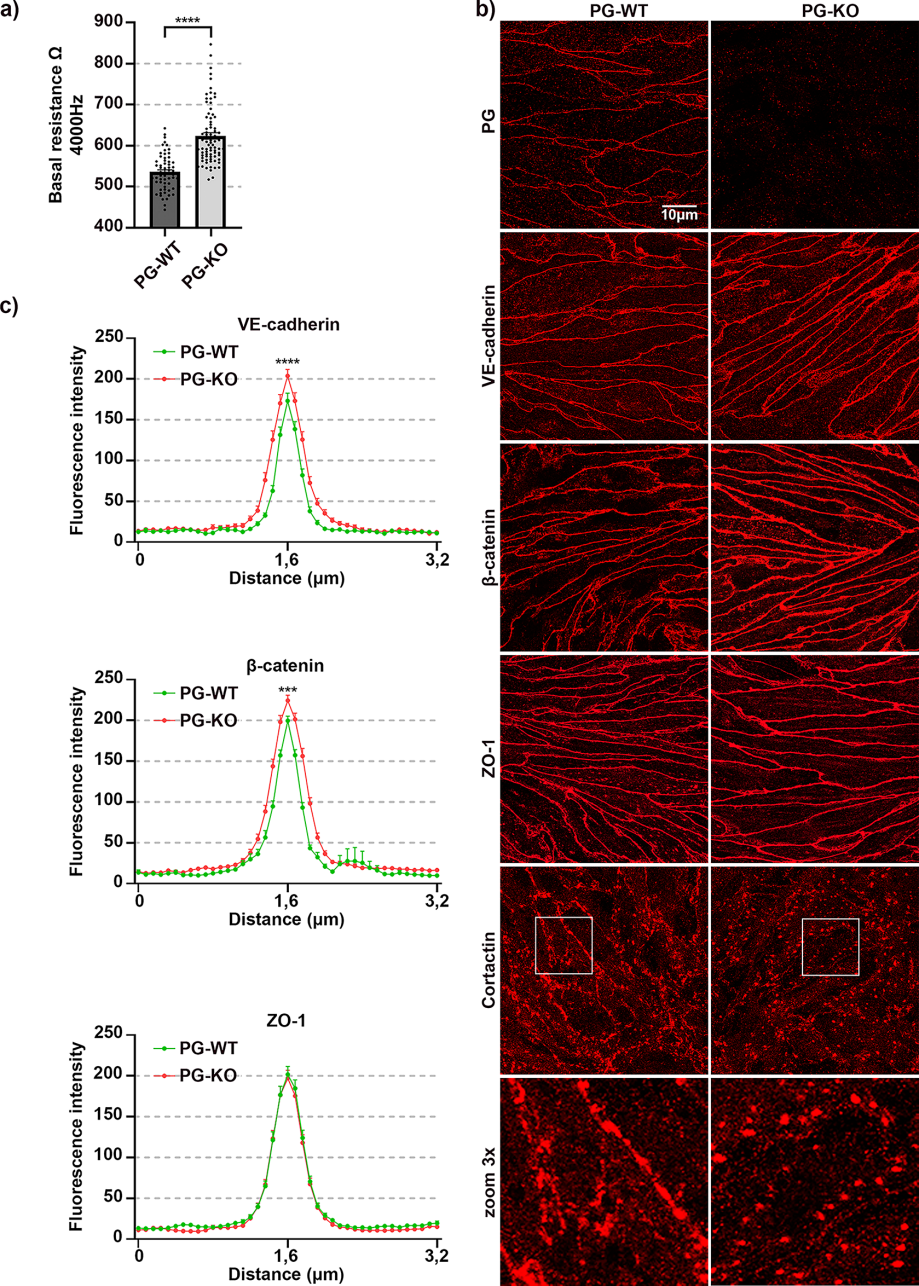
Loss of PG modulates endothelial barrier integrity. (**a**) Bar diagram depicting the average TEER for WT and PG-KO cell monolayers under basal conditions; *N* = 14 (**b**) Immunofluorescence analysis revealing the cellular distribution of VE-cadherin, β-catenin, ZO-1 and cortactin in untreated confluent WT and PG-KO monolayers; *N* = 3 (**c**) Bell-shaped curve disclosing the distribution of signal intensity across the cell-cell contacts for each analyzed protein; *N* = 3. Data are presented as mean ± SEM; the following values were considered statistically significant *** *p* ≤ 0,001, **** *p* ≤ 0,0001.

**Fig. 2:**
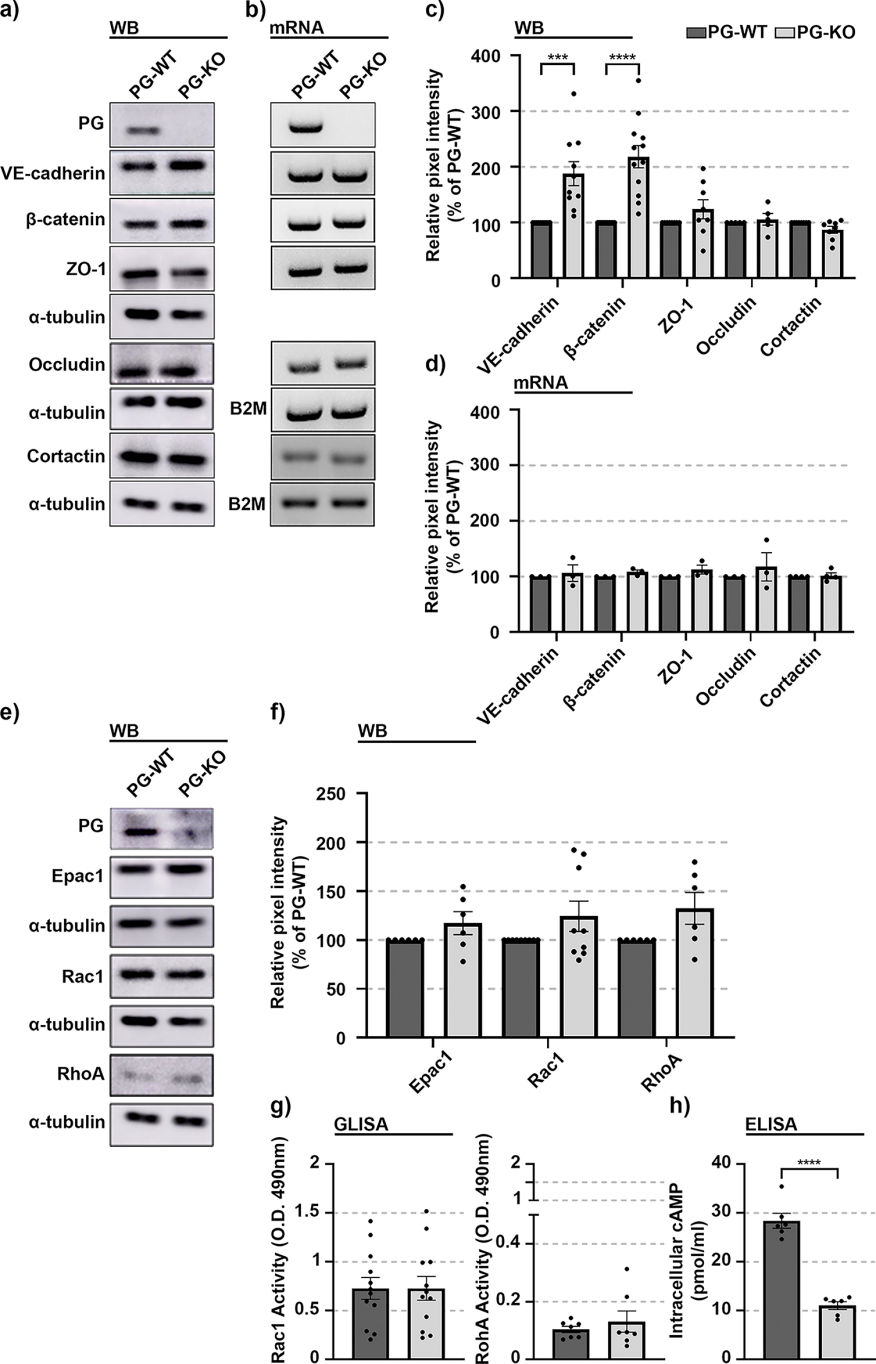
Effect of PG deficiency on central structural and signalling molecules, cAMP levels and small Rho GTPases activity. (**a**) Representative Western blot, *N* ≥ 5 and (**b**) PCR, *N* ≥ 3 analyses of junctional and junctional-related molecules revealing the protein and mRNA expression levels, respectively, in WT and PG-KO cells. α-tubulin was used as loading control for WB. Equal loading for the PCR analyses was validated by B2M expression. (**c**) Bar diagram depicting the relative protein expression of each analysed target of interest in PG-KO when compared to WT cells. (**d**) Bar diagram presenting the mRNA expression in PG-KO, relative to WT cells. (**e**) Western blot analysis illustrating the relative expression of signaling molecules such as Epac1, Rac1 and RhoA upon PG depletion with respective α-tubulin loading control; *N* ≥ 6 (**f**) Bar diagram representing the protein expression of the targets tested in (**e**), relative to WT, after densitometric measurements of the signal intensity were accomplished. (**g**) Bar graph illustrating the activity of Rac1 and RhoA GTPases measured by G-LISA; *N* ≥ 7 h) Levels of intracellular cAMP in untreated WT and PG-KO, detected by ELISA; *N* = 6. Data are presented as mean ± SEM; the following values were considered significant * *p* ≤ 0,05; ** *p* ≤ 0,01; **** *p* ≤ 0,0001.

To assess the effect of complete PG loss on the dynamic of endothelial barrier integrity, we performed TEER measurements. Surprisingly, a significant increase in TEER was discovered for PG-KO when compared to the corresponding WT cells (Fig. 1a). Furthermore, we investigated through the immunofluorescence analysis whether the changes in the barrier function are paralleled with alterations in the cellular distribution of VE-cadherin, β-catenin and ZO-1 as well as the actin-binding protein cortactin. In addition, staining with a PG-specific antibody confirmed the presence or the complete loss of PG in our cells. A prominent accumulation of VE-cadherin and β-catenin towards the junctions was observed in PG-null monolayers and this was confirmed by pixel densitometric measurements across the cell borders (Fig. 1b-c). On the other hand, the localization pattern of ZO-1 was unaffected in PG-depleted cells, and this was verified by signal intensity measurements (Fig. 1b-c). In addition, we tested the effect of PG ablation on the localization pattern of cortactin, since recently we showed that its absence resulted in a critical disruption of VE-cadherin, β-catenin and ZO-1 at cell-cell contacts and demonstrated that cortactin is found within the VE-cadherin-based complex^51^. Thus, despite a diffused intracellular staining for cortactin in both cell lines, we observed more continuous specific signal along the borders in WT cells, while in PG-KO cells the cortactin signal appeared more interrupted, dotted-like (Fig. 1b, white frame for 3x zoom). These results suggest that PG could be required to stabilize cortactin at cellular junctions. Furthermore, we tested whether lack of PG resulted in any critical alterations on mRNA and protein level for several structural molecules. In line with the immunofluorescence analysis, the protein levels of VE-cadherin and β-catenin were significantly higher after PG depletion (Fig. 2a, c), albeit the mRNA levels were unaltered, when compared with WT cells (Fig. 2b, d). Interestingly, in cells lacking PG, regardless of the changes disclosed in the cortactin localization pattern, no deviations were found in the mRNA – and protein expression levels, when compared to WT cells (Fig. 2a-d). Moreover, TJ components including occludin, ZO-1 and claudins as well as members of the immunoglobulin (Ig) gene superfamily, such as junctional adhesion molecules (JAMs) and platelet-endothelial cell adhesion molecule-1 (PECAM-1)^54^ were investigated. Neither the mRNA, nor the protein levels for ZO-1 and occludin were notably changed due to PG knock-out (Fig. 2a-d). Interestingly, the levels of claudin-5 transcript were considerably lower in PG-KO. In contrast, claudin-1 mRNA was significantly elevated, when compared to WT. Although the high expression of claudin-1 mRNA was paralleled with an increased protein level, this effect was not significant (Supplementary Fig. 3a-d). Additionally, neither the mRNA expression of JAM-A nor PECAM-1 was altered due to loss of PG (Supplementary Fig. 3c-d). However, a significant upregulation of PECAM-1 protein was noticed after PG-depletion. (Supplementary Figs. 1 and 3a-d).

### Depletion of PG modulates the intracellular levels of cAMP but does not affect the activity of Rac1 and RhoA

Next, we evaluated the protein expression and activity of central signaling molecules in WT and PG-KO cells. In addition, the intracellular concentration of cAMP as a key second messenger was tested (Fig. 2e-h). Our Western blot analysis revealed that under basal conditions, the protein levels of Epac1, Rac1 or RhoA remained unaltered by PG depletion (Fig. 2e and f). Even though the protein levels of Epac1 were unchanged, this does not directly imply effect on its functionality. Therefore, we examined the barrier resistance after the selective activation of Epac1 via O-Me-cAMP. The TEER measurements demonstrated that O-Me-cAMP application led to slight and steady increase in resistance, and this effect was significant for both cell lines analyzed, demonstrating that the Epac1-signaling pathway is unaffected by the absence of PG (Supplementary Fig. 2a-b). Moreover, by analyzing the phosphorylation of vasodilator-stimulated phosphoprotein (VASP), a major substrate for PKA and crucial regulator of actin dynamics and Rac1 activity in ECs^55^, we speculated that the PKA-mediated signaling was unaltered by loss of PG. Here, no changes in the phosphorylation of VASP were observed under basal conditions, when WT and PG-KO were compared (Supplementary Fig. 2, c). Similarly, no changes in Rac1 and RhoA activity were observed when PG-KO were compared to WT cells, suggesting that in endothelium, Rac1 and RhoA function independently of PG (Fig. 2g). Unexpectedly, the intracellular cAMP levels were significantly dropped in PG-KO indicating that PG may modulate intracellular cAMP concentration (Fig. 2h).

**Fig. 3:**
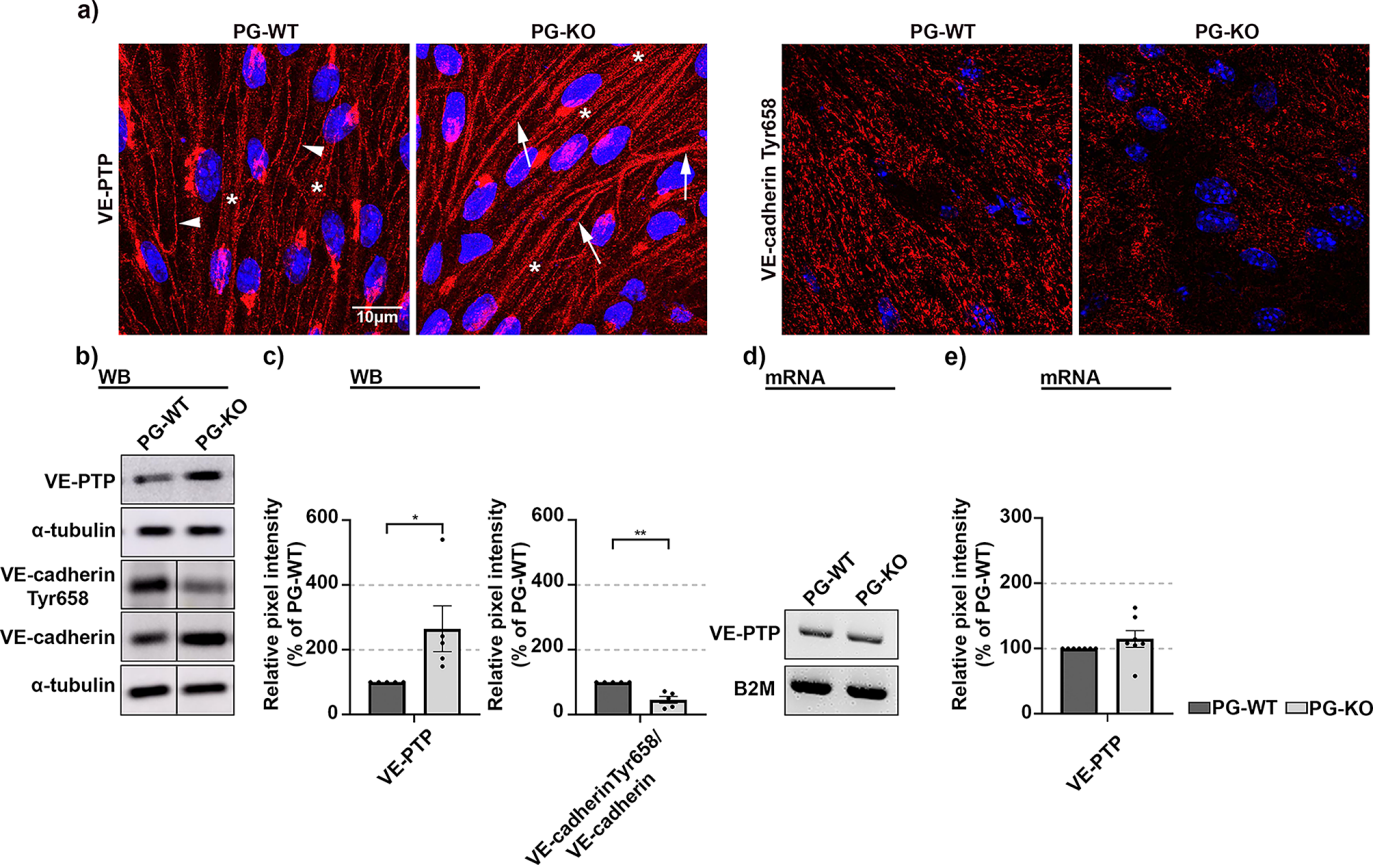
PG controls VE-PTP protein expression and phosphorylation of VE-cadherin at tyrosine 658. (**a**) Representative immunostaining for endothelial specific phosphatase VE-PTP and tyrosine phosphorylated VE-cadherin at position 658 (VE-cadherin, Tyr658), in WT and PG-KO cell monolayers; Stars denote intracellular staining in both cell lines, while arrowheads and arrows represent staining along the junctions in WT and PG-KO, respectively. DAPI was used to label nuclear DNA; *N* ≥ 3 (**b**) Corresponding Western blot analyses, where α-tubulin was used as a loading control. The bands originated from the same membrane and the vertical, continued line indicates excised irrelevant bands. Original blots are presented in Figure S4 p) provided in the Supplementary info file; *N* = 5 (**c**) Bar diagram depicting the protein expression of VE-PTP and VE-cadherin, Tyr658 in PG-KO cells, relative to the expression in WT. For VE-cadherin, Tyr658 the bar diagram represents the ratio between VE-cadherin, Tyr658 and total VE-cadherin. All data were normalized to the WT controls, (**d**) PCR analysis revealing the VE-PTP mRNA expression in WT and PG-KO. Equal loading was validated with B2M; *N* = 7 e) Bar diagram presenting the VE-PTP mRNA expression in PG-KO, relative to WT. Data are presented as mean ± SEM; unpaired t-test; * *p* ≤ 0,05; ** *p* ≤ 0,01.

### PG controls VE-cadherin phosphorylation through modulation of VE-PTP expression

Previous studies recognized the interplay between PG and the endothelial-specific VE-PTP. Thereby, its expression and localization pattern were investigated as a consequence of PG deficit. Immunolabeling with a specific antibody revealed a perinuclear staining and specific VE-PTP-signal along the junctions in both cell lines (Fig. 3a, arrowheads for WT and arrows for PG-KO). However, more pronounced signals towards the cell-cell contacts were detected in PG-KO, where a greater intracellular staining was also noticed in comparison to WT cells (Fig. 3a, starts). Furthermore, Western blot analysis confirmed that PG-depleted cells had significantly higher VE-PTP protein expression (Fig. 3b and c). However, the VE-PTP mRNA levels were unchanged in both cell lines, indicating that in our cells PG modulates VE-PTP protein expression at the posttranscriptional or posttranslational level (Fig. 3d and e). Moreover, we tested the impact of PG-mediated VE-PTP alterations on VE-cadherin tyrosine phosphorylation. Western blot analysis demonstrated that PG-KO exhibited less phosphorylation of VE-cadherin at position Tyr658 in contrast to WT cells (Fig. 3b and c). Similar observations were revealed after immunostaining analysis. Here, the specific staining for VE-cadherin Tyr658 appeared as dotted-like and diffuse in both cell lines, however, in contrast to PG-KO, WT cells disclosed a much stronger and distributed overall signal among the cell monolayers (Fig. 3a).

**Fig. 4:**
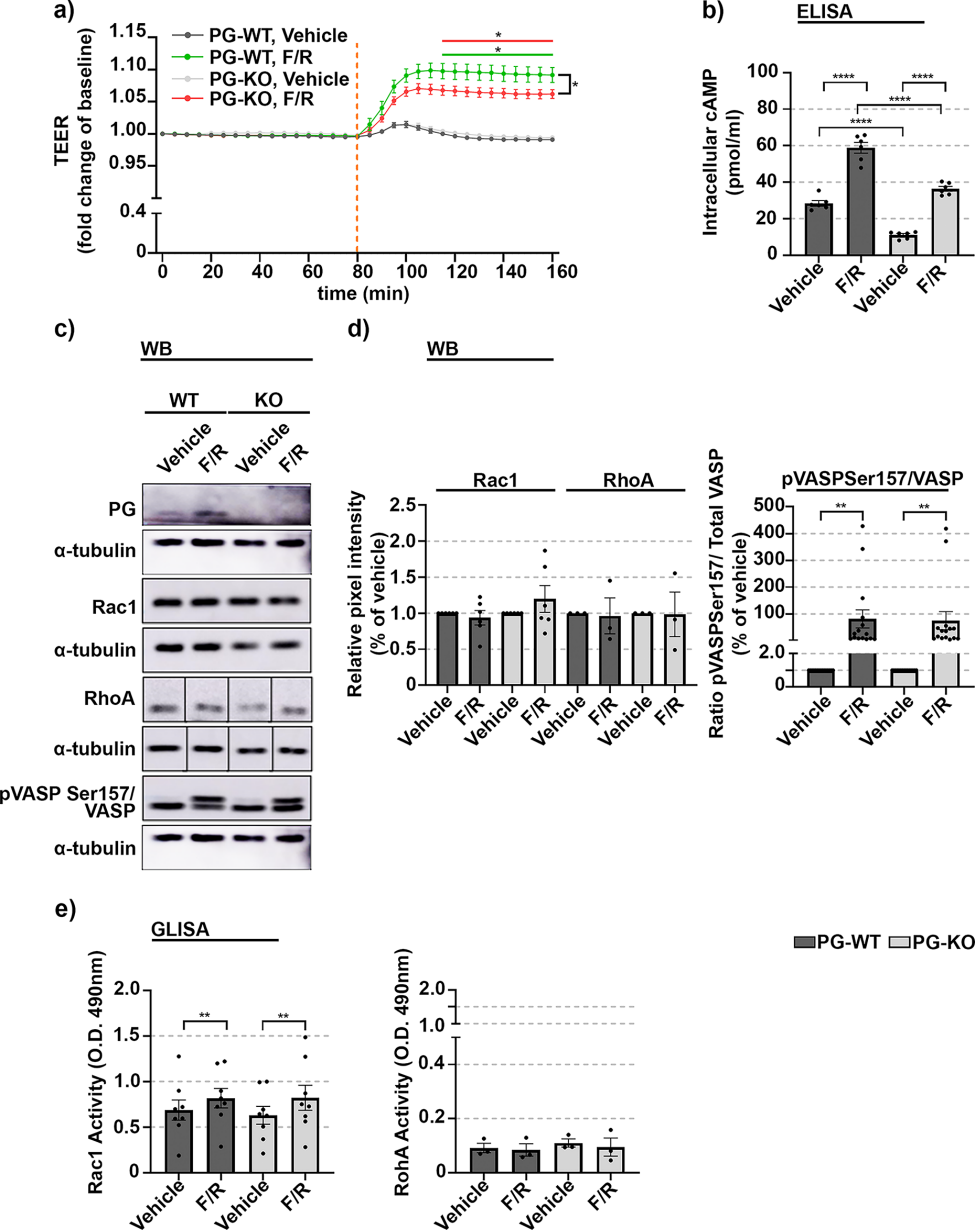
Effect of F/R-mediated intracellular cAMP elevation on barrier integrity and activity of members of Rho family of small GTPases in WT and PG-depleted cells. (**a**) Analysis of barrier integrity of WT and PG-KO cells exposed to vehicle or F/R. The segmented orange line indicates the time of mediator application. “*” underlined with green or red line denotes the time window of significant difference in TEER between Vehicle and F/R-treated monolayers in WT or PG-KO, respectively, *N* = 8. (**b**) Bar diagram displaying the intracellular cAMP levels after 1 h of treatment, assessed by ELISA; *N* = 6 (**c**) Western blot analysis visualizing the relative protein expression of Rac1 and RhoA in WT and PG-KO cells treated either with vehicle or F/R. α-tubulin was used to charge equal loading. Non-contiguous RhoA bands originating from the same membrane are distinct by a vertical black line. An equal exposure time was used for detection of the specific bands. Original blots are presented in Figure S6 d, provided in the Supplementary info file; *N* = 3 (**d**) Bar graph representing the respective densitometric measurement of the bands enclosed in (**c**). Within the respective cell line, the expression was reported as relative to Vehicle; to validate effective treatment with F/R, the phosphorylation status of VASP was assessed by building a ratio between phosphorylated and total VASP (sum of phosphorylated- and not phosphorylated- VASP). The data are presented as relative to the respective Vehicle; *N* ≥ 10 (**e**) Modulation of Rac1 and RhoA GTPase activities due to F/R treatment in WT and PG-KO cells, assessed by G-LISA; *N* ≥ 3. Data are presented as mean ± SEM; * *p* ≤ 0,05; ** *p* ≤ 0,01**** *p* ≤ 0,0001.

### cAMP-mediated endothelial barrier enhancement occurs independently of PG

During onset of inflammation, the intracellular levels of cAMP decrease prominently. In contrast, cAMP elevation promotes reduced paracellular permeability and attenuates the inflammatory response of ECs in vitro and in vivo. Our group and others have shown that endothelial barrier integrity improves following rise of intracellular cAMP concentration^42,50,51,56^ and for an extensive overview refer to Vielmuth et al.^42^. To investigate the relevance of PG for cAMP-mediated endothelial barrier regulation, we performed TEER measurements to track the barrier function over the time, in confluent WT and PG-KO cell monolayers treated with DMSO as a vehicle or with the combination of the AC activator- Forskolin (F) and the PDE4 inhibitor- Rolipram (R), to increase the intracellular cAMP levels. The analysis revealed that both cell lines respond to F/R-mediated cAMP elevation with increased TEER, however, the effect was less prominent in cells without PG, indicating that PG is required to certain extent for cAMP-mediated tightening of the barrier, but it is not dispensable in this process (Fig. 4a). Similarly, in both WT and PG-KO cells, a significant increase in cytosolic cAMP content was observed following F/R application, however, the effect was less noticeable in cells devoid of PG (Fig. 4b). On the other hand, the protein levels of Rac1 and RhoA signaling molecules were unaltered by the treatment (Fig. 4c and d). To validate the efficiency of F/R treatment, the phosphorylation of VASP, a cAMP/PKA-dependent effector was analyzed. Both cell lines reacted similarly, by significantly increasing the phosphorylation of VASP due to treatment (Fig. 4c and d), indicating not only that F/R application is sufficient, but also indirectly, that F/R-mediated PKA signaling is independent of PG. Moreover, in both cell lines, the cAMP- mediated tightening of endothelial barrier was associated with increased Rac1 activation (Fig. 4e). No modulation on RhoA activity was observed as a consequence of F/R application (Fig. 4e).

**Fig. 5:**
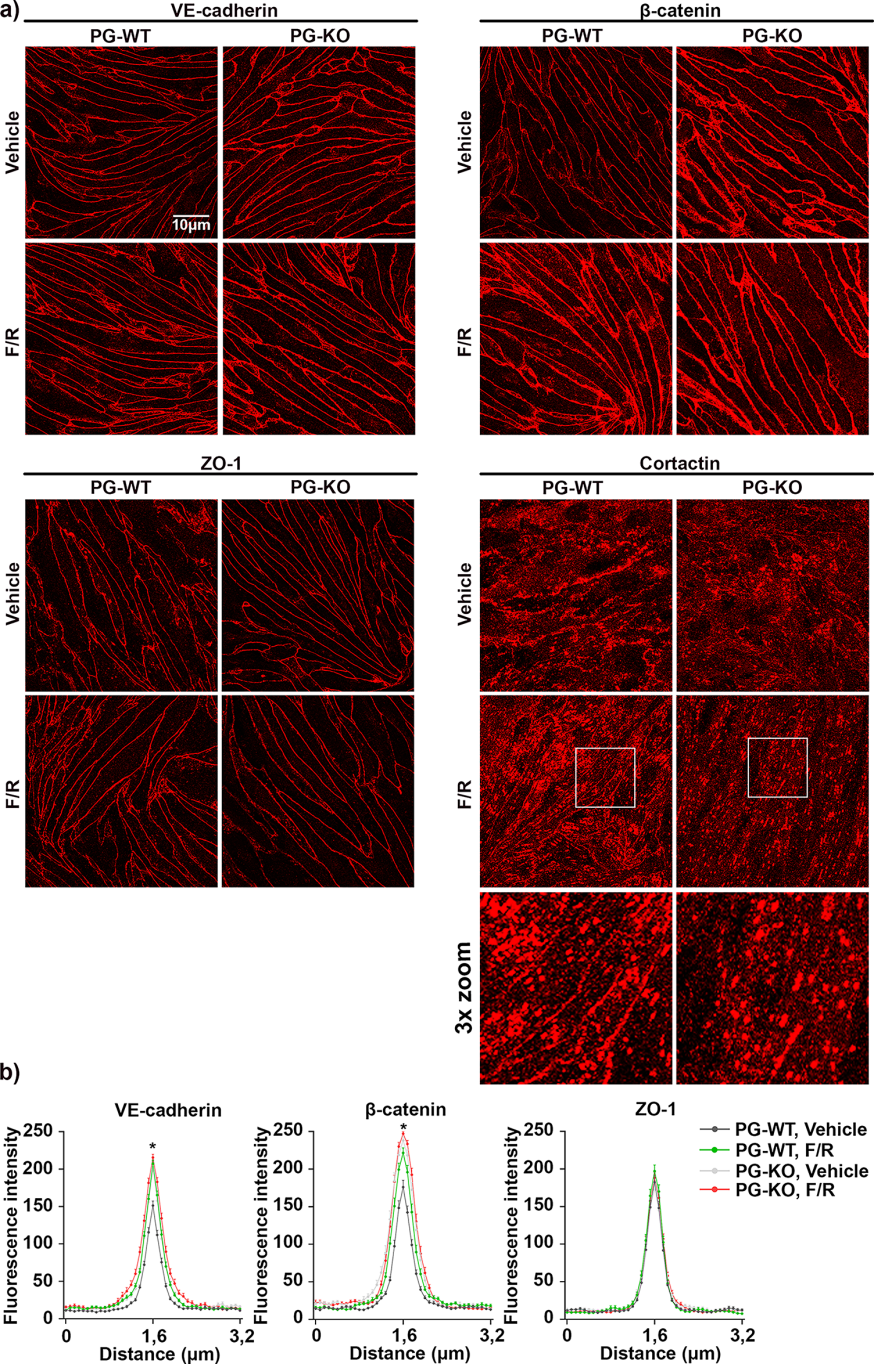
Localization pattern of junctional and junctional-related proteins after cAMP elevation in WT and PG-KO cells. (**a**) Representative immunostaining for VE-cadherin, β-catenin, ZO-1 and cortactin in confluent monolayers of WT and PG-KO cells either subjected to Vehicle or F/R; *N* ≥ 3 (**b**) Bell-shaped curve depicted the pixel intensity of the specific signals across cell-cell contact for VE-cadherin, β-catenin and ZO-1 in treated cells with and without PG; *N* ≥ 3. The “*” for VE-cadherin and β-catenin, symbolized the significant difference in the pixel intensity detected between vehicle and F/R -treated WT monolayers; *N* ≥ 3. * *p* ≤ 0,05.

**Fig. 6:**
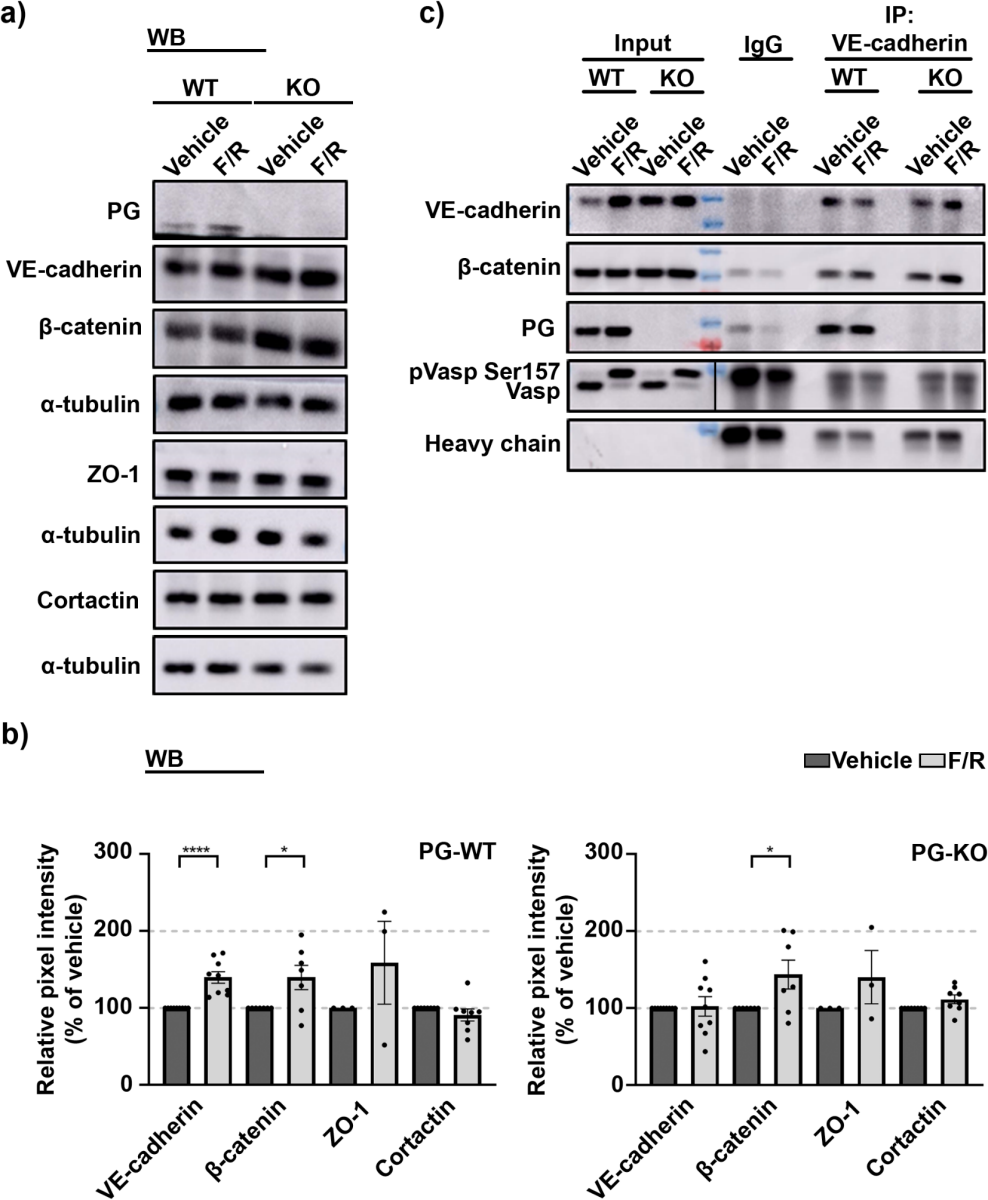
Expression pattern of structural proteins and VE-cadherin-based complex composition in WT and PG-KO cells before and after cAMP elevation. (**a**) Representative Western blots in WT and PG-KO cells, showing the protein expression of VE-cadherin, β-catenin, ZO-1 and cortactin upon cAMP elevation. α-tubulin was used as a loading control; *N* ≥ 3. (**b**) Bar diagrams illustrating the densitometric analysis from (**a**) in WT and PG-KO cells, respectively. The bars reflect the protein abundance in F/R treated cells, when compared to cells exposed to Vehicle. (**c**) VE-cadherin pull-down in WT and PG-KO cells treated either with Vehicle or F/R;phosphorylation status of VASP confirmed effective treatment with F/R; Bands originated from the same membrane, but with different exposure time applied, were separated with a vertical black line. Original blots are presented in Figure S8 a, provided in the Supplementary info file; *N* = 6. Data are presented as mean ± SEM; unpaired t-test; * *p* ≤ 0,05; ** *p* ≤ 0,01; **** *p* ≤ 0,0001.

Next, we explored the protein dynamics of junctional and junctional associated proteins after cAMP elevation. In WT-cells, F/R addition resulted to significant translocation of VE-cadherin and β-catenin towards the junctions. In contrast, cells missing PG exhibited no evident changes in the membrane deposition of both analyzed proteins (Fig. 5a and b). The localization of ZO-1 remained unchanged, either because of lack of PG, or due to the treatment (Fig. 5a and b). However, we observed shifts in the translocation of cortactin, not only due to the loss of PG but also because of F/R treatment. Thus, in WT cells we noticed more cortactin specific signal at the perijunctional area after F/R. This effect was diminished in PG-KO cells (Fig. 5a). Importantly, in WT cells, the changes in the signal intensity of the structural proteins matched the Western blot data. Here, a significant increase in the total protein amount for VE-cadherin and β-catenin after F/R application was detected. Densitometric measurements confirmed that no significant changes for ZO-1 and cortactin were present in either of the cell lines tested (Fig. 6a and b). Moreover, in PG-KO cells, no significant changes in the VE-cadherin protein expression following treatments were observed, and this effect reflected the immunostaining analysis. However, despite the lack of changes in β-catenin localization pattern (Fig. 5a-b), considerable F/R-mediated up-regulation for β-catenin expression in PG-KO cells was found (Fig. 6a and b).

### The association between VE-cadherin and β-catenin does not require PG

β-catenin and PG have many common protein partners, leading up to the speculation that they both can compete for their association with various molecules^38^. Thus, it is widely accepted that both catenins interact with VE-cadherin, therefore, we investigated if the lack of PG induced modulation of the VE-cadherin-β-catenin complex. Immunoprecipitation studies revealed that in both cell lines, neither the treatment with F/R nor the ablation of PG altered the composition of the complex, indicating that the association between VE-cadherin and β-catenin is independent of PG (Fig. 6c).

### The phosphorylation of PG at position Ser665 is not required for cAMP-enhanced adhesion in endothelium

**Fig. 7:**
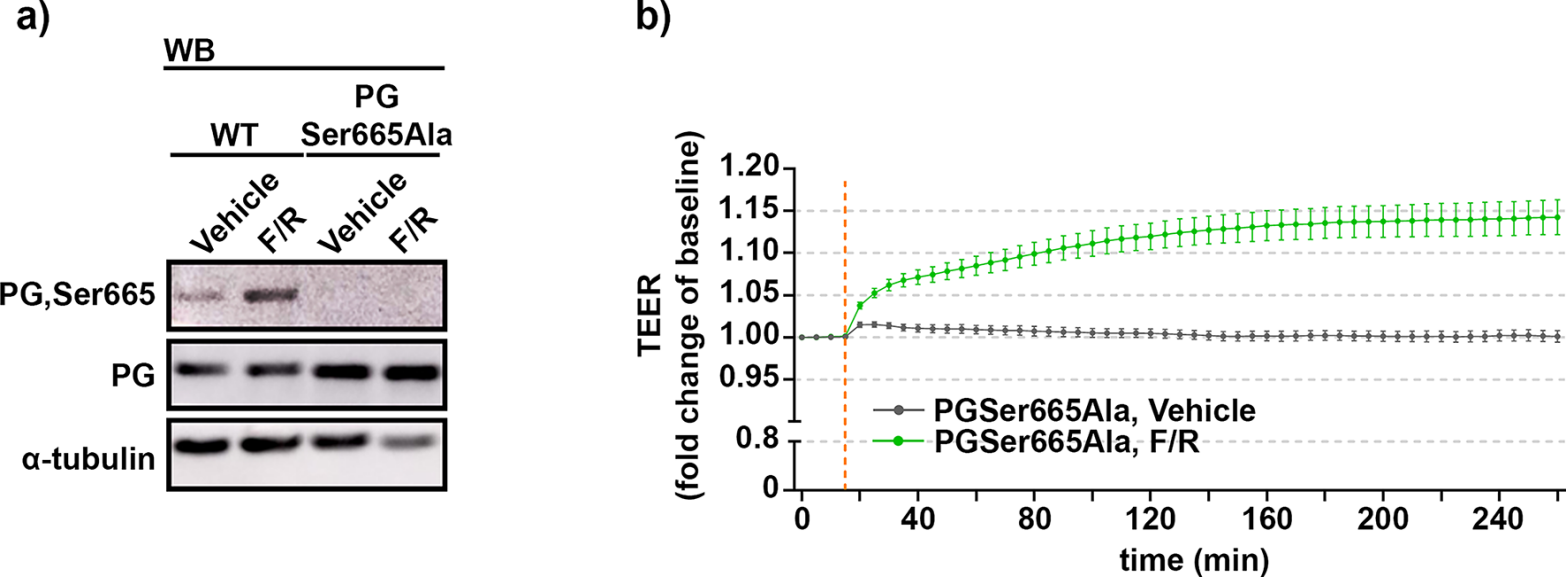
Importance of PG Ser665 phosphorylation site for cAMP-mediated endothelial barrier stability. (**a**) Western blot analysis, in WT and PGSer665Ala mutant cells, with specific antibody recognizing the phosphorylated form of PG at position Ser665, i.e. PGSer665. Cell monolayers were either treated or not with F/R; α-tubulin was used for equal loading; *N* = 4. (**b**) TEER-based barrier integrity measurements in PgSer665Ala confluent monolayers exposed to Vehicle or F/R-treatment; *N* = 6. Data are presented as mean ± SEM.

The phosphorylation of PG at Ser665 has been previously reported to be essential for cAMP- mediated adhesion strengthening in various cell types^44,45,57^. Therefore, phospho-PG deficient endothelial cells derived from PGSer665Ala knock-in mice were established. In the mutant cells, the substitution of Ser665 for Ala prevents the phosphorylation at this position. Thus, in mutant cells, by using previously validated phospho-specific antibody^44,45^, we were not able to confirm phosphorylation of PG under all experimental conditions tested. Importantly, in WT cells, elevation of intracellular cAMP by F/R resulted in higher phosphorylation of PG at Ser665 when compared to cells under control conditions (Fig. 7a). Furthermore, TEER measurement of barrier integrity of the PGSer665Ala monolayers, exposed to vehicle or F/R, revealed that the cAMP-mediated increase in barrier integrity was not blunted (Fig. 7b), indicating that PG phosphorylation at Ser665 plays no critical role in cAMP-mediated endothelial barrier tightening.

## Discussion

With the present study we aimed to better understand the role of PG in basal and cAMP-mediated conditions in heart-derived endothelial cells. Here, we show that loss of PG led to enhanced endothelial integrity (Fig. 1a). The improvement was associated with pronounced translocation of VE-cadherin and β-catenin towards the junctions (Fig. 1b-c) and their increased cellular protein abundance (Fig. 2a and c), suggesting the presence of compensatory mechanism in cells lacking PG. In contrast, it was shown that complete depletion of PG is indeed deadly for mutant embryos^58^. Furthermore, increased permeability to FITC dextran was reported in HUVEC transfected with PG-specific siRNA. This effect correlated with disturb junctional localization of VE-cadherin, thereby is speculated that PG contributes to VE-cadherin dependent adhesion^27^. However, no compensatory increase of VE-cadherin and β-catenin expression were noted as observed in our MyEnd cells. Our findings align with a previous study, where cardio-restricted depletion of PG led to an increase in β-catenin expression at the intercalated disc, which the authors speculated may enhance the function of the remaining gap junctions. However, β-catenin was unable to compensate the missing desmosomal PG^33^. Ultrastructural analyses of hearts, derived from mice with PG restricted cardiomyocyte also revealed intact AJs, where β-catenin was more at junctions and substituted for the missing PG^59^. In this line of thoughts, a compensatory replacement of β-catenin in skin and intestinal desmosomes of PG null mice was observed, but no β-catenin upregulation was evident. Thus, it is important to note that β-catenin at junction suggests less intact β-catenin signaling and consequently disturbed endothelial barrier function. However, in our PG-KO cells, we not only observed more β-catenin at junction, but also higher total protein amount, which does not rule out the possibility of active β-catenin signaling. Moreover, we could not see any substantial differences either in the protein abundance nor in the cellular localization pattern of ZO-1 due to lack of PG (Figs. 1b-c and 2a and d). These observations are in line with a previous study, where PG overexpression in HMEC-1 cells did not appear to change the ZO-1 abundance or localization pattern^30^. Additionally, Tornavaca et al. reported that in HDMEC, a functional unit is formed between the junctional adaptor protein ZO-1 and JAM-A, which may regulate TJ assembly. Thus, it was shown that both proteins not only control their localization at intercellular contacts reciprocally but also are essential for TJ localization of claudin-5. Moreover, depletion of either ZO-1 or JAM-A led to modulation of processes critical for endothelial barrier function, such as an actin cytoskeleton reorganization and actomyosin contractility via ROCK^60^. A recent study also verified the role of JAM-A to increase claudin-5 expression and reported that endothelial barrier function is promoted via JAM-A/EPAC/c/EBP-α signaling, which indeed controls claudin-5 expression^61^. Nevertheless, PG-KO cells had less claudin-5 transcript while exhibiting better barrier integrity when compared to WT cells (Supplementary Figure 3 c-d), indicating that in our working model, the mechanisms controlling barrier function are, with high probability, JAM-A-independent. Similar to others ^62^, we speculated that PECAM-1, another member of the JAM family, may substitute JAM-A in the context of junction organization; since both proteins have been shown to share many structural and functional properties and may exert, in some conditions, redundant roles. Interestingly, we detected an elevated protein expression of PECAM-1 in PG-KO cells, an effect which may contribute to the enhanced TEER observed in these cells (Supplementary Figure 3a-b). Our observation is fitting to the plethora of studies demonstrating the role of PECAM-1 in maintenance of endothelial barrier integrity (summarized in the review by Privratsky, et al., 2014^63^). Thus, it was shown that in contrast to PECAM-1 deficient cells, resting monolayers of PECAM-1 positive endothelial cells have increased steady-state barrier function and more rapid restoration of the barrier integrity when challenged with thrombin^64^. Moreover, while AJs are important for the assembly of TJs^65^, PECAM-1 has been reported to interfere with the expression and localization pattern of VE-cadherin and β-catenin. Thus, studies disclosed that in PECAM-1-null endothelial cells the expression levels of these AJ components were reduced, and this was associated with diminished VE-cadherin junctional localization^66,67^. These observations support our findings indicating that increased expression of PECAM-1 in PG-KO cells is associated with improved barrier function.

Although, claudin-5 is recognized as an endothelial-specific marker, it is most predominant in brain endothelial cells, where it is essential for the size-selective barrier function of TJs within the blood-brain barrier^68–70^. In addition the presence of claudin-1 in endothelial cells has also been recognized^54 ^and associated with the maintenance of endothelial barrier function^71,72^. Furthermore, several studies revealed a correlation between claudin-1 and − 5 expressions^73,74^. Similarly, we also noticed a link between the expression of these two claudins. Thus, while claudin-5 mRNA was significantly down-regulated in PG-KO, claudin-1 mRNA was profoundly upregulated, an effect detected at the protein level too, although not significant (Supplementary Fig. 3a-d). With the current results, we can only speculate that a claudin-1-dependent compensatory mechanism may be involved in the maintenance of endothelial barrier integrity when PG is absent, however, more detailed analysis is needed to further elucidate this theory. Additionally, previous research has suggested that PG expression significantly impacts actin organization^43^. However, our assessment revealed no significant variations in the F-actin staining when WT and PG-KO cells were compared (Supplementary Fig. 1a), indicating that PG does not play a critical role in actin cytoskeleton reorganization.

Recently, cortactin was also found in complex with VE-cadherin, β-catenin and PG and lack of cortactin disrupted the membrane distribution of VE-cadherin and β-catenin^51^, opposing our current findings with PG-KO cells. Furthermore, differential expression of cortactin was not induced by PG ablation or vice versa (Fig. 2a-d). Despite this, here we show that lack of PG prevented the translocation of cortactin towards cell-cell contacts, independently of the unaltered protein or mRNA abundance both under resting conditions and after cAMP increase (Figs. 1b, 2 and a-d, 5a and 6a-b). Moreover, in WT cells, the junctional translocation of cortactin was more noticeable after F/R-facilitated cAMP elevation (Fig. 5a), confirming its role in endothelial barrier function ^51^. Our observations align with a previous study highlighting the interplay between cortactin and adrenomedullin, which is known to stimulate cAMP production^75,76^. In turn, cAMP elevation leads to Rap1-dependent Rac1 activation, previously reported to facilitate cortactin translocation to the cell periphery. Thus, it was shown that cells transfected with constantly active Rac1, presented more cortactin at cell borders where it colocalized with Rac1^77,78^. Therefore, we may speculate that more active Rac1 is engaged at junctions in WT cells, however, the contacts are tighter in PG-KO cells, due to the significant abundance and membrane translocation of VE-cadherin and β-catenin. Indeed, both cortactin and PG affect junctional VE- cadherin distribution and thus, endothelial barrier integrity.

In this study, we also reported that in the absence of PG, the levels of the intracellular cAMP drop down significantly (Fig. 2h). It is well known that both ACs and PDEs are involved in the cAMP homeostasis. While ACs are implicated in generation of cAMP, the PDEs are crucial for tunning the cAMP responses within the cell via degradation of the second messenger. In fact, the balance between the activities of the two enzymes is critical for regulation of the intracellular cAMP levels^42,79^. In this regard, we speculated that due to PG depletion, the spatio-temporal localization of PDE4 and/or AC may change. A possible mechanism to explain our findings may involve signalosomes, a multiprotein complex containing A-kinase anchoring proteins (AKAPs). The latter are found to directly and/or indirectly associate with various cAMP effector molecules such as PKA and Epac, but also are registered to balance the cellular cAMP content by interacting with AC and PDE^80^. In addition, we have previously shown that AKAPs are part of VE-cadherin based complex, together with β-catenin and PKA^81^. Therefore, we hypothesise that loss of PG promotes the present of AKAP-containing signalosomes at junctions where more VE-cadherin and β-catenin accumulated to fine tune the local cAMP pool and improve junctional integrity. Several studies have supported this notion, demonstrating that the membrane localized cAMP pool is associated with improved barrier function, whereas the cytosolic cAMP pool leads to barrier dysfunction (refer to^42^ for detailed references). It is important however, to consider that in this study the assay used to measure the cAMP content cannot distinguish between membrane-bound or cytosolic cAMP, and thus possess a technical limitation to understand this phenomenon in detail. Furthermore, although the total cellular cAMP concentration significantly differs in both cell lines, the enzymatic activity of Rac1 and RhoA was unaltered and no changes in the actin cytoskeleton were visible (Fig. 2g and Supplementary Fig. 1), which indeed is fitting to the concept that junction-associated cortical actin reorganization is maybe controlled by Rho GTPases^82^. Moreover, number of groups reported the relationship between Rho GTPases and Wnt signaling, where β-catenin and PG are well-known players^83–85^. Nonetheless, not many studies exploring the interconnection between PG alterations and Rho GTPase activity are currently available. However, a previous report revealed that Rac1 and RhoA activity are significantly elevated in PG-depleted keratinocytes, relative to heterozygote for PG mutants and this was associated with changes in the actin cytoskeleton organization^43^. These observations contrasted with our analyses suggesting that PG is not crucial for either regulating small Rho GTPase activity or cytoskeleton remodeling. One explanation for this discrepancy can be the use of a different cell types, possessing different sorts of junctions, where other signaling pathways contribute to maintain junctional integrity.

Nottebaum and colleagues reported that VE-PTP associates with VE-cadherin to maintain endothelial barrier function via PG. Interestingly, PG and VE-PTP are hardly detectable at new form contacts. However, when cells approach confluency, PG was shown to be accumulated at junctions where it merged with VE-cadherin^26–28^. Moreover, VE-PTP silencing induced higher permeability and inhibited VE-cadherin-mediated adhesion. However, when PG-deficient endothelioma cells were knocked for VE-PTP, less increased permeability was noticed in comparison to cells possessing endogenous PG. Thus, a possible correlation between PG presence and VE-PTP expression that acts on VE-cadherin-based junctions was recognized^26^. Since PG is also a signaling molecule, it may control mRNA stability and consequently the activity of downstream effectors, as already reported^43^. It was demonstrated that PG ablation led to significant increase of VE-PTP mRNA^27^. This observation did not reflect our finding, where VE-PTP mRNA levels remained similar in both WT and PG-KO cells (Fig. 3d-e). In contrast, possibly because of posttranscriptional or posttranslational modification, we found that in PG-depleted cells, the VE-PTP protein levels were increased, and this effect was accompanied by a prominent reduction of VE-cadherin phosphorylation (Fig. 3a-c). These results are in line with a series of reports providing evidence that tyrosine phosphorylation of various components of VE-cadherin-catenin complex is associated with weaken AJ integrity and impaired barrier function^26,86–91^. Moreover, it was reported that VE-PTP associates with VE-cadherin and control its level of phosphorylation regardless of its enzymatic activities^25^. Thus, it was reported that VE-PTP may serve as an adaptor molecule stabilizing VE-cadherin junctions by reducing the internalization rate of AJs though/via limiting RhoA-mediated tension across junctions^92^. However, in our experimental model, no change in RhoA activity was detected for any of the cell lines tested (Fig. 2g), even though VE-PTP expression levels were altered due to PG loss. Together, our data suggest that PG regulates the phosphorylation state of VE-cadherin and its junctional stability via control over VE-PTP expression. Nevertheless, it is still not clear how this regulation occurs. Moreover, it was already shown that phosphorylation of VE-cadherin is often attributed to VE-PTP dissociation^26,93^, resulting in rapid cadherin internalization and degradation causing endothelial barrier disorganization^94,95^. Due to technical limitations, we could not validate the association between VE-PTP and VE-cadherin to explore any potential alterations in the interaction after PG depletion. Still, we demonstrated that the VE-cadherin-β-catenin complex is not affected in PG-KO cells (Fig. 6c). Thus, we cannot rule out any possibility that a PG-independent compensatory mechanism can be implicated in VE-PTP-VE-cadherin mediated endothelial barrier integrity. We proposed that PG acts as a cellular “sensor” for junctional tightening, controlling the accumulation of structural proteins towards cell-cell contacts. When PG is not present, a disbalance occurs and endothelial cells try to compensate for it by overexpressing and accumulating VE-cadherin and β-catenin at the junctions.

We also investigated the contribution of PG for cAMP-mediated strengthening of endothelial barrier. TEER measurements revealed that both WT and PG-KO cells react to F/R application by elevating resistance. However, the effect was milder in PG-KO cells (Fig. 4a). The F/R-mediated response in integrity correlated with increased levels of cAMP in both cell lines, but once again, the effect in PG-KO cells was less pronounced (Fig. 4b). In this regard, we observed that cAMP elevation leads to accumulation of VE-cadherin and β-catenin at the junctions in WT cells, while no significant changes were observed for PG-KO cells, probably due to already increased translocation of those structural proteins under basal conditions (Figs. 1b-c and 5a-b). The disclosed translocation patterns in WT correlated with more total protein after F/R application, similar to a previously published study ^51^. Despite the trend of increased VE-cadherin and β-catenin expression by F/R in PG-KO cells, no significant alterations in the junctional accumulation of the proteins were observed (Figs. 5a-b and 6a-b). We proposed that the lesser reaction occurred because a pre-fortified barrier of PG-KO may be insensitive to the rise of cAMP, since no further junction tightening is physiologically possible as the molecules involved in this process reach a point of saturation where more cAMP is not necessarily beneficial for the barrier function. In addition, ZO-1 and cortactin did not show any alterations in the protein abundance due to lack of PG or treatment. However, cortactin was more at junctions after F/R treatment in both cell lines, but the effect was less prominent in PG-KO (Figs. 5a-b and 6a-b). We were also able to verify that neither the lack of PG nor the F/R treatment interfered with the composition of the VE-cadherin-β-catenin complex (Fig. 6c), indicating that PG is not critical for this association. Furthermore, the activity of Rac1, but not RhoA was significantly elevated in both cell lines due to treatment, as the effect was comparable despite the pronounced difference in the cAMP content (Fig. 4b and e). Thereby, we are speculating that pathways different than cAMP-mediated signalling can also affect Rac1 activity or PG itself may control spatial and temporal compartmentalization of cAMP and its targets resulting in specific modulation of the intracellular signalling.

A potential relation between PG and the main cAMP downstream effectors, PKA and Epac1 was also examined. We demonstrated that the phosphorylation of VASP, a PKA effector, was not affected by lack of PG or exposure to treatment. Furthermore, similar to other reports^50,96^, the application of Epac1 specific activator, O-Me-cAMP, lead to a moderate, but a significant increase in barrier resistance of both cell lines (Fig. 4c-d and Supplementary Fig. 2a-c). Thus, we concluded that PG neither contributes nor interferes with PKA and Epac1 function following cAMP stimulation.

Several studies showed that a rise of cAMP concentration triggers an increase of adhesive cohesion in different cell types and this effect correlates with profound phosphorylation of PG at Ser665^44,45,57^. Therefore, by using TEER measurements, we evaluated the monolayer integrity in phosphodeficient for PG Ser665 endothelium exposed to high intracellular levels of cAMP. In contrast to the already published data, we could demonstrate a pronounced increase in barrier resistance of PGSer665Ala monolayers exposed to treatment (Fig. 7b), speculating that the phosphorylation of PG at Ser665 is not relevant for endothelial barrier tightening upon cAMP stimulation.

In summary, PG acts as a regulator of cellular adhesion and barrier homeostasis in different cell types, ranging from cardiomyocytes to endothelial cells. Especially in the endothelium, PG takes the center stage by modulating junctional integrity and the expression of diverse barrier components, as it is well-known that this protein functions as a structural and signaling molecule. We have demonstrated that the lack of PG modulates AJ and TJ by influencing VE-cadherin, β-catenin, claudins and PECAM-1. Part of the effect on AJ may be explained by the PG-dependent modulation of VE-PTP expression and subsequent junctional accumulation of VE-cadherin, leading to improved barrier function. On the other hand, the loss of PG had no influence on small GTPases despite altering the intracellular levels of cAMP. The aforementioned effects could be due to compensatory mechanisms leading to enhanced barrier in PG-KO cells, which may not be benefited by an additional enhancement of the intracellular cAMP.

Thus, the loss of PG could lead to multiple consequences: on one hand, PG possibly acts upstream of transcription factors controlling the mRNA expression of structural or signaling molecules; and on the other hand, PG could alter the activity of signaling molecules involved in barrier-mediating pathways. In conclusion, PG may act as a sensor to regulate endothelial junctional turn-over and thereby barrier integrity.

## Electronic supplementary material

Below is the link to the electronic supplementary material.

Supplementary Material 1

Supplementary Material 2

Supplementary Material 3

Supplementary Material 4

## Data Availability

The datasets used and/or analyzed during the current study are available from the corresponding author on reasonable request.

## Abbreviations

PG: Plakoglobin
cAMP: cyclic adenosine monophosphate
KO: Knock-out
WT: Wild type
TEER: Transendothelial electrical resistance
VE-cadherin: Vascular endothelial cadherin
VE-PTP: Vascular endothelial protein tyrosine phosphatase
Rac1: Ras-related C3 botulinium toxin substrate 1 (Rho Family, Small GTP Binding Protein Rac1)
RhoA: Ras homolog family member A
TJ: Tight junction
AJ: Adherens junction
AC: Adenylyl cyclase
ZO: Zonula occludens
VEGFR2: Vascular endothelial growth factor receptor 2
TGF-βRII/I: Transforming growth factor beta receptor II/I
DEP-1: Density-enhanced phosphatase 1
mRNA: messenger ribonucleic acid
HDMEC-1: Human dermal microvascular endothelial cell
GTP: Guanosine triphosphate
ECs: Endothelial cells
siRNA: small interfering ribonucleic acid
PCR: Polymerase chain reaction
MyEnd: Myocardial endothelial
HEPES: 4-(2-hydroxyethyl)-1-piperazineethanesulfonic acid
BSA: Bovine serum albumin
DMEM: Dulbecco’s Modified Eagle’s medium
FCS: Fetal calf serum
vWF: von Willebrand Factor
PECAM-1: Platelet and endothelial cell adhesion molecule 1
F/R: Forskolin/Rolipram
DMSO: Dimethyl sulfoxide
Epac-1: Exchange protein directly activated by cAMP 1
O-Me-cAMP: O-methyladenosine-cyclic monophosphate
PBS: Phosphate-buffered saline
SDS: Sodium dodecyl-sulfate
Ser: Serine
EDTA: Ethylenediaminetetraacetic
TBS-T: Tris-Buffered Saline, containing 0,1% Tween
HRP: Horseradish Peroxidase
ECL: Enhanced chemiluminescence
IP: Immunoprecipitation
DAPI: 4’,6-diamino-2-phenylindole
cDNA: complementary deoxyribonucleic acid
RNA: Ribonucleic acid
dNTP: deoxy nucleotide triphosphate
B2M: Beta-2-microtubulin
GLISA: Small GTPase activation assay
ELISA: Enzyme-linked immunosorbent assay
VASP: Vasodilator-stimulated phosphoprotein
PKA: Protein kinase A
PDE: Phosphodiesterase
AKAP: A-kinase anchoring protein

## References

[1] Wettschureck, N., Strilic, B. & Offermanns, S. Passing the vascular barrier: endothelial signaling processes controlling extravasation. *Physiol. Rev.* **99**, 1467–1525. 10.1152/physrev.00037.2018 (2019). 31140373 10.1152/physrev.00037.2018

[2] Dejana, E., Orsenigo, F., Molendini, C., Baluk, P. & McDonald, D. M. Organization and signaling of endothelial cell-to-cell junctions in various regions of the blood and lymphatic vascular trees. *Cell. Tissue Res.* **335**, 17–25. 10.1007/s00441-008-0694-5 (2009). 18855014 10.1007/s00441-008-0694-5 PMC4422058

[3] Schnittler, H. et al. Actin filament dynamics and endothelial cell junctions: the Ying and Yang between stabilization and motion. *Cell. Tissue Res.* **355**, 529–543. 10.1007/s00441-014-1856-2 (2014). 24643678 10.1007/s00441-014-1856-2

[4] Bravi, L., Dejana, E. & Lampugnani, M. G. VE-cadherin at a glance. *Cell. Tissue Res.* **355**, 515–522. 10.1007/s00441-014-1843-7 (2014). 24643676 10.1007/s00441-014-1843-7

[5] Nelson, W. J. Regulation of cell-cell adhesion by the cadherin-catenin complex. *Biochem. Soc. Trans.* **36**, 149–155. 10.1042/BST0360149 (2008). 18363555 10.1042/BST0360149 PMC3368607

[6] Peifer, M., McCrea, P. D., Green, K. J., Wieschaus, E. & Gumbiner, B. M. The vertebrate adhesive junction proteins beta-catenin and Plakoglobin and the drosophila segment Polarity gene armadillo form a multigene family with similar properties. *J. Cell. Biol.* **118**, 681–691. 10.1083/jcb.118.3.681 (1992). 1639851 10.1083/jcb.118.3.681 PMC2289544

[7] Butz, S., Stappert, J., Weissig, H. & Kemler, R. Plakoglobin and beta-catenin: distinct but closely related. *Science* **257**, 1142–1144. 10.1126/science.257.5073.1142-a (1992). 1509266 10.1126/science.257.5073.1142-a

[8] McCrea, P. D., Turck, C. W. & Gumbiner, B. A homolog of the armadillo protein in drosophila (plakoglobin) associated with E-cadherin. *Science* **254**, 1359–1361. 10.1126/science.1962194 (1991). 1962194 10.1126/science.1962194

[9] Rubenstein, A., Merriam, J. & Klymkowsky, M. W. Localizing the adhesive and signaling functions of Plakoglobin. *Dev. Genet.* **20**, 91–102. https://doi.org/10.1002/(SICI)1520-6408(1997)20:2<91::AID-DVG2>3.0.CO;2-3 (1997). 10.1002/(SICI)1520-6408(1997)20:2<91::AID-DVG2>3.0.CO;2-3 9144920

[10] Willert, K. & Nusse, R. Beta-catenin: a key mediator of Wnt signaling. *Curr. Opin. Genet. Dev.* **8**, 95–102. 10.1016/s0959-437x(98)80068-3 (1998). 9529612 10.1016/s0959-437x(98)80068-3

[11] Huber, A. H., Nelson, W. J. & Weis, W. I. Three-dimensional structure of the armadillo repeat region of beta-catenin. *Cell* **90**, 871–882. 10.1016/s0092-8674(00)80352-9 (1997). 9298899 10.1016/s0092-8674(00)80352-9

[12] Bullions, L. C. & Levine, A. J. The role of beta-catenin in cell adhesion, signal transduction, and cancer. *Curr. Opin. Oncol.* **10**, 81–87. 10.1097/00001622-199801000-00013 (1998). 9466489 10.1097/00001622-199801000-00013

[13] Hakimelahi, S. et al. Plakoglobin regulates the expression of the anti-apoptotic protein BCL-2. *J. Biol. Chem.* **275**, 10905–10911. 10.1074/jbc.275.15.10905 (2000). 10753888 10.1074/jbc.275.15.10905

[14] Xu, W. & Kimelman, D. Mechanistic insights from structural studies of beta-catenin and its binding partners. *J. Cell. Sci.* **120**, 3337–3344. 10.1242/jcs.013771 (2007). 17881495 10.1242/jcs.013771

[15] Ozawa, M., Baribault, H. & Kemler, R. The cytoplasmic domain of the cell adhesion molecule Uvomorulin associates with three independent proteins structurally related in different species. *Embo J.* **8**, 1711–1717. 10.1002/j.1460-2075.1989.tb03563.x (1989). 2788574 10.1002/j.1460-2075.1989.tb03563.x PMC401013

[16] Knudsen, K. A., Soler, A. P., Johnson, K. R. & Wheelock, M. J. Interaction of alpha-actinin with the Cadherin/catenin cell-cell adhesion complex via alpha-catenin. *J. Cell. Biol.* **130**, 67–77. 10.1083/jcb.130.1.67 (1995). 7790378 10.1083/jcb.130.1.67 PMC2120515

[17] Rimm, D. L., Koslov, E. R., Kebriaei, P., Cianci, C. D. & Morrow, J. S. Alpha 1(E)-catenin is an actin-binding and -bundling protein mediating the attachment of F-actin to the membrane adhesion complex. *Proc. Natl. Acad. Sci. U S A*. **92**, 8813–8817. 10.1073/pnas.92.19.8813 (1995). 7568023 10.1073/pnas.92.19.8813 PMC41057

[18] Weiss, E. E., Kroemker, M., Rudiger, A. H., Jockusch, B. M. & Rudiger, M. Vinculin is part of the cadherin-catenin junctional complex: complex formation between alpha-catenin and vinculin. *J. Cell. Biol.* **141**, 755–764. 10.1083/jcb.141.3.755 (1998). 9566974 10.1083/jcb.141.3.755 PMC2132754

[19] Itoh, M., Nagafuchi, A., Moroi, S. & Tsukita, S. Involvement of ZO-1 in cadherin-based cell adhesion through its direct binding to alpha Catenin and actin filaments. *J. Cell. Biol.* **138**, 181–192 (1997). 9214391 10.1083/jcb.138.1.181 PMC2139940

[20] Pradhan, D., Lombardo, C. R., Roe, S., Rimm, D. L. & Morrow, J. S. Alpha -Catenin binds directly to spectrin and facilitates spectrin-membrane assembly in vivo. *J. Biol. Chem.* **276**, 4175–4181. 10.1074/jbc.M009259200 (2001). 11069925 10.1074/jbc.M009259200

[21] Dejana, E., Tournier-Lasserve, E. & Weinstein, B. M. The control of vascular integrity by endothelial cell junctions: molecular basis and pathological implications. *Dev. Cell.* **16**, 209–221. 10.1016/j.devcel.2009.01.004 (2009). 19217423 10.1016/j.devcel.2009.01.004

[22] Broman, M. T. et al. Cdc42 regulates adherens junction stability and endothelial permeability by inducing alpha-catenin interaction with the vascular endothelial Cadherin complex. *Circ. Res.* **98**, 73–80. 10.1161/01.RES.0000198387.44395.e9 (2006). 16322481 10.1161/01.RES.0000198387.44395.e9

[23] Gavard, J. Endothelial permeability and VE-cadherin: a wacky comradeship. *Cell. Adh Migr.* **7**, 455–461. 10.4161/cam.27330 (2013). 24430214 10.4161/cam.27330 PMC3916348

[24] Gavard, J. Endothelial permeability and VE-cadherin: a wacky comradeship. *Cell. Adh Migr.* **8**, 158–164 (2014). 25422846 10.4161/cam.29026 PMC4049861

[25] Nawroth, R. et al. VE-PTP and VE-cadherin ectodomains interact to facilitate regulation of phosphorylation and cell contacts. *Embo J.* **21**, 4885–4895 (2002). 12234928 10.1093/emboj/cdf497 PMC126293

[26] Nottebaum, A. F. et al. VE-PTP maintains the endothelial barrier via Plakoglobin and becomes dissociated from VE-cadherin by leukocytes and by VEGF. *J. Exp. Med.* **205**, 2929–2945. 10.1084/jem.20080406 (2008). 19015309 10.1084/jem.20080406 PMC2585844

[27] Muramatsu, F. et al. Plakoglobin maintains the integrity of vascular endothelial cell junctions and regulates VEGF-induced phosphorylation of VE-cadherin. *J. Biochem.* **162**, 55–62. 10.1093/jb/mvx001 (2017). 28158602 10.1093/jb/mvx001

[28] Lampugnani, M. G. et al. The molecular organization of endothelial cell to cell junctions: differential association of Plakoglobin, beta-catenin, and alpha-catenin with vascular endothelial Cadherin (VE-cadherin). *J. Cell. Biol.* **129**, 203–217 (1995). 7698986 10.1083/jcb.129.1.203 PMC2120375

[29] Schnittler, H. J., Puschel, B. & Drenckhahn, D. Role of cadherins and Plakoglobin in interendothelial adhesion under resting conditions and shear stress. *Am. J. Physiol.* **273**, H2396–2405. 10.1152/ajpheart.1997.273.5.H2396 (1997). 9374777 10.1152/ajpheart.1997.273.5.H2396

[30] Venkiteswaran, K. et al. Regulation of endothelial barrier function and growth by VE-cadherin, Plakoglobin, and beta-catenin. *Am. J. Physiol. Cell. Physiol.* **283**, C811–821. 10.1152/ajpcell.00417.2001 (2002). 12176738 10.1152/ajpcell.00417.2001

[31] Cox, R. T. et al. Membrane-tethered drosophila armadillo cannot transduce wingless signal on its own. *Development* **126**, 1327–1335 (1999). 10021350 10.1242/dev.126.6.1327

[32] Salomon, D. et al. Regulation of beta-catenin levels and localization by overexpression of Plakoglobin and Inhibition of the ubiquitin-proteasome system. *J. Cell. Biol.* **139**, 1325–1335. 10.1083/jcb.139.5.1325 (1997). 9382877 10.1083/jcb.139.5.1325 PMC2140206

[33] Li, J. et al. Cardiac tissue-restricted deletion of Plakoglobin results in progressive cardiomyopathy and activation of {beta}-catenin signaling. *Mol. Cell. Biol.* **31**, 1134–1144. 10.1128/MCB.01025-10 (2011). 21245375 10.1128/MCB.01025-10 PMC3067899

[34] Garcia-Gras, E. et al. Suppression of canonical Wnt/beta-catenin signaling by nuclear Plakoglobin recapitulates phenotype of arrhythmogenic right ventricular cardiomyopathy. *J. Clin. Invest.* **116**, 2012–2021. 10.1172/JCI27751 (2006). 16823493 10.1172/JCI27751 PMC1483165

[35] Aktary, Z. et al. Plakoglobin interacts with and increases the protein levels of metastasis suppressor Nm23-H2 and regulates the expression of Nm23-H1. *Oncogene* **29**, 2118–2129. 10.1038/onc.2009.495 (2010). 20101217 10.1038/onc.2009.495

[36] Shimizu, M., Fukunaga, Y., Ikenouchi, J. & Nagafuchi, A. Defining the roles of beta-catenin and Plakoglobin in LEF/T-cell factor-dependent transcription using beta-catenin/plakoglobin-null F9 cells. *Mol. Cell. Biol.* **28**, 825–835. 10.1128/MCB.02375-06 (2008). 17984222 10.1128/MCB.02375-06 PMC2223424

[37] Zhurinsky, J. & Shtutman, M. Ben-Ze’ev, A. Differential mechanisms of LEF/TCF family-dependent transcriptional activation by beta-catenin and Plakoglobin. *Mol. Cell. Biol.* **20**, 4238–4252. 10.1128/MCB.20.12.4238-4252.2000 (2000). 10825188 10.1128/mcb.20.12.4238-4252.2000 PMC85792

[38] Zhurinsky, J. & Shtutman, M. Ben-Ze’ev, A. Plakoglobin and beta-catenin: protein interactions, regulation and biological roles. *J. Cell. Sci.* **113** (Pt 18), 3127–3139 (2000). 10954412 10.1242/jcs.113.18.3127

[39] Teuliere, J. et al. beta-catenin-dependent and -independent effects of DeltaN-plakoglobin on epidermal growth and differentiation. *Mol. Cell. Biol.* **24**, 8649–8661. 10.1128/MCB.24.19.8649-8661.2004 (2004). 15367683 10.1128/MCB.24.19.8649-8661.2004 PMC516731

[40] Maeda, O. et al. Plakoglobin (gamma-catenin) has TCF/LEF family-dependent transcriptional activity in beta-catenin-deficient cell line. *Oncogene* **23**, 964–972. 10.1038/sj.onc.1207254 (2004). 14661054 10.1038/sj.onc.1207254

[41] McCrea, P. D., Gu, D. & Balda, M. S. Junctional music that the nucleus hears: cell-cell contact signaling and the modulation of gene activity. *Cold Spring Harb Perspect. Biol.* **1**, a002923. 10.1101/cshperspect.a002923 (2009). 20066098 10.1101/cshperspect.a002923 PMC2773621

[42] Vielmuth, F. et al. A master regulator of cadherin-mediated binding in endothelium, epithelium and myocardium. *Acta Physiol. (Oxf)*. **238**, e14006. 10.1111/apha.14006 (2023). 37243909 10.1111/apha.14006

[43] Todorovic, V. et al. Plakoglobin regulates cell motility through Rho- and fibronectin-dependent Src signaling. *J. Cell. Sci.* **123**, 3576–3586. 10.1242/jcs.070391 (2010). 20876660 10.1242/jcs.070391 PMC2951470

[44] Sigmund, A. M. et al. Apremilast prevents blistering in human epidermis and stabilizes keratinocyte adhesion in pemphigus. *Nat. Commun.* **14**, 116. 10.1038/s41467-022-35741-0 (2023). 36624106 10.1038/s41467-022-35741-0 PMC9829900

[45] Yeruva, S. et al. Adrenergic Signaling-Induced ultrastructural strengthening of intercalated discs via Plakoglobin is crucial for positive adhesiotropy in murine cardiomyocytes. *Front. Physiol.* **11**, 430. 10.3389/fphys.2020.00430 (2020). 32508670 10.3389/fphys.2020.00430 PMC7253624

[46] Schinner, C. et al. Adrenergic signaling strengthens cardiac myocyte cohesion. *Circ. Res.*10.1161/CIRCRESAHA.116.309631 (2017). 28289018 10.1161/CIRCRESAHA.116.309631

[47] Burek, M., Salvador, E. & Forster, C. Y. Generation of an immortalized murine brain microvascular endothelial cell line as an in vitro blood brain barrier model. *J Vis Exp*, e4022 (2012). 10.3791/4022 10.3791/4022 PMC3486758 22951995

[48] Baumer, Y., Drenckhahn, D. & Waschke, J. cAMP induced Rac 1-mediated cytoskeletal reorganization in microvascular endothelium. *Histochem. Cell. Biol.* **129**, 765–778. 10.1007/s00418-008-0422-y (2008). 18392843 10.1007/s00418-008-0422-y

[49] Laemmli, U. K. Cleavage of structural proteins during the assembly of the head of bacteriophage T4. *Nature* **227**, 680–685 (1970). 5432063 10.1038/227680a0

[50] Garcia-Ponce, A. et al. Epac1 is crucial for maintenance of endothelial barrier function through A mechanism partly independent of Rac1. *Cells* **9**10.3390/cells9102170 (2020). 10.3390/cells9102170 PMC7601253 32992982

[51] Moztarzadeh, S. et al. Cortactin is in a complex with VE-cadherin and is required for endothelial adherens junction stability through Rap1/Rac1 activation. *Sci. Rep.* **14**, 1218. 10.1038/s41598-024-51269-3 (2024). 38216638 10.1038/s41598-024-51269-3 PMC10786853

[52] Wylie, L. A., Mouillesseaux, K. P., Chong, D. C. & Bautch, V. L. Developmental SMAD6 loss leads to blood vessel hemorrhage and disrupted endothelial cell junctions. *Dev. Biol.* **442**, 199–209. 10.1016/j.ydbio.2018.07.027 (2018). 30098998 10.1016/j.ydbio.2018.07.027 PMC6903908

[53] Eddleston, J., Murdoch, J. N., Copp, A. J. & Stanier, P. Physical and transcriptional map of a 3-Mb region of mouse chromosome 1 containing the gene for the neural tube defect mutant loop-tail (Lp). *Genomics* **56**, 149–159. 10.1006/geno.1998.5701 (1999). 10051400 10.1006/geno.1998.5701

[54] Cong, X. & Kong, W. Endothelial tight junctions and their regulatory signaling pathways in vascular homeostasis and disease. *Cell. Signal.* **66**, 109485. 10.1016/j.cellsig.2019.109485 (2020). 31770579 10.1016/j.cellsig.2019.109485

[55] Schlegel, N. & Waschke, J. Vasodilator-stimulated phosphoprotein: crucial for activation of Rac1 in endothelial barrier maintenance. *Cardiovasc. Res.* **87**, 1–3. 10.1093/cvr/cvq093 (2010). 20308204 10.1093/cvr/cvq093

[56] Moztarzadeh, S. et al. Lack of adducin impairs the stability of endothelial adherens and tight junctions and May be required for cAMP-Rac1-mediated endothelial barrier stabilization. *Sci. Rep.* **12**, 14940. 10.1038/s41598-022-18964-5 (2022). 36056066 10.1038/s41598-022-18964-5 PMC9440001

[57] Schinner, C. et al. Adrenergic signaling strengthens cardiac myocyte cohesion. *Circ. Res.* **120**, 1305–1317. 10.1161/CIRCRESAHA.116.309631 (2017). 28289018 10.1161/CIRCRESAHA.116.309631

[58] Bierkamp, C., McLaughlin, K. J., Schwarz, H., Huber, O. & Kemler, R. Embryonic heart and skin defects in mice lacking Plakoglobin. *Dev. Biol.* **180**, 780–785. 10.1006/dbio.1996.0346 (1996). 8954745 10.1006/dbio.1996.0346

[59] Li, D. et al. Restrictive loss of Plakoglobin in cardiomyocytes leads to arrhythmogenic cardiomyopathy. *Hum. Mol. Genet.* **20**, 4582–4596. 10.1093/hmg/ddr392 (2011). 21880664 10.1093/hmg/ddr392 PMC3209829

[60] Tornavaca, O. et al. ZO-1 controls endothelial adherens junctions, cell-cell tension, angiogenesis, and barrier formation. *J. Cell. Biol.* **208**, 821–838. 10.1083/jcb.201404140 (2015). 25753039 10.1083/jcb.201404140 PMC4362456

[61] Kakogiannos, N. et al. JAM-A acts via C/EBP-alpha to promote Claudin-5 expression and enhance endothelial barrier function. *Circ. Res.* **127**, 1056–1073. 10.1161/CIRCRESAHA.120.316742 (2020). 32673519 10.1161/CIRCRESAHA.120.316742 PMC7508279

[62] Nourshargh, S., Krombach, F. & Dejana, E. The role of JAM-A and PECAM-1 in modulating leukocyte infiltration in inflamed and ischemic tissues. *J. Leukoc. Biol.* **80**, 714–718. 10.1189/jlb.1105645 (2006). 16857733 10.1189/jlb.1105645

[63] Privratsky, J. R. & Newman, P. J. PECAM-1: regulator of endothelial junctional integrity. *Cell. Tissue Res.* **355**, 607–619. 10.1007/s00441-013-1779-3 (2014). 24435645 10.1007/s00441-013-1779-3 PMC3975704

[64] Privratsky, J. R. et al. Relative contribution of PECAM-1 adhesion and signaling to the maintenance of vascular integrity. *J. Cell. Sci.* **124**, 1477–1485. 10.1242/jcs.082271 (2011). 21486942 10.1242/jcs.082271 PMC3078814

[65] Taddei, A. et al. Endothelial adherens junctions control tight junctions by VE-cadherin-mediated upregulation of claudin-5. *Nat. Cell. Biol.* **10**, 923–934. 10.1038/ncb1752 (2008). 18604199 10.1038/ncb1752

[66] Biswas, P. et al. PECAM-1 affects GSK-3beta-mediated beta-catenin phosphorylation and degradation. *Am. J. Pathol.* **169**, 314–324. 10.2353/ajpath.2006.051112 (2006). 16816383 10.2353/ajpath.2006.051112 PMC1698776

[67] Park, S., DiMaio, T. A., Scheef, E. A., Sorenson, C. M. & Sheibani, N. PECAM-1 regulates proangiogenic properties of endothelial cells through modulation of cell-cell and cell-matrix interactions. *Am. J. Physiol. Cell. Physiol.* **299**, C1468–1484. 10.1152/ajpcell.00246.2010 (2010). 20810911 10.1152/ajpcell.00246.2010 PMC3006334

[68] Morita, K. et al. Expression of claudin-5 in dermal vascular endothelia. *Exp. Dermatol.* **12**, 289–295 (2003). 12823443 10.1034/j.1600-0625.2003.120309.x

[69] Morita, K., Sasaki, H., Furuse, M. & Tsukita, S. Endothelial Claudin: claudin-5/TMVCF constitutes tight junction strands in endothelial cells. *J. Cell. Biol.* **147**, 185–194 (1999). 10508865 10.1083/jcb.147.1.185 PMC2164984

[70] Nitta, T. et al. Size-selective loosening of the blood-brain barrier in claudin-5-deficient mice. *J. Cell. Biol.* **161**, 653–660. 10.1083/jcb.200302070 (2003). 12743111 10.1083/jcb.200302070 PMC2172943

[71] Asaka, M., Hirase, T., Hashimoto-Komatsu, A. & Node, K. Rab5a-mediated localization of claudin-1 is regulated by proteasomes in endothelial cells. *Am. J. Physiol. Cell. Physiol.* **300**, C87–96. 10.1152/ajpcell.00565.2010 (2011). 20926780 10.1152/ajpcell.00565.2010

[72] Liebner, S. et al. Claudin-1 and claudin-5 expression and tight junction morphology are altered in blood vessels of human glioblastoma multiforme. *Acta Neuropathol.* **100**, 323–331. 10.1007/s004010000180 (2000). 10965803 10.1007/s004010000180

[73] Sladojevic, N. et al. Claudin-1-Dependent destabilization of the Blood-Brain barrier in chronic stroke. *J. Neurosci.* **39**, 743–757. 10.1523/JNEUROSCI.1432-18.2018 (2019). 30504279 10.1523/JNEUROSCI.1432-18.2018 PMC6343646

[74] Berndt, P. et al. Tight junction proteins at the blood-brain barrier: Far more than claudin-5. *Cell. Mol. Life Sci.* **76**, 1987–2002. 10.1007/s00018-019-03030-7 (2019). 30734065 10.1007/s00018-019-03030-7 PMC11105330

[75] Hippenstiel, S. et al. Adrenomedullin reduces endothelial hyperpermeability. *Circ. Res.* **91**, 618–625. 10.1161/01.res.0000036603.61868.f9 (2002). 12364390 10.1161/01.res.0000036603.61868.f9

[76] Garcia Ponce, A. et al. Loss of cortactin causes endothelial barrier dysfunction via disturbed adrenomedullin secretion and actomyosin contractility. *Sci. Rep.* **6**, 29003. 10.1038/srep29003 (2016). 27357373 10.1038/srep29003 PMC4928053

[77] Weed, S. A., Du, Y. & Parsons, J. T. Translocation of cortactin to the cell periphery is mediated by the small GTPase Rac1. *J. Cell. Sci.* **111** (Pt 16), 2433–2443 (1998). 9683637 10.1242/jcs.111.16.2433

[78] Schlegel, N. et al. The role of VASP in regulation of cAMP- and Rac 1-mediated endothelial barrier stabilization. *Am. J. Physiol. Cell. Physiol.* **294**, C178–188. 10.1152/ajpcell.00273.2007 (2008). 17989211 10.1152/ajpcell.00273.2007

[79] Sassone-Corsi, P. The Cyclic AMP pathway. *Cold Spring Harb Perspect. Biol.* **4**10.1101/cshperspect.a011148 (2012). 10.1101/cshperspect.a011148 PMC3504441 23209152

[80] Kritzer, M. D., Li, J., Dodge-Kafka, K. & Kapiloff, M. S. AKAPs: the architectural underpinnings of local cAMP signaling. *J. Mol. Cell. Cardiol.* **52**, 351–358. 10.1016/j.yjmcc.2011.05.002 (2012). 21600214 10.1016/j.yjmcc.2011.05.002 PMC3168680

[81] Radeva, M. Y., Kugelmann, D., Spindler, V. & Waschke, J. PKA compartmentalization via AKAP220 and AKAP12 contributes to endothelial barrier regulation. *PLoS One*. **9**, e106733. 10.1371/journal.pone.0106733 (2014). 25188285 10.1371/journal.pone.0106733 PMC4154725

[82] Spindler, V., Schlegel, N. & Waschke, J. Role of GTPases in control of microvascular permeability. *Cardiovasc. Res.* **87**, 243–253. 10.1093/cvr/cvq086 (2010). 20299335 10.1093/cvr/cvq086

[83] Wu, X. et al. Rac1 activation controls nuclear localization of beta-catenin during canonical Wnt signaling. *Cell* **133**, 340–353. 10.1016/j.cell.2008.01.052 (2008). 18423204 10.1016/j.cell.2008.01.052 PMC2390926

[84] Esufali, S. & Bapat, B. Cross-talk between Rac1 GTPase and dysregulated Wnt signaling pathway leads to cellular redistribution of beta-catenin and TCF/LEF-mediated transcriptional activation. *Oncogene* **23**, 8260–8271. 10.1038/sj.onc.1208007 (2004). 15377999 10.1038/sj.onc.1208007

[85] Rossol-Allison, J. et al. Rho GTPase activity modulates Wnt3a/beta-catenin signaling. *Cell. Signal.* **21**, 1559–1568. 10.1016/j.cellsig.2009.05.010 (2009). 19482078 10.1016/j.cellsig.2009.05.010 PMC2735600

[86] Dejana, E., Orsenigo, F. & Lampugnani, M. G. The role of adherens junctions and VE-cadherin in the control of vascular permeability. *J. Cell. Sci.* **121**, 2115–2122. 10.1242/jcs.017897 (2008). 18565824 10.1242/jcs.017897

[87] Jin, Y. et al. Tyrosine-protein kinase yes controls endothelial junctional plasticity and barrier integrity by regulating VE-cadherin phosphorylation and endocytosis. *Nat. Cardiovasc. Res.* **1**, 1156–1173. 10.1038/s44161-022-00172-z (2022). 37936984 10.1038/s44161-022-00172-z PMC7615285

[88] Vestweber, D. Vascular endothelial protein tyrosine phosphatase regulates endothelial function. *Physiol. (Bethesda)*. **36**, 84–93. 10.1152/physiol.00026.2020 (2021). 10.1152/physiol.00026.2020 33595386

[89] Esser, S., Lampugnani, M. G., Corada, M., Dejana, E. & Risau, W. Vascular endothelial growth factor induces VE-cadherin tyrosine phosphorylation in endothelial cells. *J. Cell. Sci.* **111** (Pt 13), 1853–1865. 10.1242/jcs.111.13.1853 (1998). 9625748 10.1242/jcs.111.13.1853

[90] Lampugnani, M. G. et al. Cell confluence regulates tyrosine phosphorylation of adherens junction components in endothelial cells. *J. Cell. Sci.* **110** (Pt 17), 2065–2077. 10.1242/jcs.110.17.2065 (1997). 9378757 10.1242/jcs.110.17.2065

[91] McLachlan, R. W. & Yap, A. S. Not so simple: the complexity of phosphotyrosine signaling at Cadherin adhesive contacts. *J. Mol. Med. (Berl)*. **85**, 545–554. 10.1007/s00109-007-0198-x (2007). 17429596 10.1007/s00109-007-0198-x

[92] Juettner, V. V. et al. VE-PTP stabilizes VE-cadherin junctions and the endothelial barrier via a phosphatase-independent mechanism. *J. Cell. Biol.* **218**, 1725–1742. 10.1083/jcb.201807210 (2019). 30948425 10.1083/jcb.201807210 PMC6504901

[93] Broermann, A. et al. Dissociation of VE-PTP from VE-cadherin is required for leukocyte extravasation and for VEGF-induced vascular permeability in vivo. *J. Exp. Med.* **208**, 2393–2401. 10.1084/jem.20110525 (2011). 22025303 10.1084/jem.20110525 PMC3256962

[94] Hatanaka, K., Simons, M. & Murakami, M. Phosphorylation of VE-cadherin controls endothelial phenotypes via p120-catenin coupling and Rac1 activation. *Am. J. Physiol. Heart Circ. Physiol.* **300**, H162–172. 10.1152/ajpheart.00650.2010 (2011). 21037229 10.1152/ajpheart.00650.2010 PMC3023264

[95] Potter, M. D., Barbero, S. & Cheresh, D. A. Tyrosine phosphorylation of VE-cadherin prevents binding of p120- and beta-catenin and maintains the cellular mesenchymal state. *J. Biol. Chem.* **280**, 31906–31912. 10.1074/jbc.M505568200 (2005). 16027153 10.1074/jbc.M505568200

[96] Kooistra, M. R., Corada, M., Dejana, E. & Bos, J. L. Epac1 regulates integrity of endothelial cell junctions through VE-cadherin. *FEBS Lett.* **579**, 4966–4972. 10.1016/j.febslet.2005.07.080 (2005). 16115630 10.1016/j.febslet.2005.07.080

