# Supplementary figures and images for: Plakoglobin does not participate in endothelial barrier stabilization mediated by cAMP
Source: Sci Rep. 2025 Mar 16;15:9043. doi: 10.1038/s41598-025-93756-1 (PMC11911453; doi:10.1038/s41598-025-93756-1)

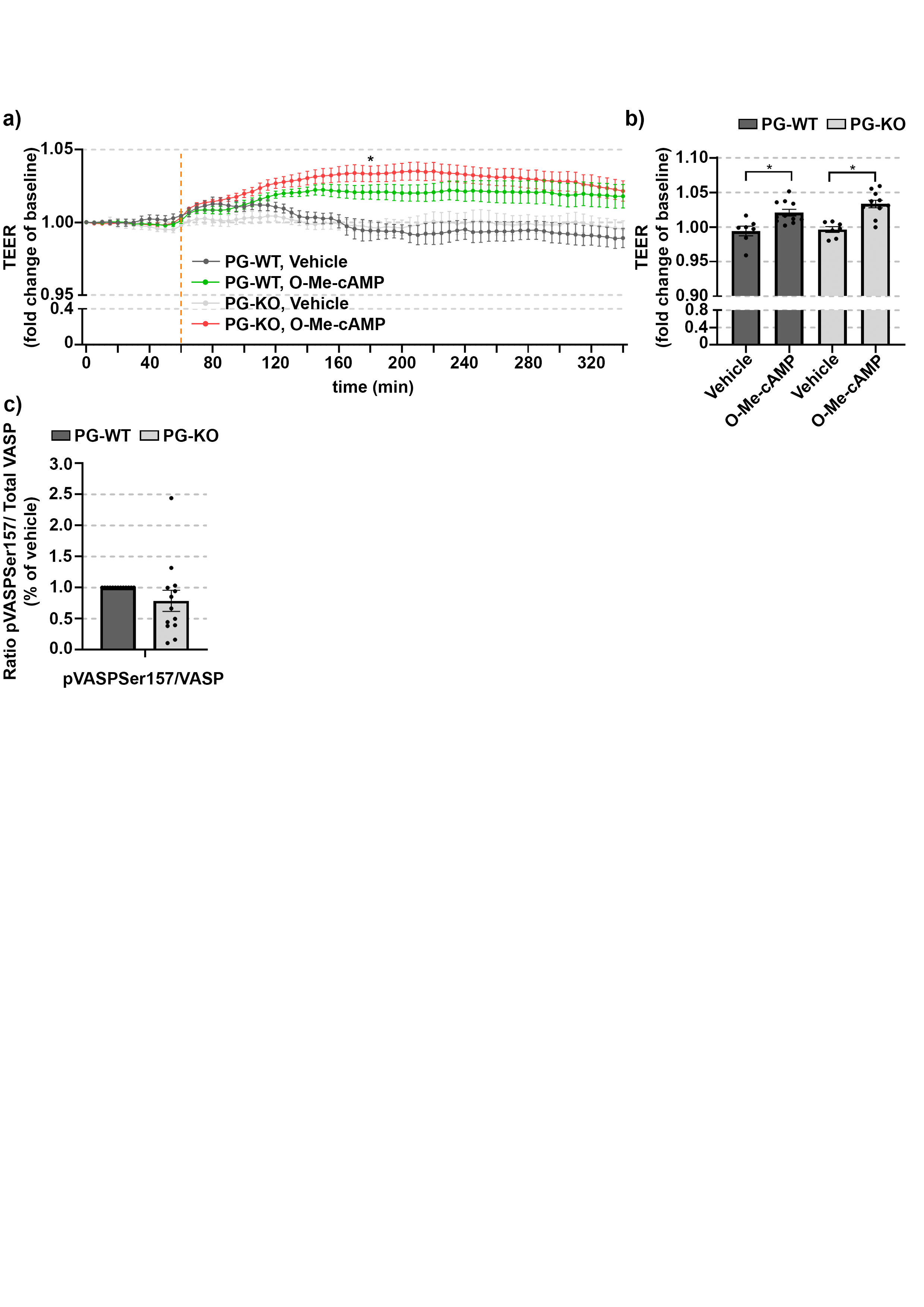

Supplement: Supplementary file 1 — Supplementary Material 1 [file 41598_2025_93756_MOESM1_ESM.tif]

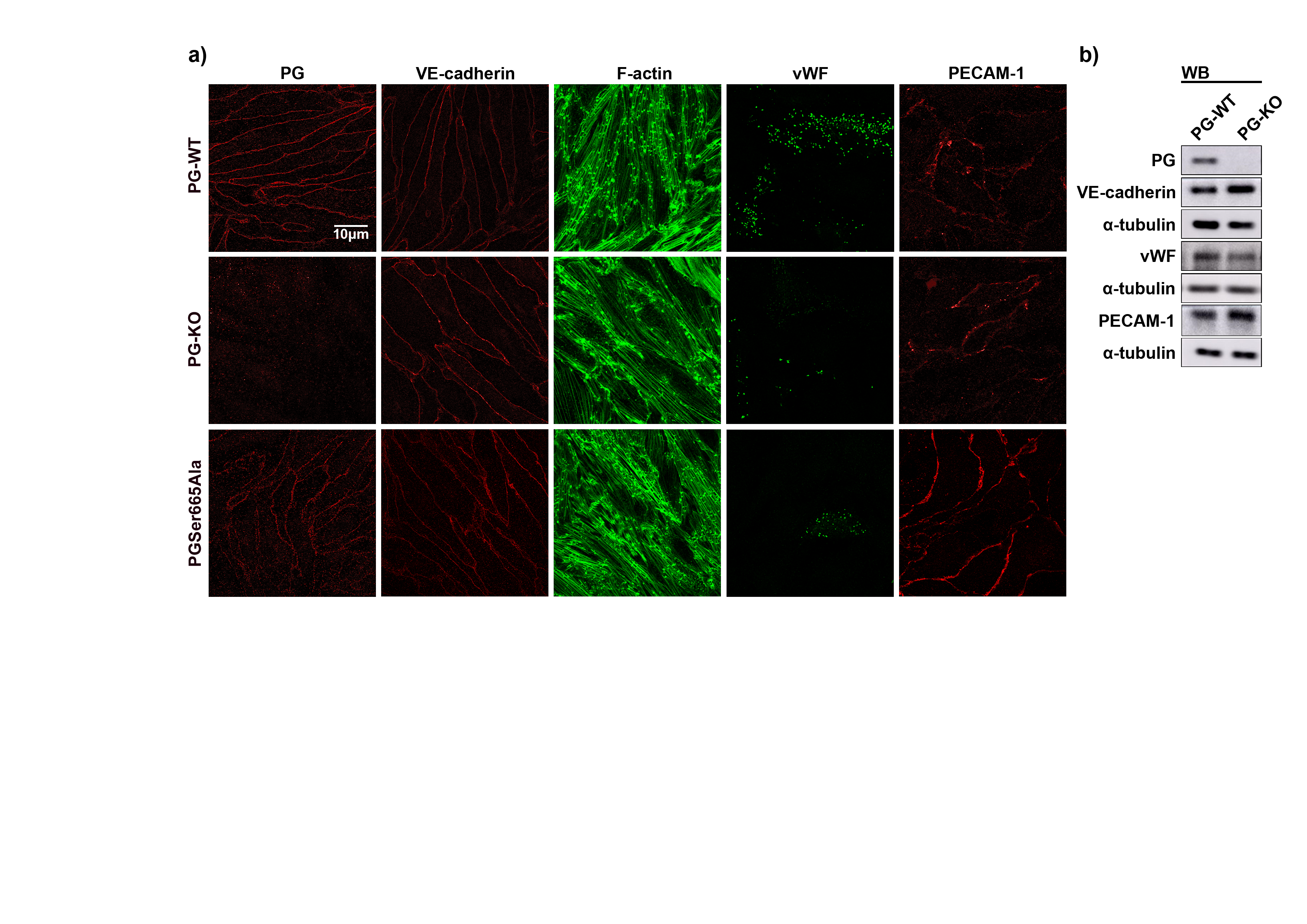

Supplement: Supplementary file 2 — Supplementary Material 2 [file 41598_2025_93756_MOESM2_ESM.tif]

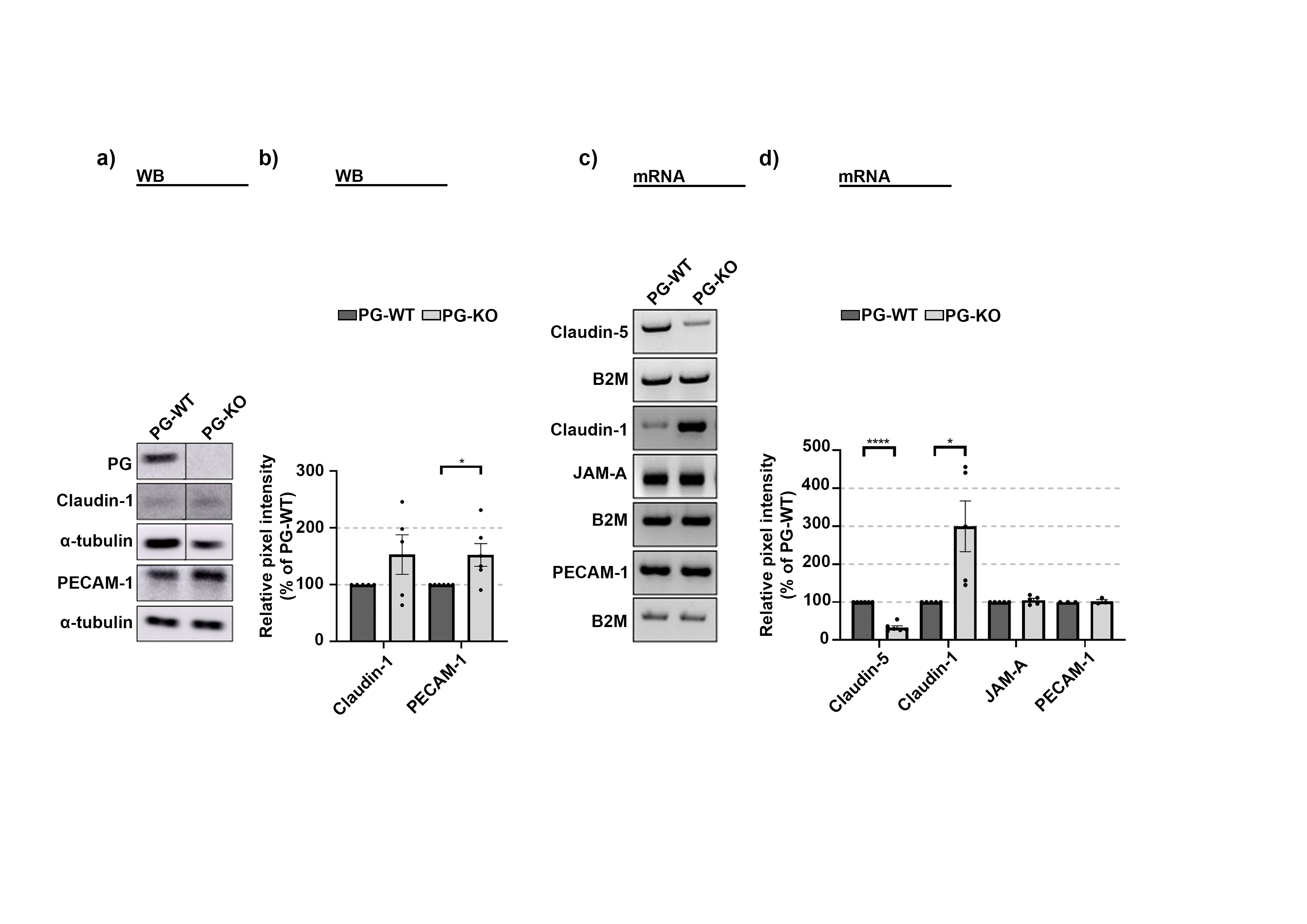

Supplement: Supplementary file 3 — Supplementary Material 3 [file 41598_2025_93756_MOESM3_ESM.tif]
