# Supplementary material for: Plakoglobin does not participate in endothelial barrier stabilization mediated by cAMP
Source: Sci Rep. 2025 Mar 16;15:9043. doi: 10.1038/s41598-025-93756-1 (PMC11911453; doi:10.1038/s41598-025-93756-1)
Supplement: Supplementary file 4 — Supplementary Material 4 [file 41598_2025_93756_MOESM4_ESM.pdf]

cAMP-mediated endothelial barrier stabilization is plakoglobin-independent

Ibrahim Hamad, Sara Sepic, Sina Moztarzadeh, Alexander García-Ponce, Jens Waschke and Mariya Y. Radeva*

Chair of Vegetative Anatomy, Institute of Anatomy, Faculty of Medicine, Ludwig-Maximilians-University (LMU), Pettenkoferstraße 11, 80336, Munich, Germany.

**corresponding author*

Tel. +49 89 2180 - 72701

Mail: mariya.radeva@med.uni-muenchen.de

Figure S1

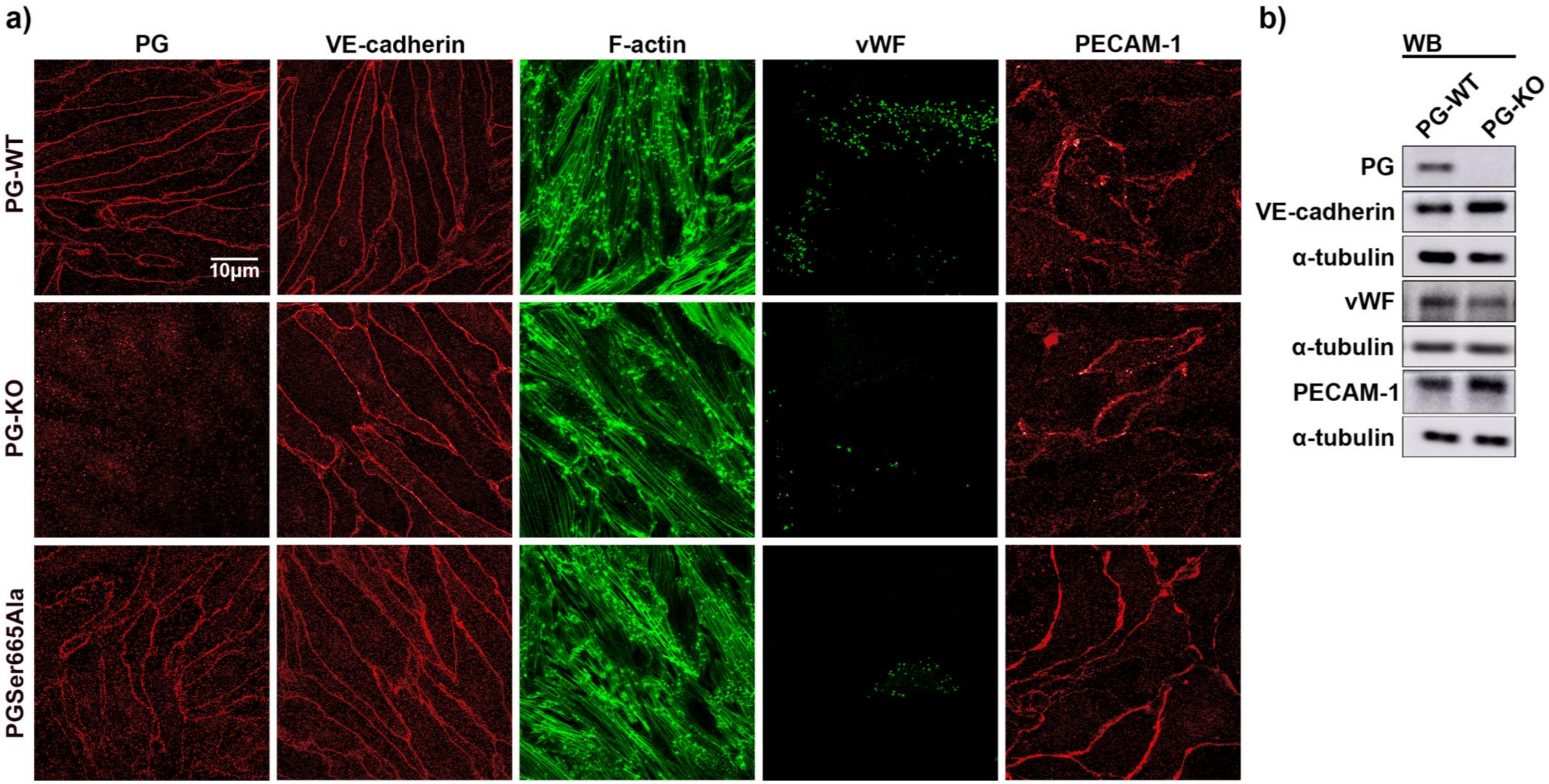

Figure S1

Cells derived from WT and PG-KO mice express typical endothelial markers
a) The presence of endothelial specific markers such as VE-cadherin, vWF and PECAM-1 was validated by immunostaining. Presence or lack of PG was also confirmed (the WB images for PG, VE-cadherin and respective loading control were also used in Figure 2). Actin cytoskeleton organization was visualized with Alexa Fluor®488 Phalloidin; N=3 b) Cellular abundance of the proteins presented in a) was proven by Western blot analyses. Equal loading was ensured by detection of α-tubulin; N≥5

Figure S2

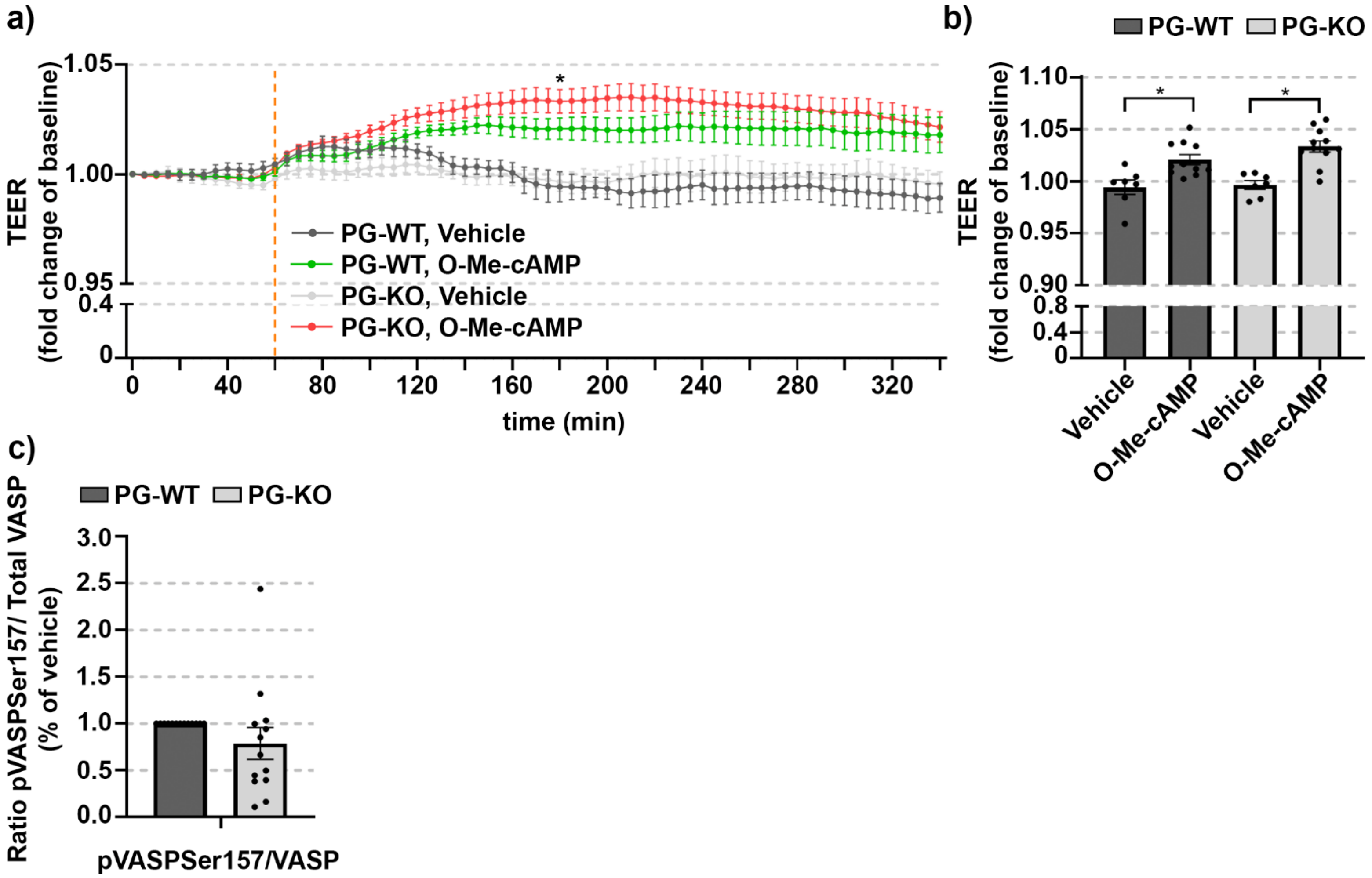

Figure S2

Barrier integrity behaviour in WT and PG-KO cells, exposed to O-Me-cAMP treatment

a) Time course of TEER measurements in WT and PG-depleted confluent monolayers treated with either Vehicle or O-Me-cAMP. The recorded data were normalized to the initial point of measure. The segmented orange line depicts the time of primary treatment; N=5 b) Bar diagram representing the significant differences in the average TEER measurements between Vehicle and O-Me-cAMP treated cells, in WT and PG-null cells, respectively. The analysis is done 2 h after initial application; N=5. c) Bar diagram illustrating the phosphorylation of VASP in WT and PG-depleted MyEnd cells under basal condition; N≥5. Data are presented as mean ± SEM; unpaired t-test; * p≤0,05

Figure S3

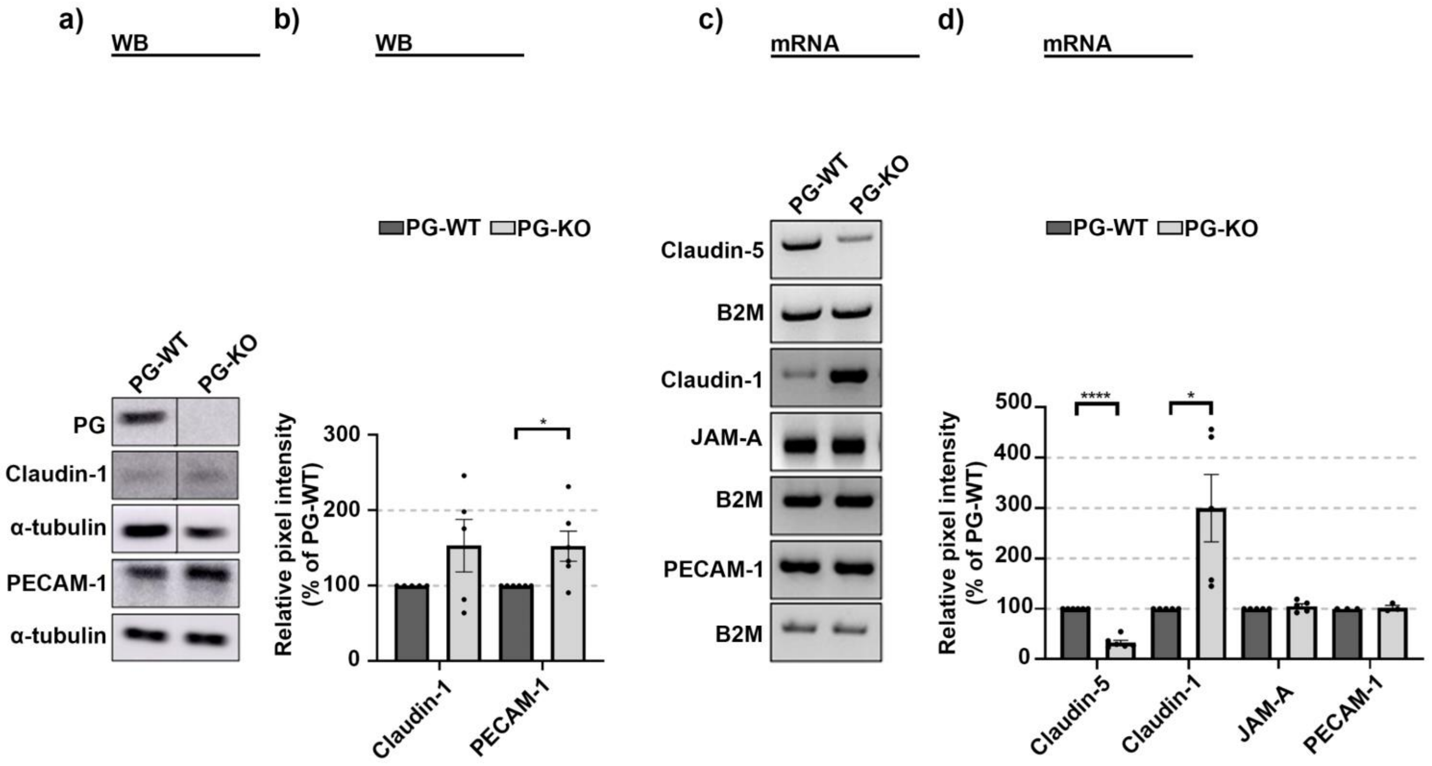

Supplementary Figure 3

Effect of PG deficiency on central junctional molecules

a) Representative Western blot analyses of claudin-1 and PECAM-1 in WT and PG-KO cells, α-tubulin was used as loading control. Non-contiguous bands for PG, claudin-1 and α-tubulin originating from the same membrane are distinct by a vertical black line. An equal exposure time was used for detection of the specific bands. Original blots are presented in Figure S4 a, s-t, provided in the Supplementary info file. The images, representing PECAM-1 protein expression in WT and PG-KO are the same used in Supplementary Figure 1; N≥5 b) Bar diagrams depicting the relative protein expression of each analyzed target of interest in PG-KO when compared to WT cells. c) PCR analysis, revealing the transcription levels of TJ-related claudin-1, -5, JAM-A and PECAM-1 in WT and PG-KO cells. Equal loading for the PCR analyses was validated by B2M expression. Original agarose gels are presented in Figure S5 h-k from the Supplementary info file; N≥3 c) d) Bar diagram presenting the mRNA expression in PG-KO, relative to WT cells. Data are presented as mean ± SEM; the following values were considered significant * p≤0,05; **** p≤0,0001.

Original western blot gel images for basal condition

Figure S4 a)

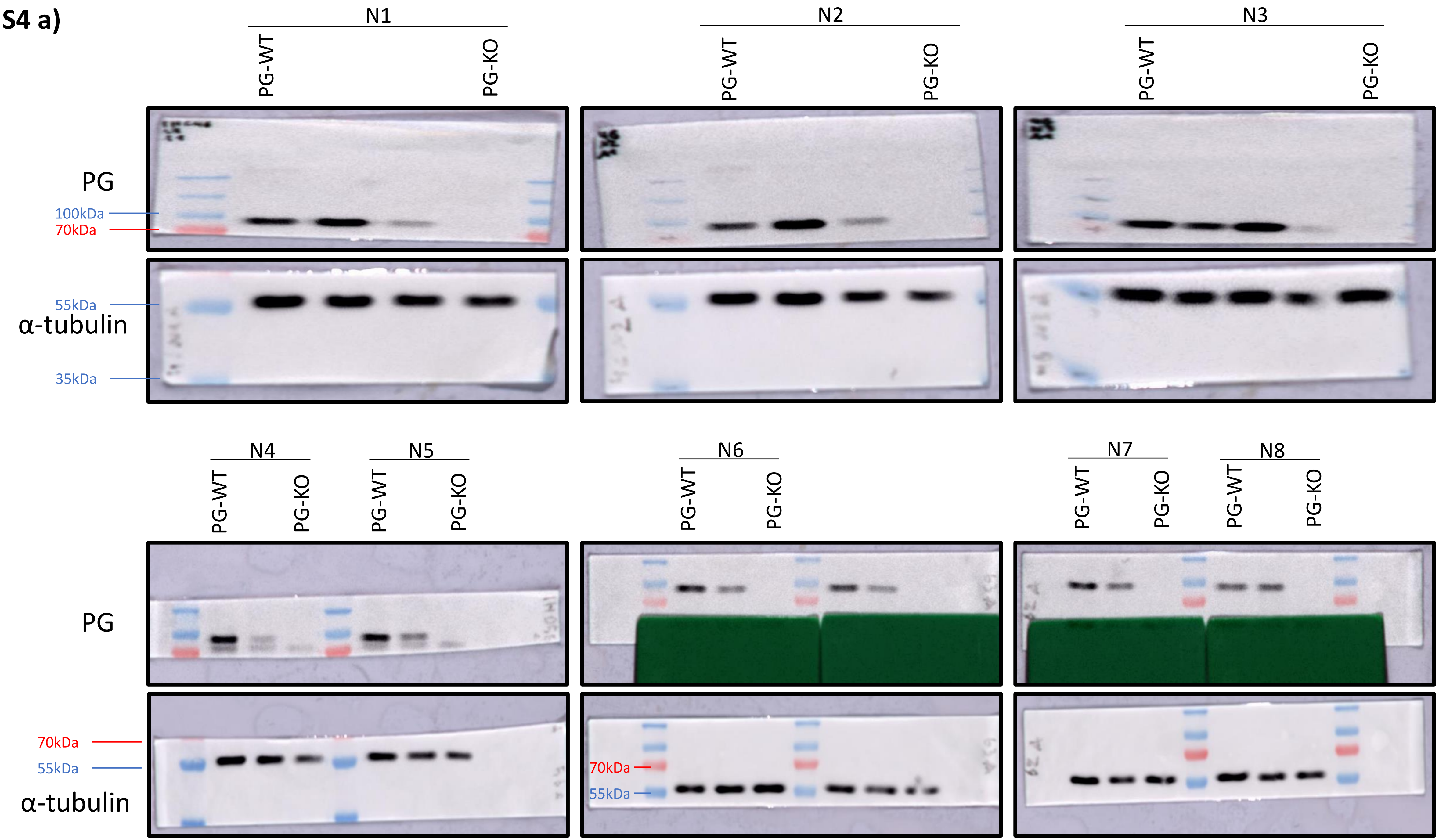
N1
PG-WT
PG-KO
N2
PG-WT
PG-KO
N3
PG-WT
PG-KO
PG
100kDa
70kDa
55kDa
α-tubulin
35kDa
N4
PG-WT
PG-KO
N5
PG-WT
PG-KO
N6
PG-WT
PG-KO
N7
PG-WT
PG-KO
N8
PG-WT
PG-KO
PG
70kDa
55kDa
α-tubulin
70kDa
55kDa

Figure S4 b)

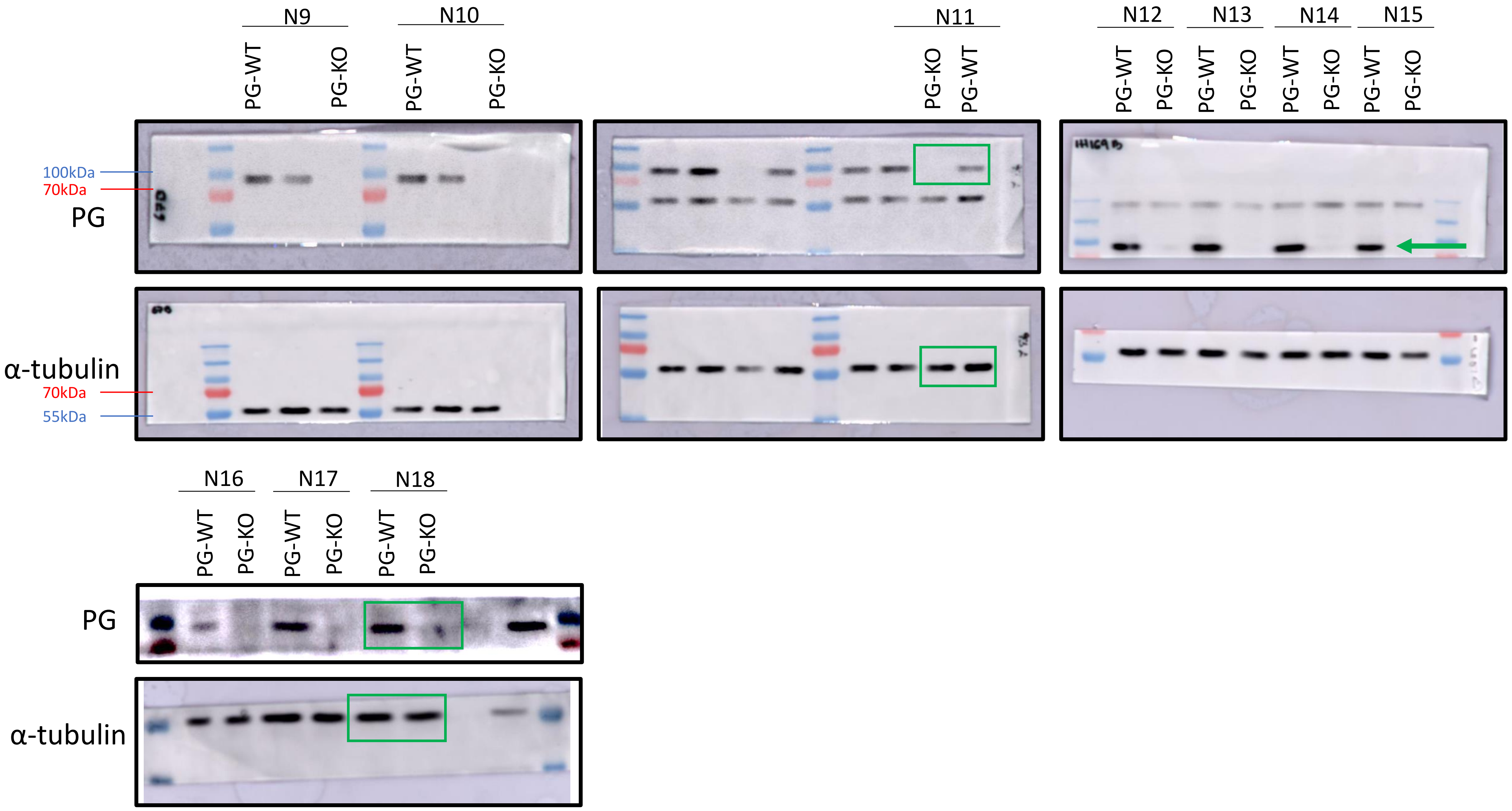
N9
N10
N11
N12
N13
N14
N15
PG-WT
PG-KO
PG-WT
PG-KO
PG-KO
PG-WT
PG-WT
PG-KO
PG-WT
PG-KO
PG-WT
PG-KO
PG-WT
PG-KO
100kDa
70kDa
PG
α-tubulin
70kDa
55kDa
N16
N17
N18
PG-WT
PG-KO
PG-WT
PG-KO
PG-WT
PG-KO
PG
α-tubulin

Figure S4 c)

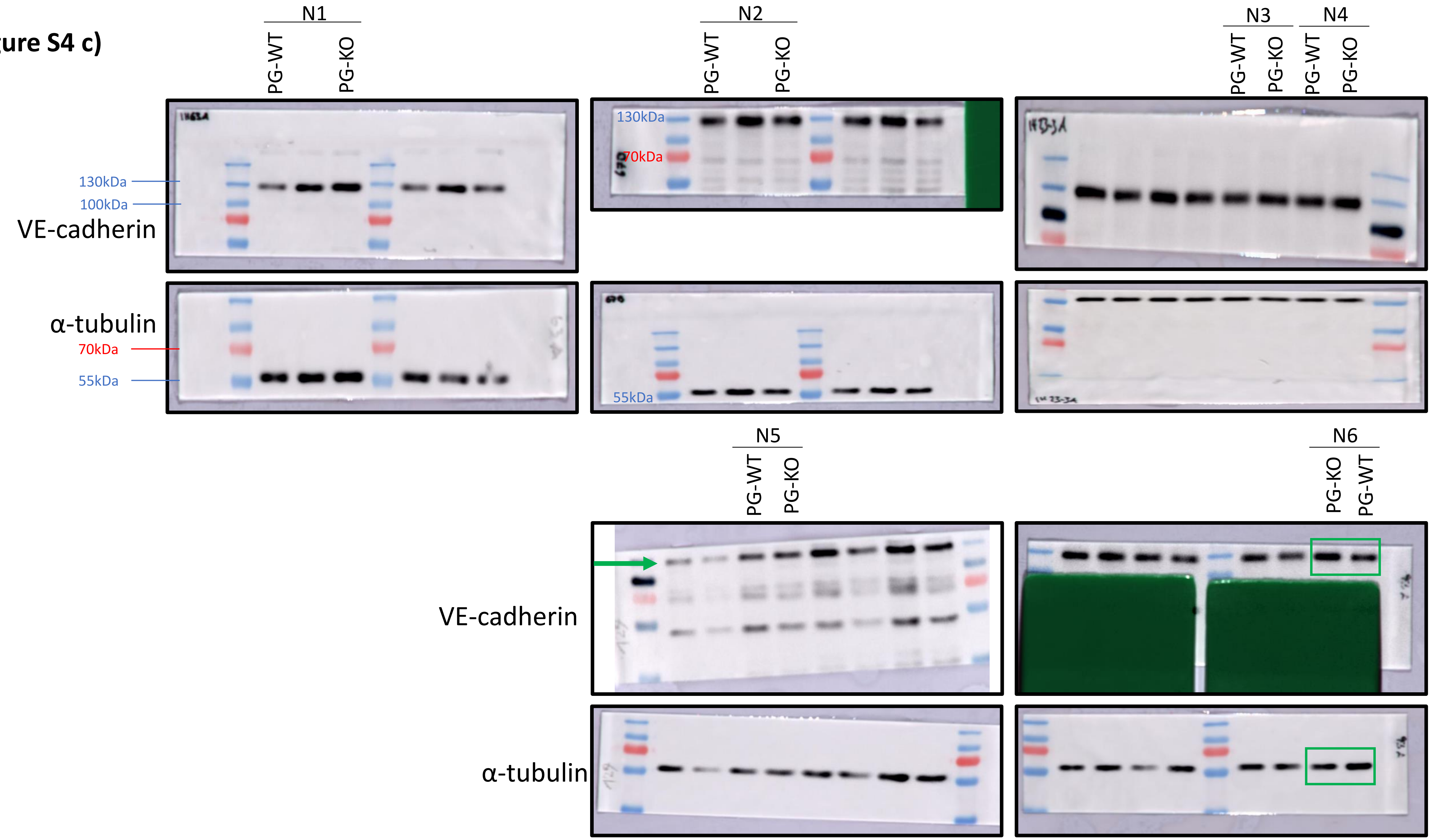
N1
PG-WT
PG-KO
N2
PG-WT
PG-KO
N3
PG-WT
PG-KO
N4
PG-WT
PG-KO
130kDa
100kDa
VE-cadherin
130kDa
70kDa
α-tubulin
70kDa
55kDa
55kDa
N5
PG-WT
PG-KO
N6
PG-KO
PG-WT
VE-cadherin
α-tubulin

Figure S4 d)

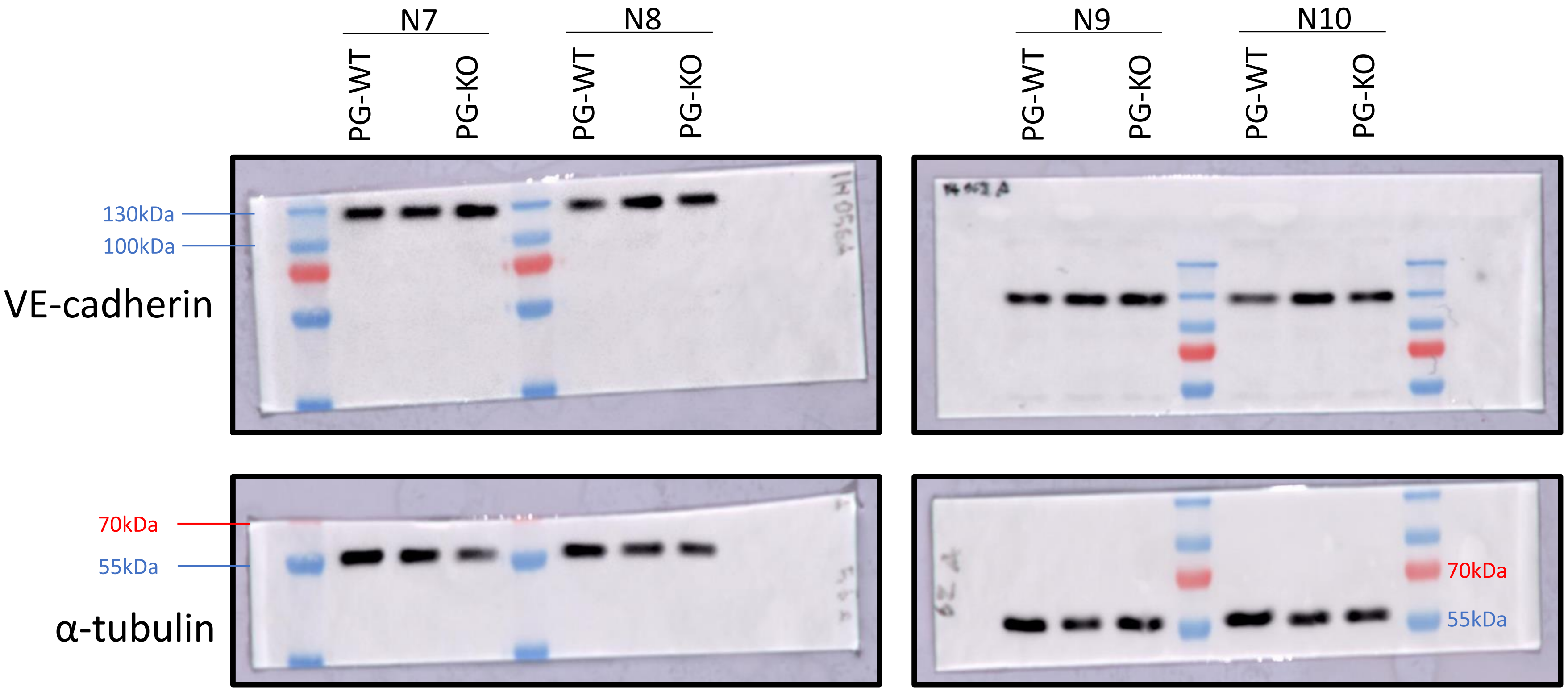

Figure S4, a-d) Original Western Blot gel images for basal condition. To prevent over exposure, the black or green cover was used. The green square determines the selected blot used in the figure 2 and S1.

Figure S4 e)

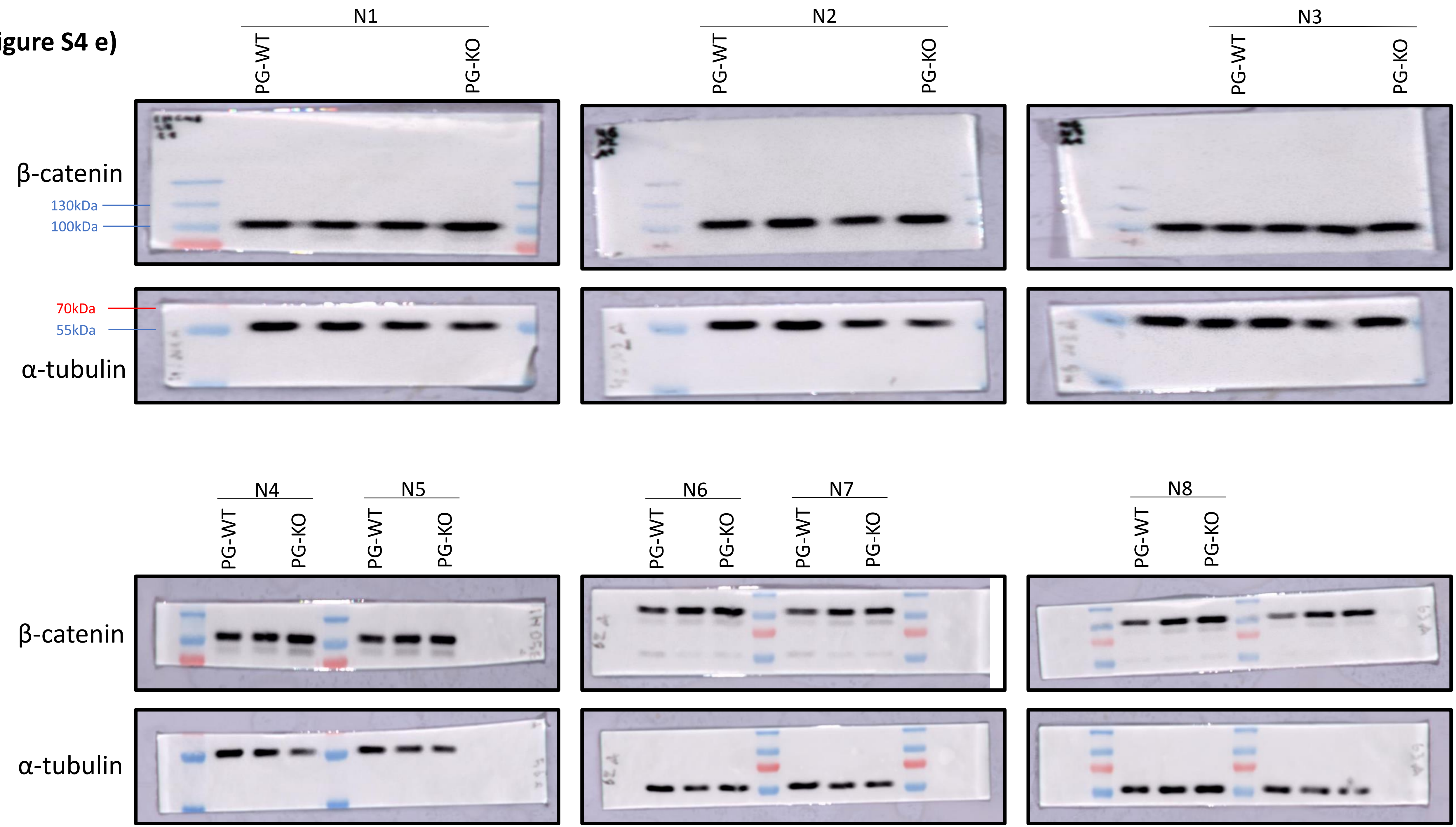
N1
N2
N3
PG-WT
PG-KO
β-catenin
130kDa
100kDa
70kDa
55kDa
α-tubulin
N4
N5
N6
N7
N8

Figure S4 f)

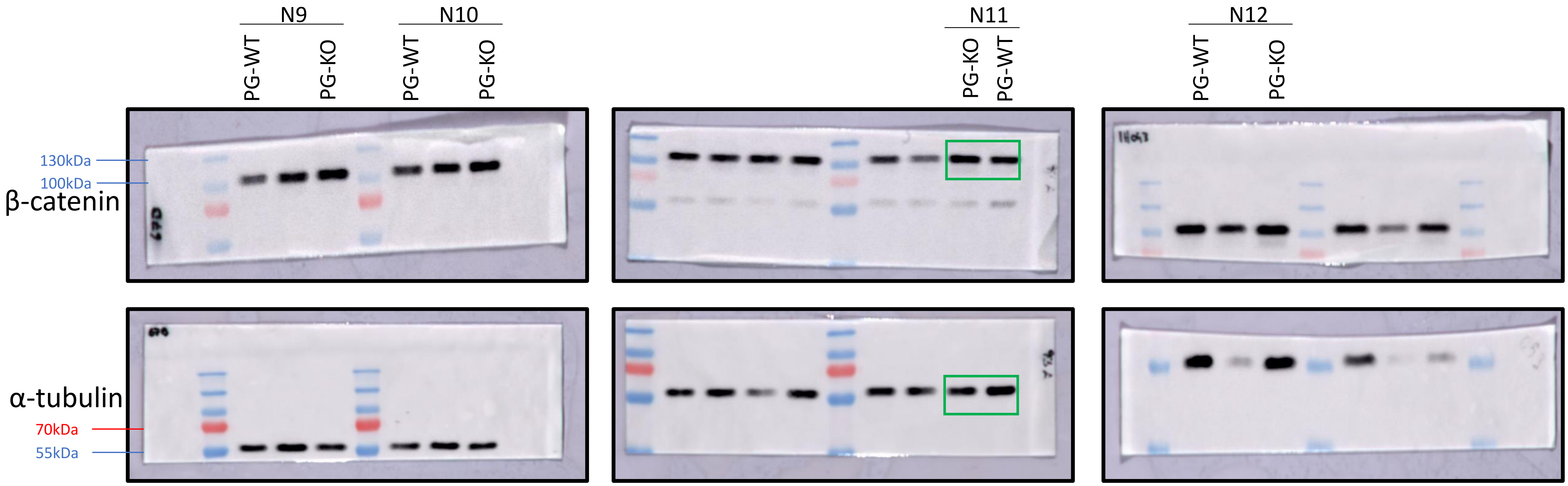
N9
N10
N11
N12
PG-WT
PG-KO
PG-WT
PG-KO
PG-KO
PG-WT
PG-WT
PG-KO
130kDa
100kDa
β-catenin
α-tubulin
70kDa
55kDa

Figure S4 g)

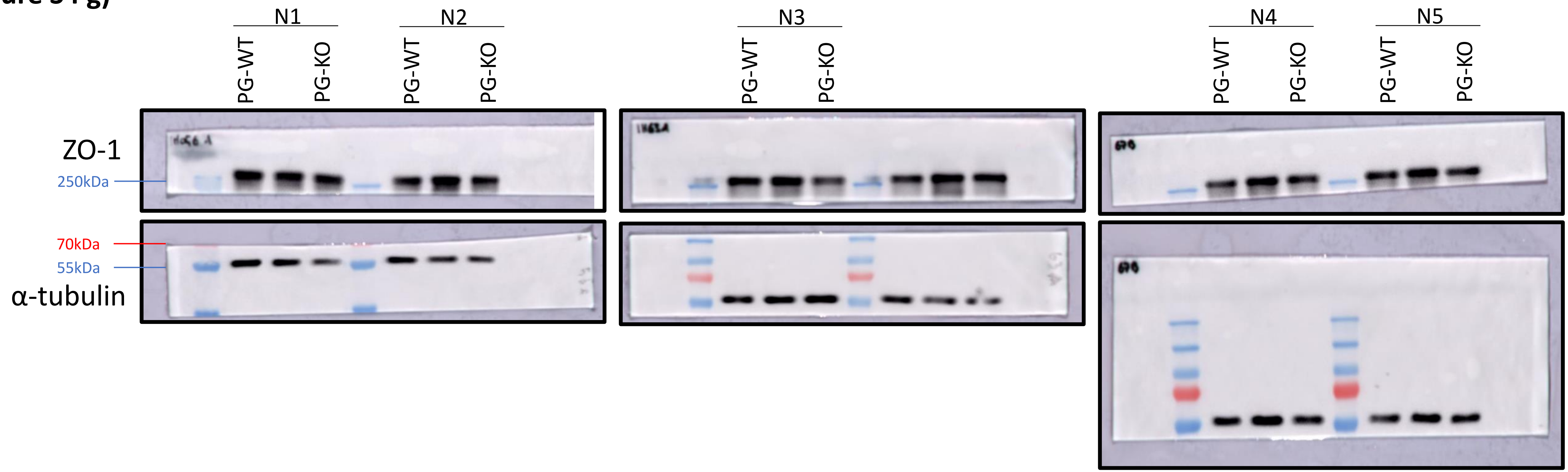
N1
N2
N3
N4
N5
PG-WT
PG-KO
PG-WT
PG-KO
PG-WT
PG-KO
PG-WT
PG-KO
PG-WT
PG-KO
ZO-1
250kDa
70kDa
55kDa
α-tubulin

Figure S4 h)

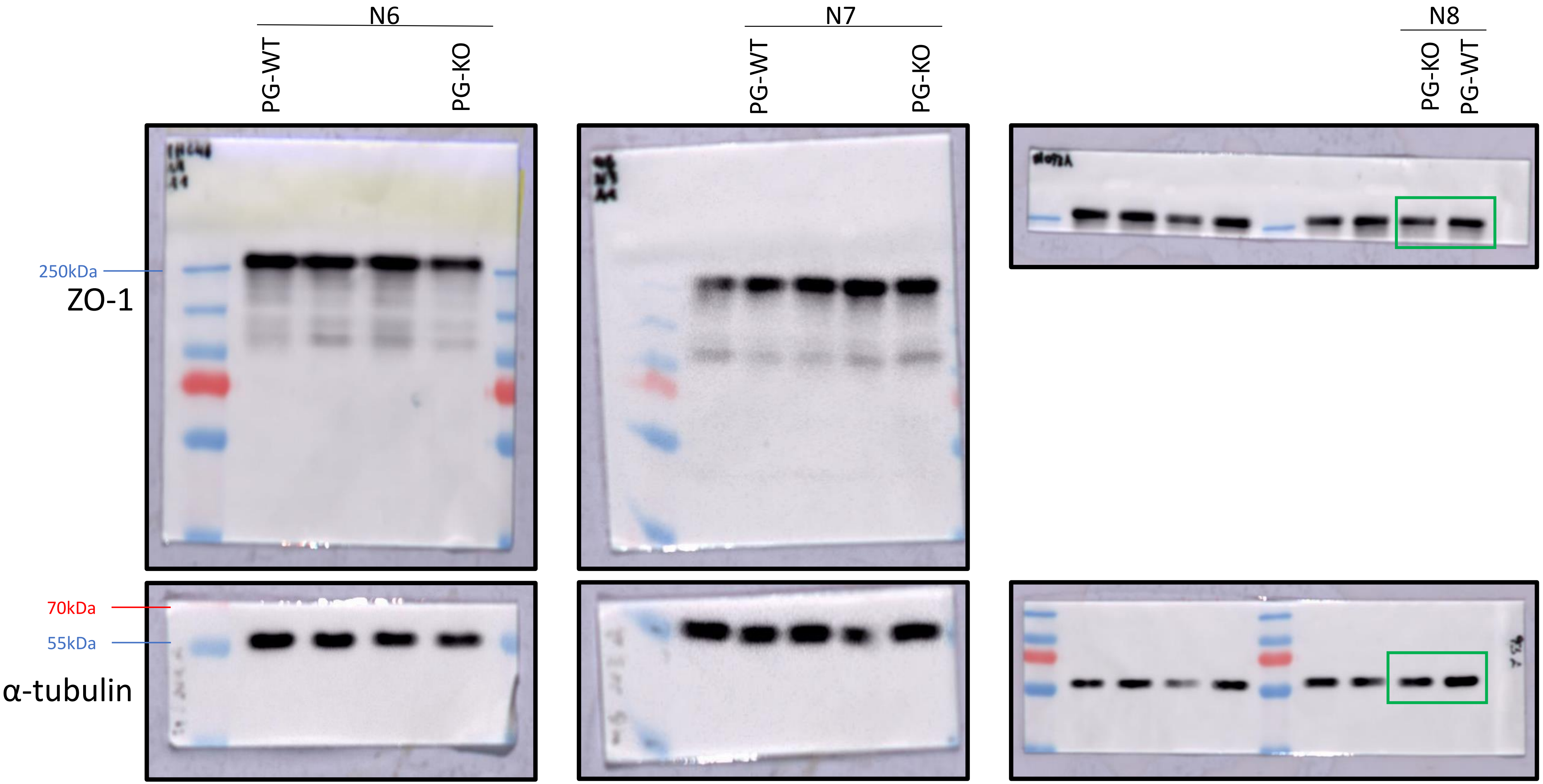
N6
PG-WT
PG-KO
N7
PG-WT
PG-KO
N8
PG-KO
PG-WT
250kDa
ZO-1
70kDa
55kDa
α-tubulin

Figure S4 i)

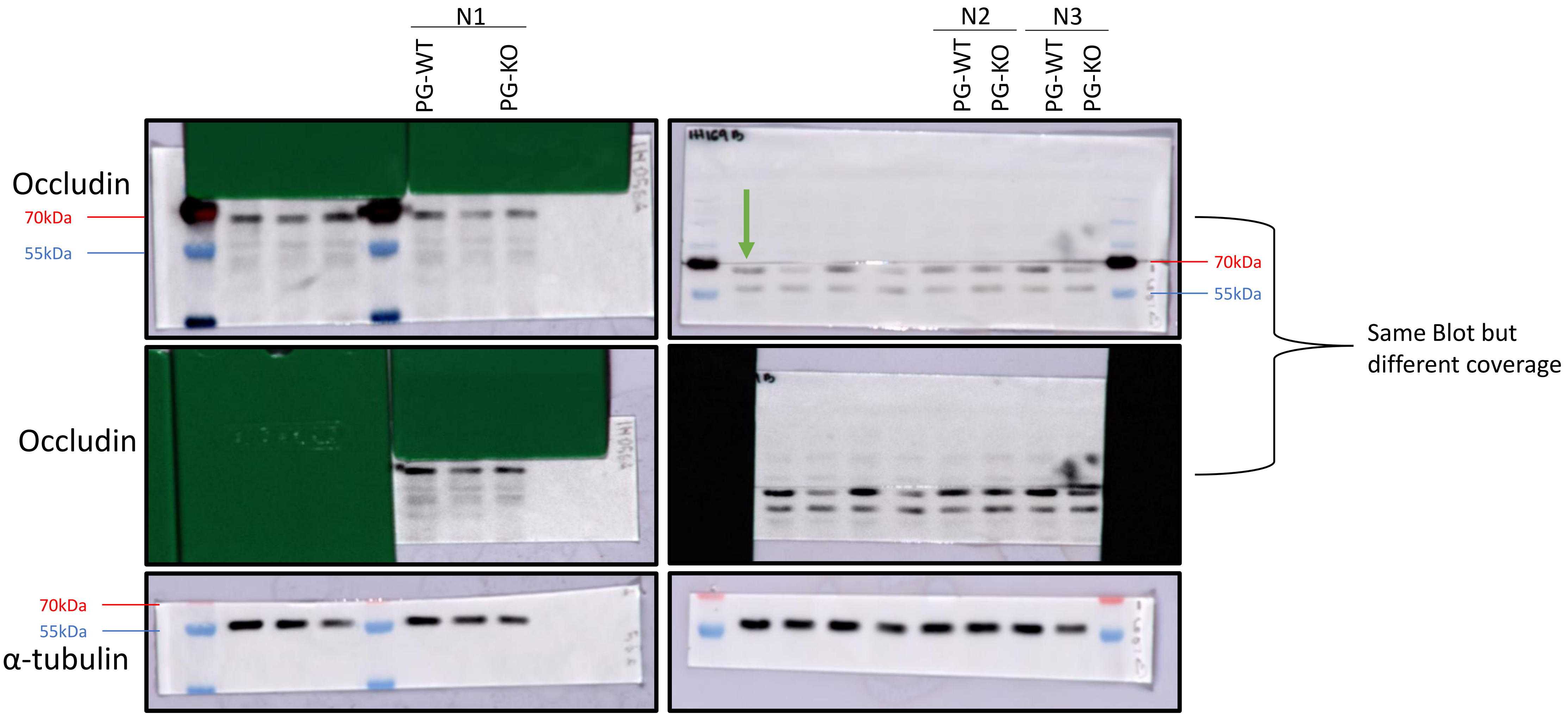
N1
PG-WT
PG-KO
N2
PG-WT
PG-KO
N3
PG-WT
PG-KO
Occludin
70kDa
55kDa
Occludin
70kDa
55kDa
α-tubulin
70kDa
55kDa
Same Blot but different coverage

Figure S4 j)

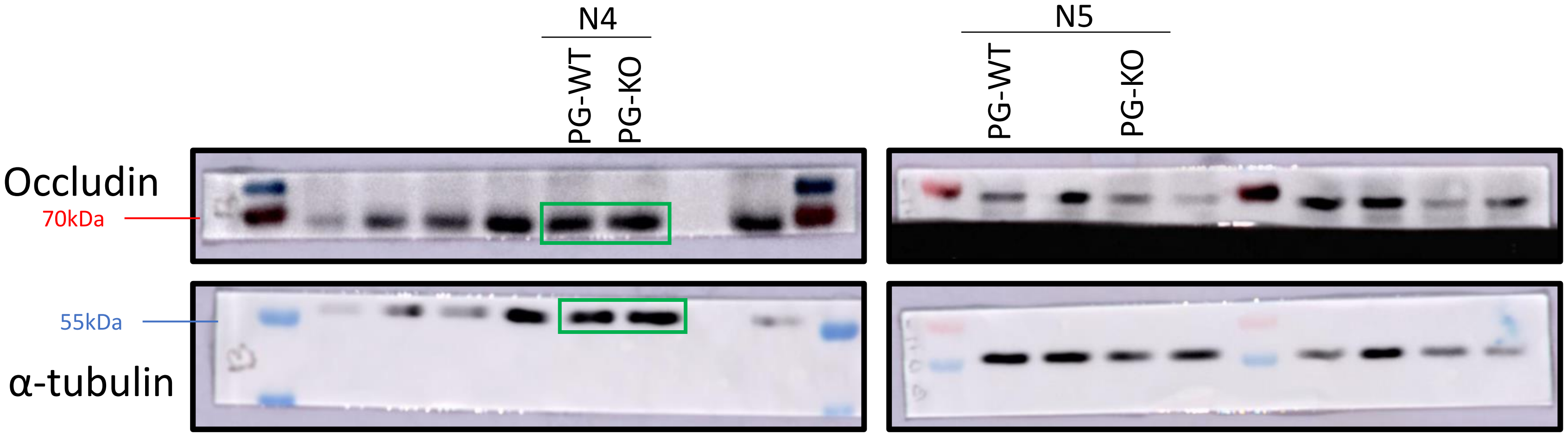

Figure S4 k)

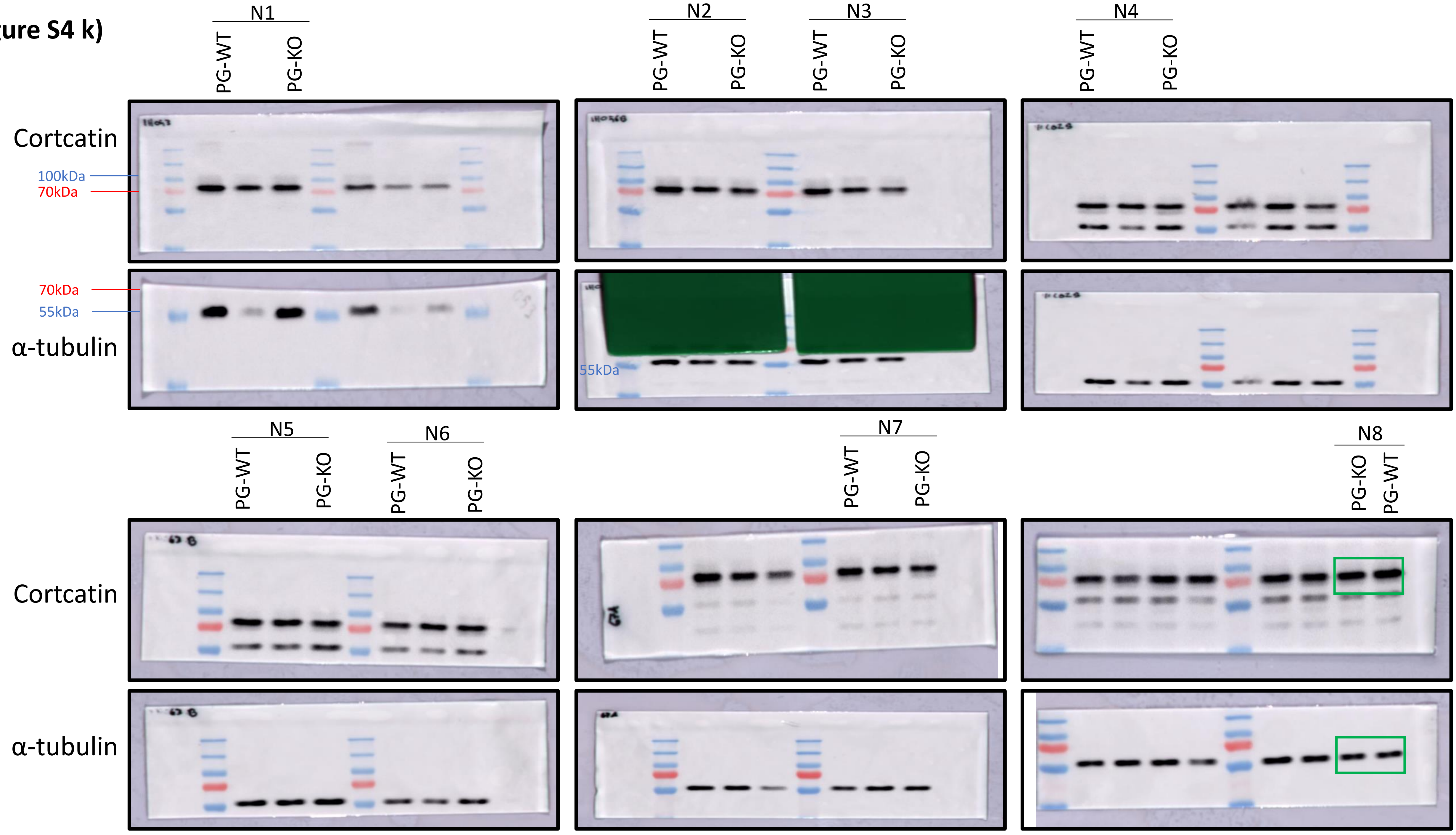
N1
PG-WT
PG-KO
N2
PG-WT
PG-KO
N3
PG-WT
PG-KO
N4
PG-WT
PG-KO
Cortcatin
100kDa
70kDa
70kDa
55kDa
α-tubulin
55kDa
N5
PG-WT
PG-KO
N6
PG-WT
PG-KO
N7
PG-WT
PG-KO
N8
PG-KO
PG-WT
Cortcatin
α-tubulin

Figure S4 l)

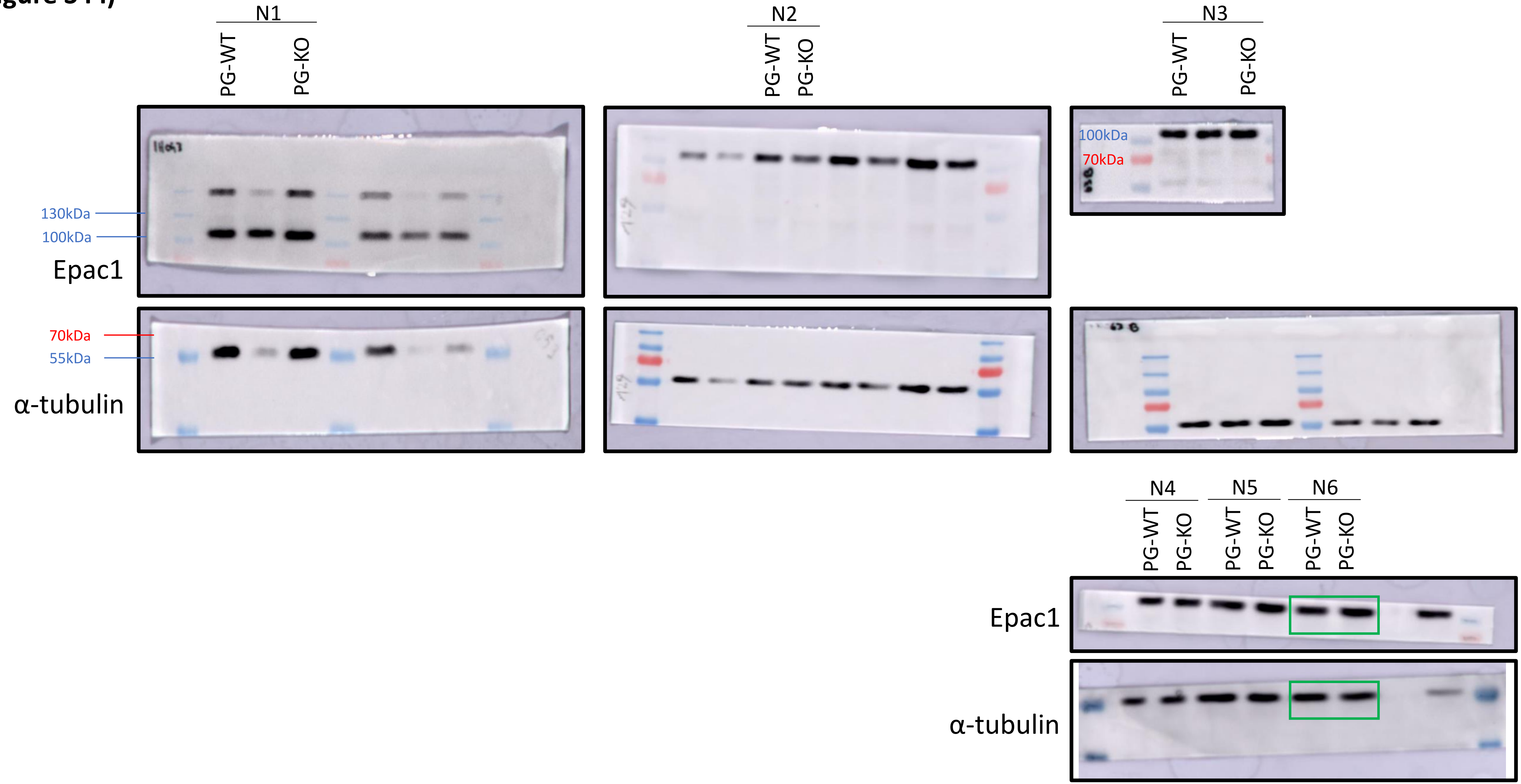

N1
PG-WT
PG-KO
N2
PG-WT
PG-KO
N3
PG-WT
PG-KO
130kDa
100kDa
Epac1
70kDa
55kDa
α-tubulin
100kDa
70kDa
N4
N5
N6
PG-WT
PG-KO
PG-WT
PG-KO
PG-WT
PG-KO
Epac1
α-tubulin

Figure S4 m)

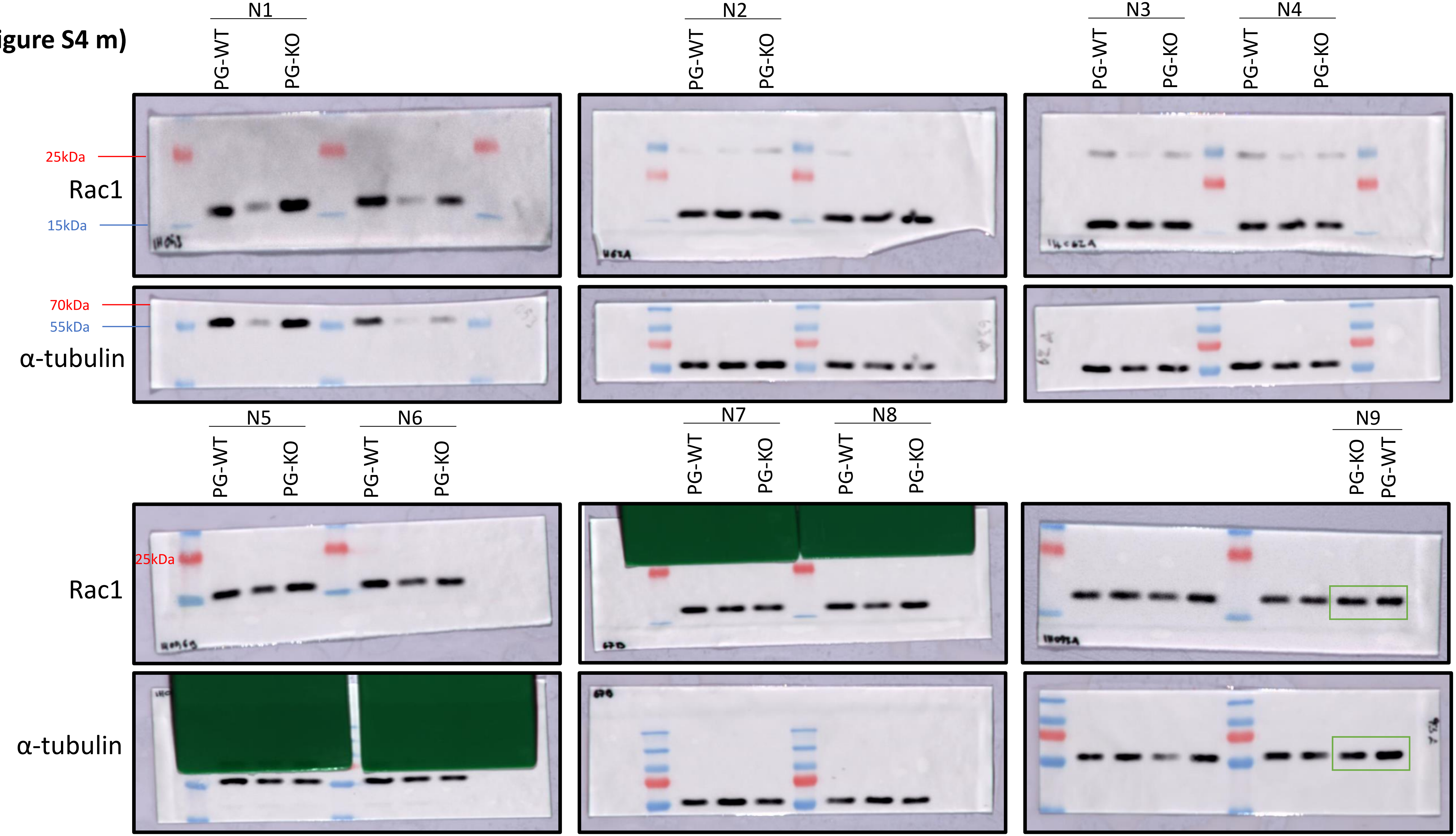
N1
PG-WT
PG-KO
N2
PG-WT
PG-KO
N3
PG-WT
PG-KO
N4
PG-WT
PG-KO
25kDa
Rac1
15kDa
70kDa
55kDa
α-tubulin
N5
PG-WT
PG-KO
N6
PG-WT
PG-KO
N7
PG-WT
PG-KO
N8
PG-WT
PG-KO
N9
PG-KO
PG-WT
25kDa
Rac1
α-tubulin

Figure S4 n)

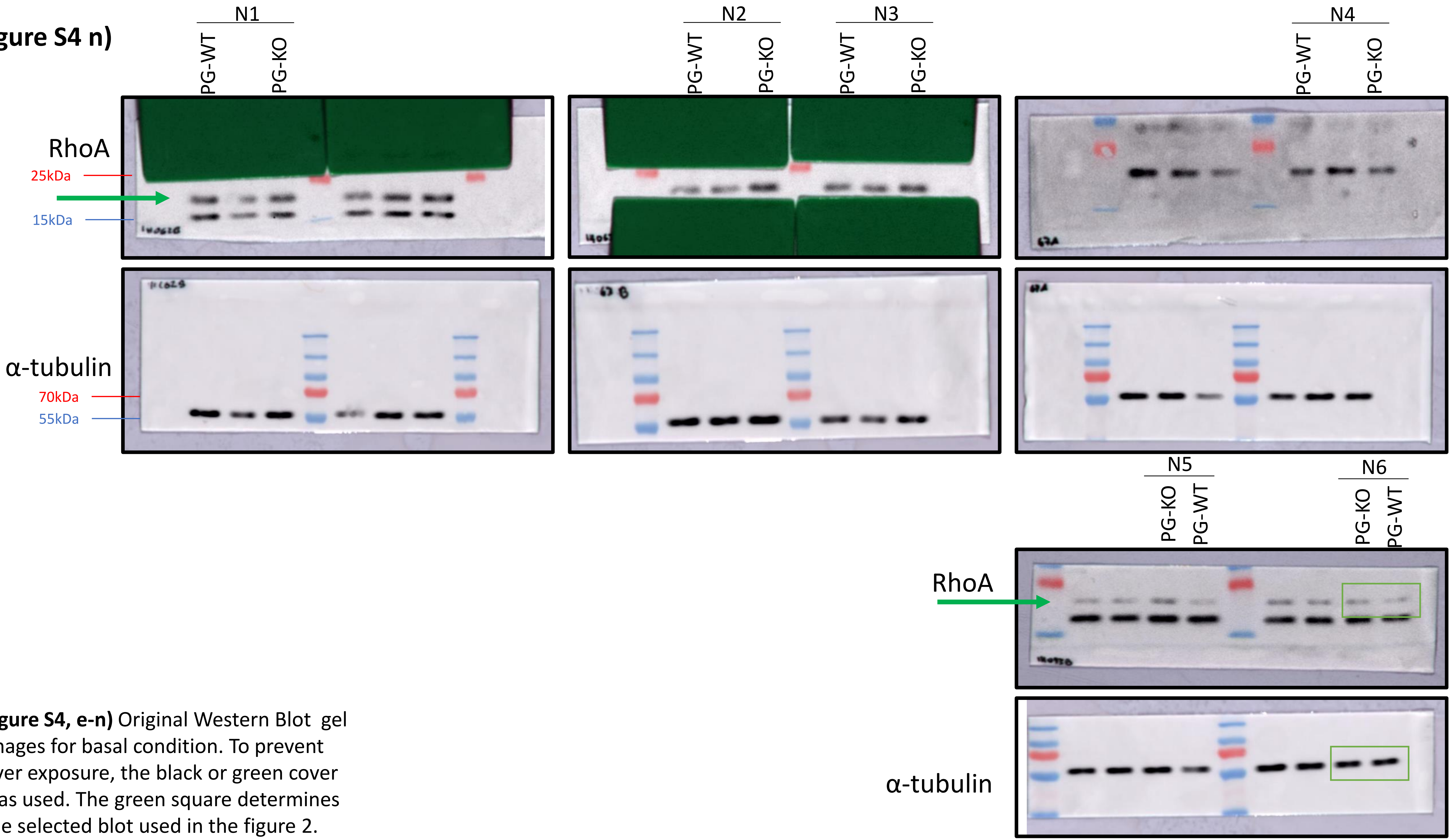

Figure S4, e-n) Original Western Blot gel images for basal condition. To prevent over exposure, the black or green cover was used. The green square determines the selected blot used in the figure 2.

Figure S4 o)

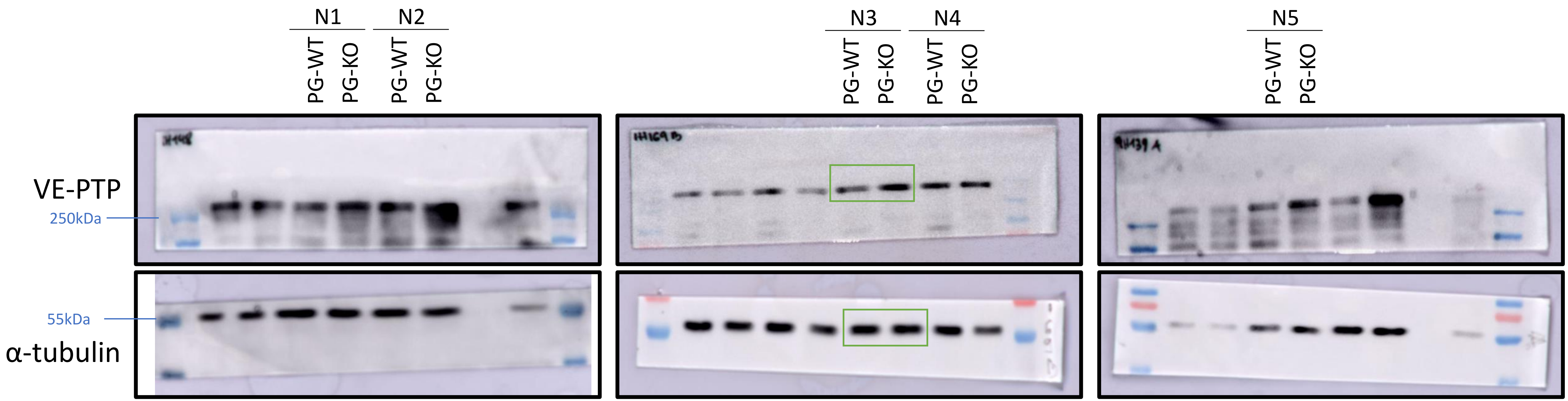

Figure S4 p)

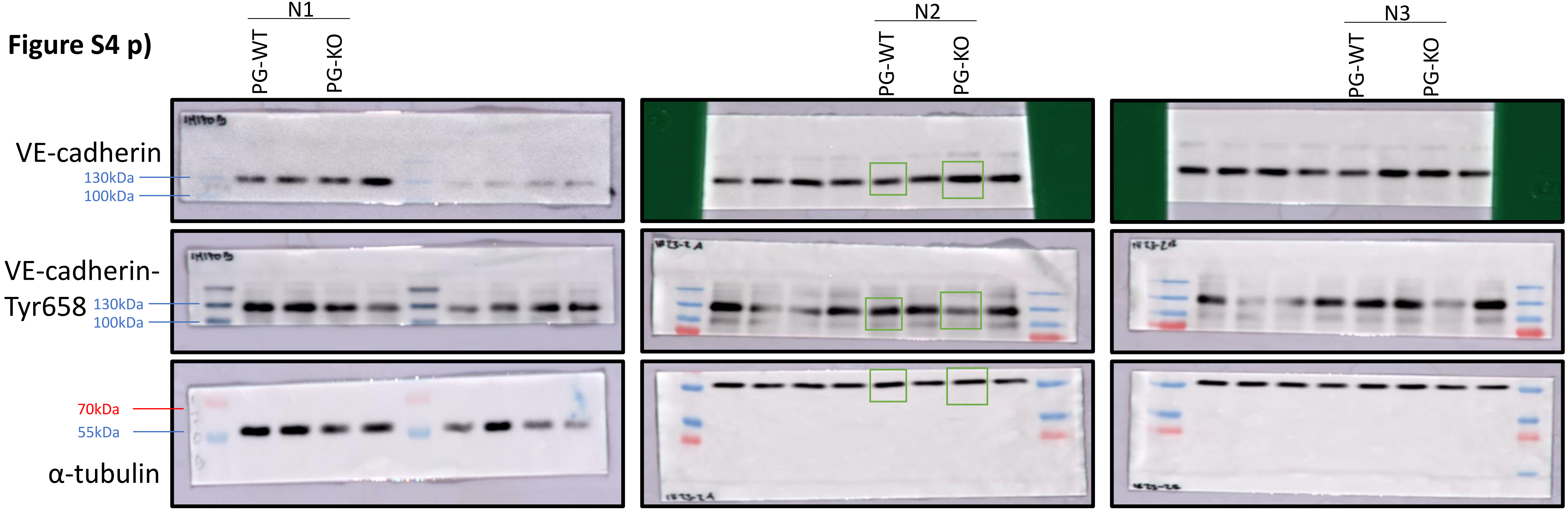

N1
PG-WT
PG-KO
N2
PG-WT
PG-KO
N3
PG-WT
PG-KO
VE-cadherin
130kDa
100kDa
VE-cadherin-Tyr658
130kDa
100kDa
70kDa
55kDa
α-tubulin

Figure S4 q)

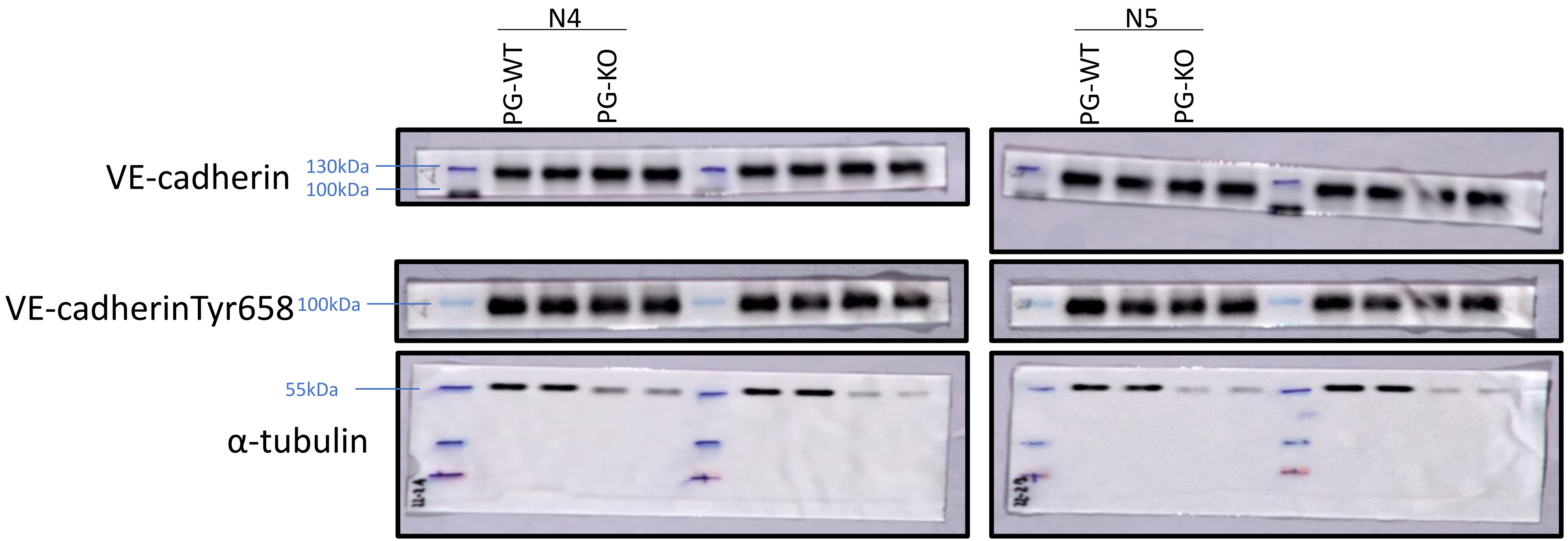

Figure S4, o-q) Original Western Blot gel images for basal condition. To prevent over exposure, the black or green cover was used. The green square determines the selected blot used in the figure 3.

Figure S4 r)

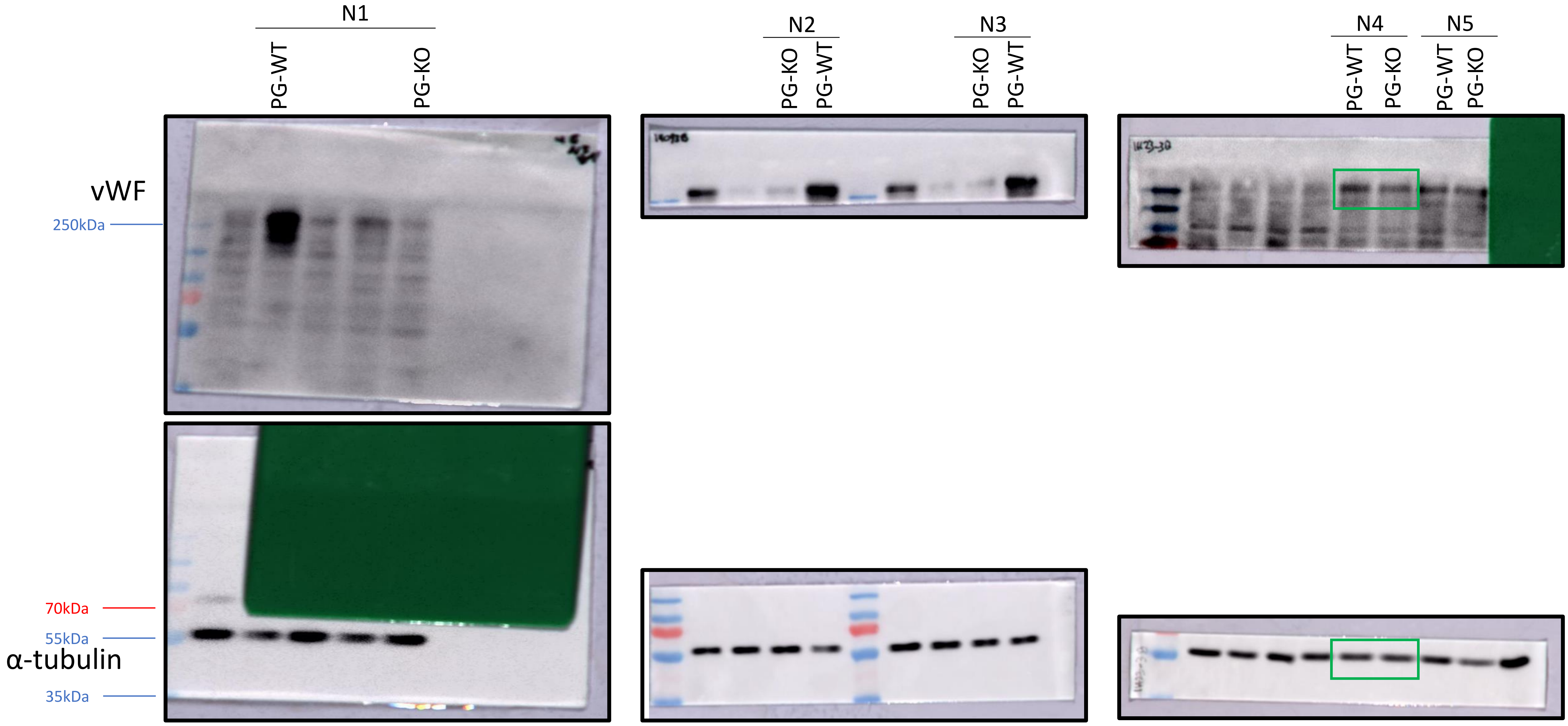

N1
PG-WT
PG-KO
N2
PG-KO
PG-WT
N3
PG-KO
PG-WT
N4
PG-WT
PG-KO
N5
PG-WT
PG-KO
vWF
250kDa
70kDa
55kDa
α-tubulin
35kDa

Figure S4 s)

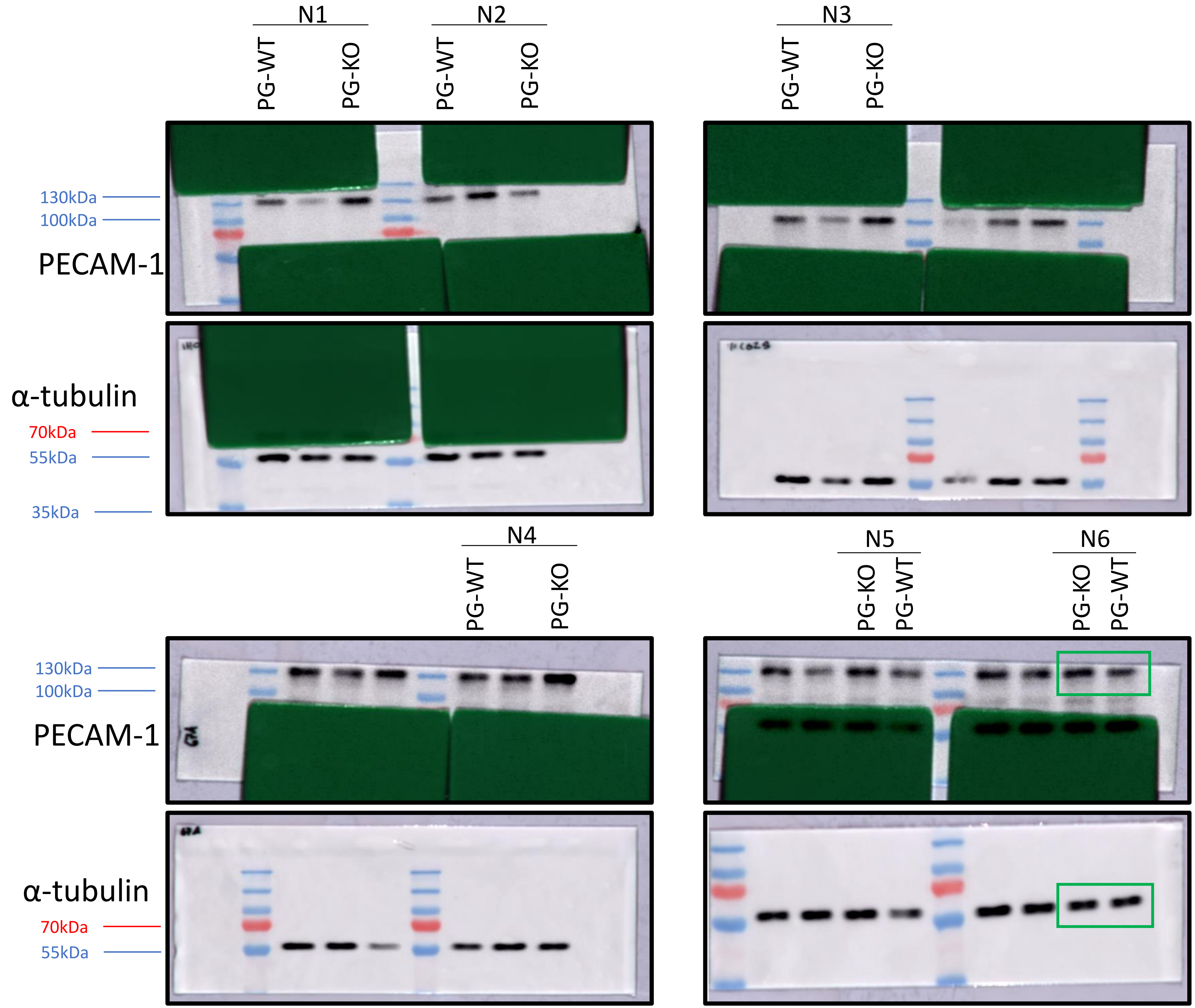

Figure S4, r-s) Original Western Blot gel images for basal condition. To prevent over exposure, the black or green cover was used. The green square determines the selected blot used in the figure S1.

Figure S4 t)

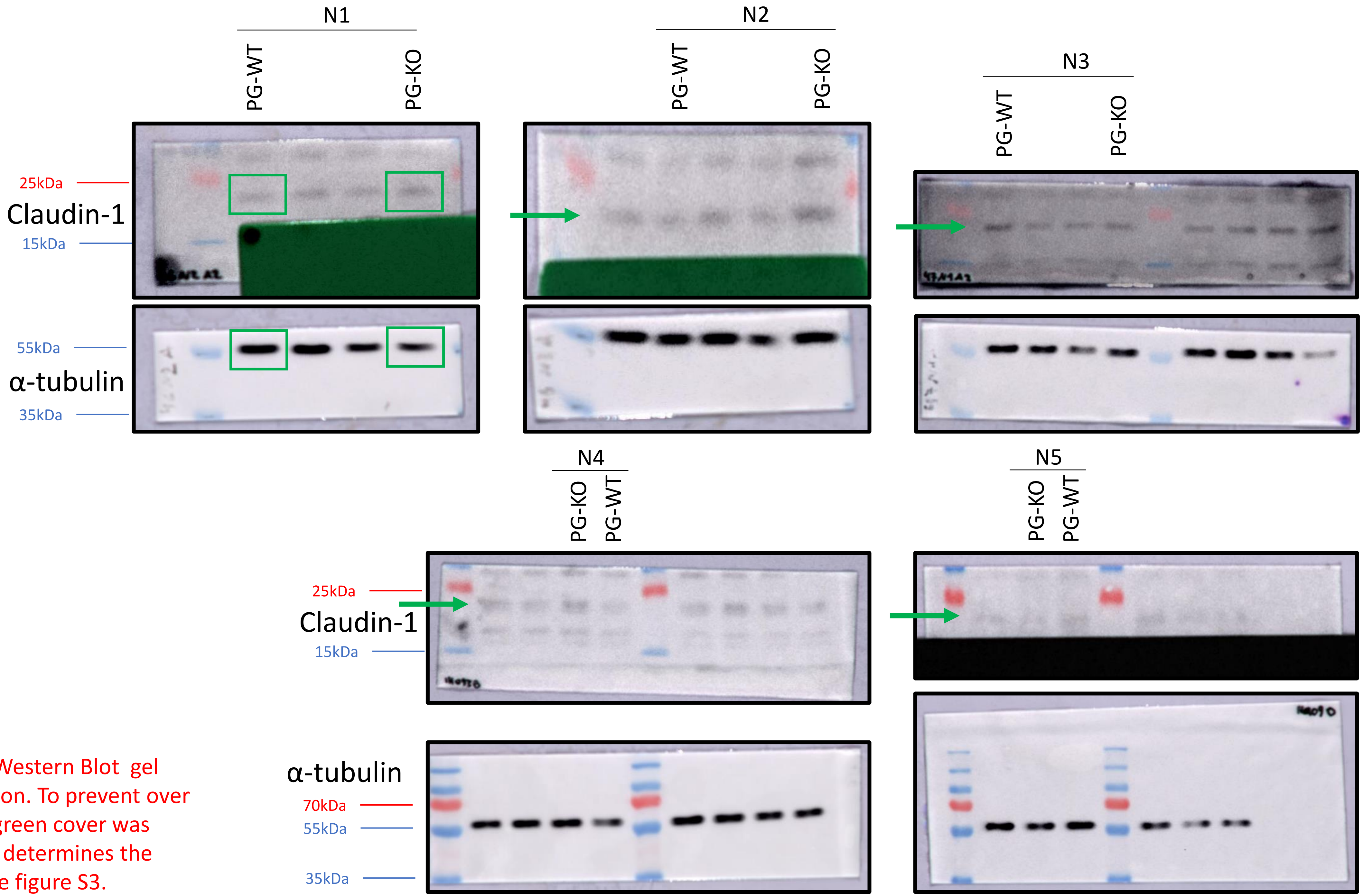

Figure S4, s-t) Original Western Blot gel images for basal condition. To prevent over exposure, the black or green cover was used. The green square determines the selected blot used in the figure S3.

Original PCR gel images

Figure S5 a)

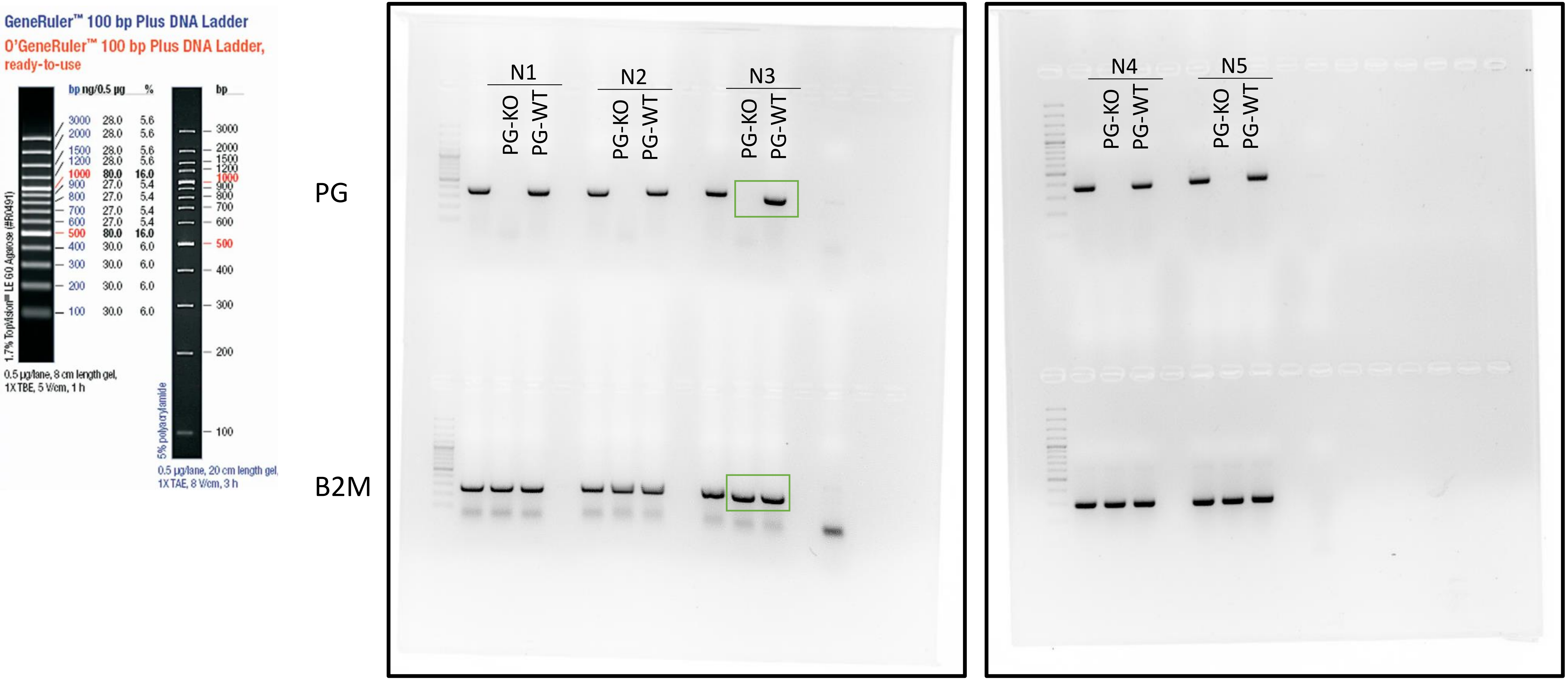
GeneRuler™ 100 bp Plus DNA Ladder
O'GeneRuler™ 100 bp Plus DNA Ladder, ready-to-use
bp ng/0.5 µg %
3000 28.0 5.6
2000 28.0 5.6
1500 28.0 5.6
1200 28.0 5.6
1000 80.0 16.0
900 27.0 5.4
800 27.0 5.4
700 27.0 5.4
600 27.0 5.4
500 80.0 16.0
400 30.0 6.0
300 30.0 6.0
200 30.0 6.0
100 30.0 6.0
1.7% TopVision™ LE GQ Agarose (#R0491)
0.5 µg/lane, 8 cm length gel, 1X TBE, 5 V/cm, 1 h
bp
3000
2000
1500
1200
1000
900
800
700
600
500
400
300
200
100
5% polyacrylamide
0.5 µg/lane, 20 cm length gel, 1X TAE, 8 V/cm, 3 h
PG
B2M
N1
N2
N3
N4
N5
PG-KO
PG-WT

Figure S5 b)

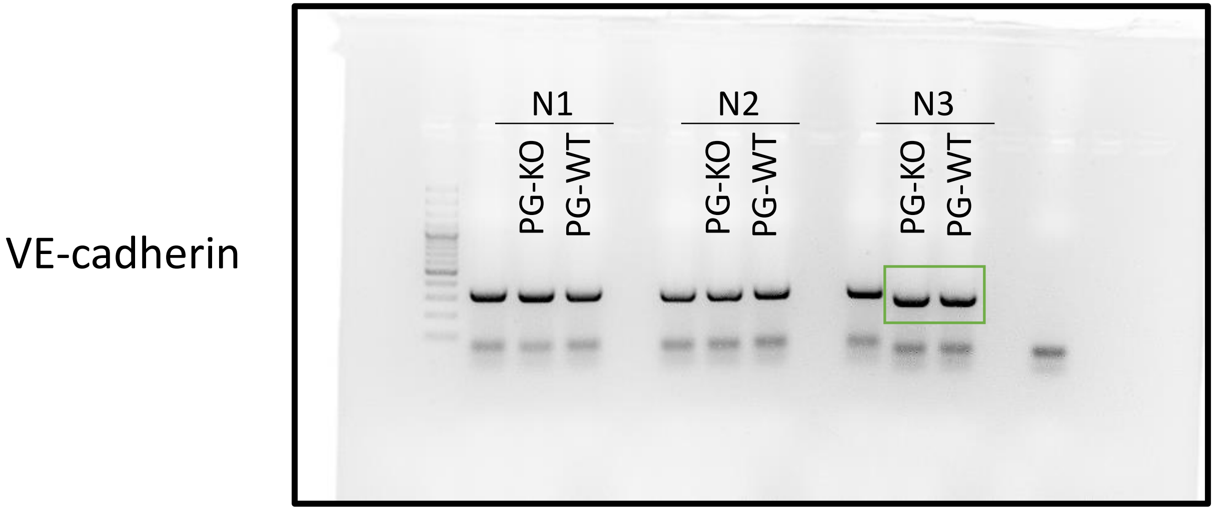
VE-cadherin
N1
PG-KO
PG-WT
N2
PG-KO
PG-WT
N3
PG-KO
PG-WT

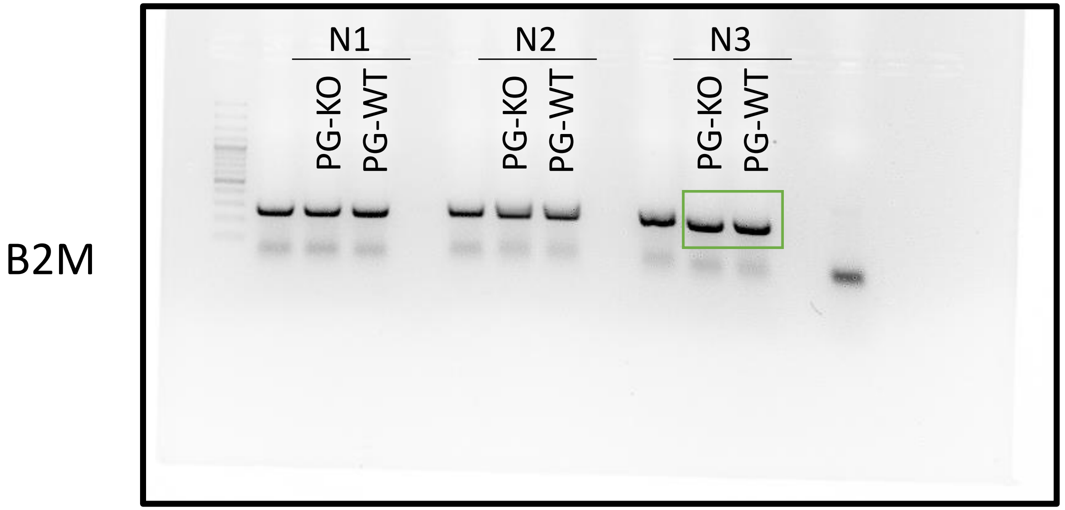
B2M
N1
PG-KO
PG-WT
N2
PG-KO
PG-WT
N3
PG-KO
PG-WT

Figure S5 c)

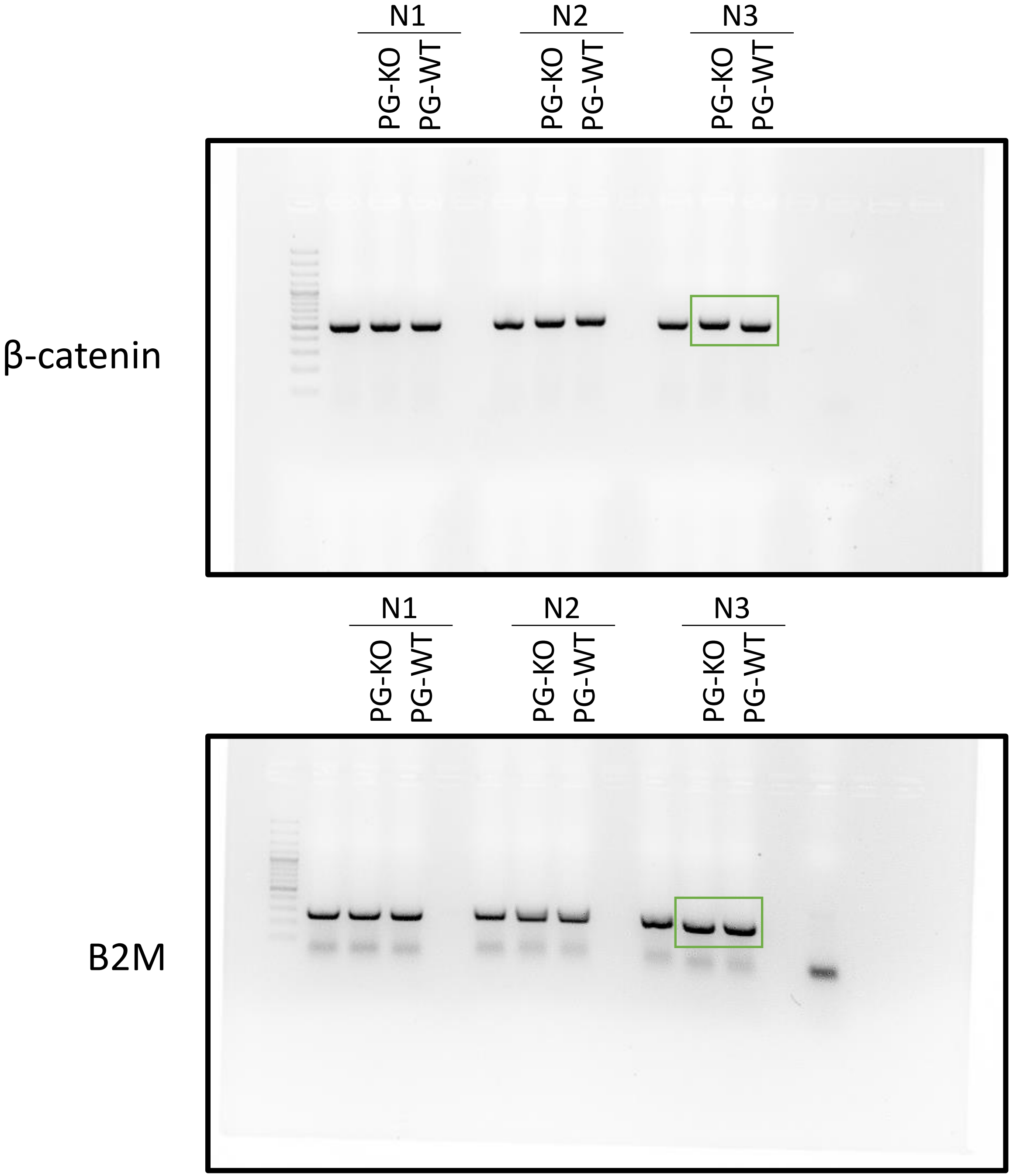
N1
N2
N3
PG-KO
PG-WT
PG-KO
PG-WT
PG-KO
PG-WT
β-catenin
N1
N2
N3
PG-KO
PG-WT
PG-KO
PG-WT
PG-KO
PG-WT
B2M

Figure S5 d)

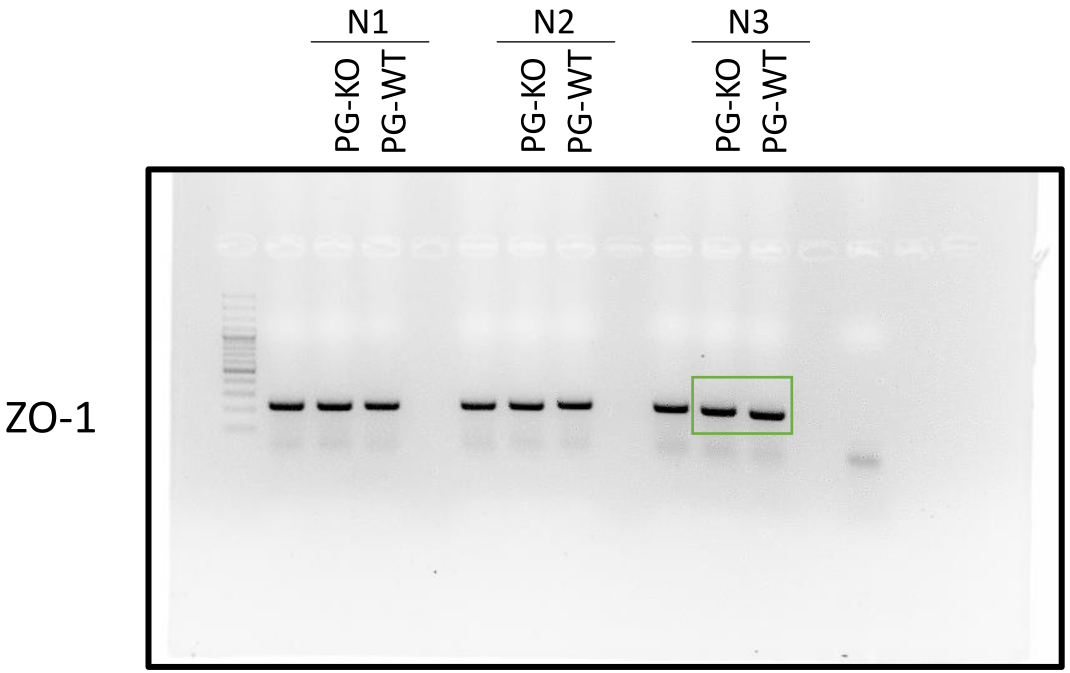
N1
N2
N3
PG-KO
PG-WT
PG-KO
PG-WT
PG-KO
PG-WT
ZO-1

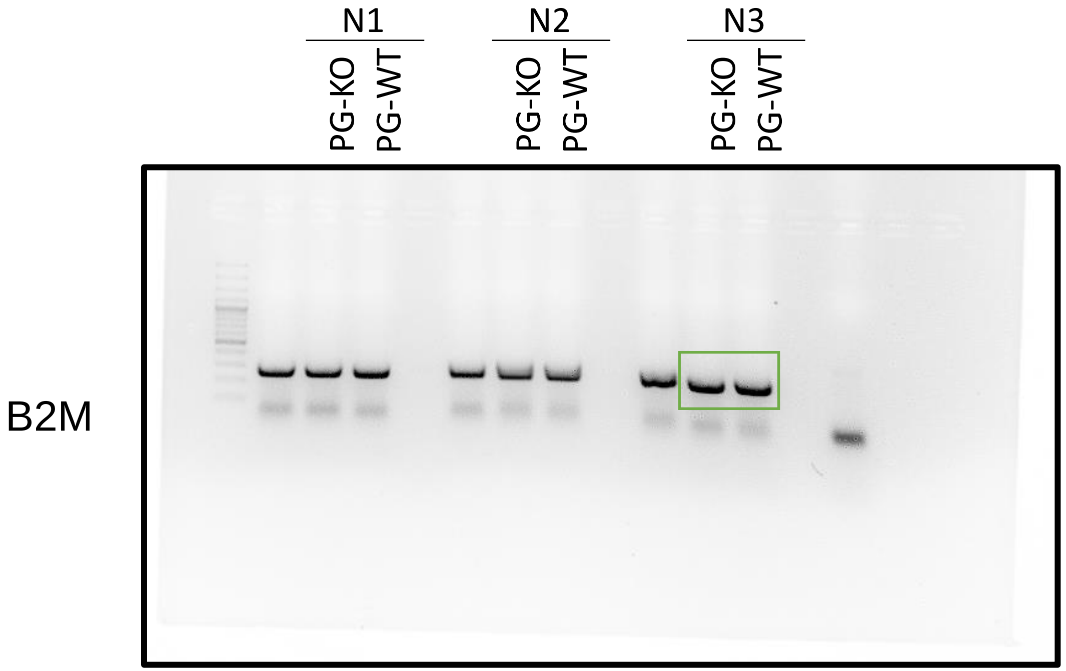
N1
N2
N3
PG-KO
PG-WT
PG-KO
PG-WT
PG-KO
PG-WT
B2M

Figure S5 e)

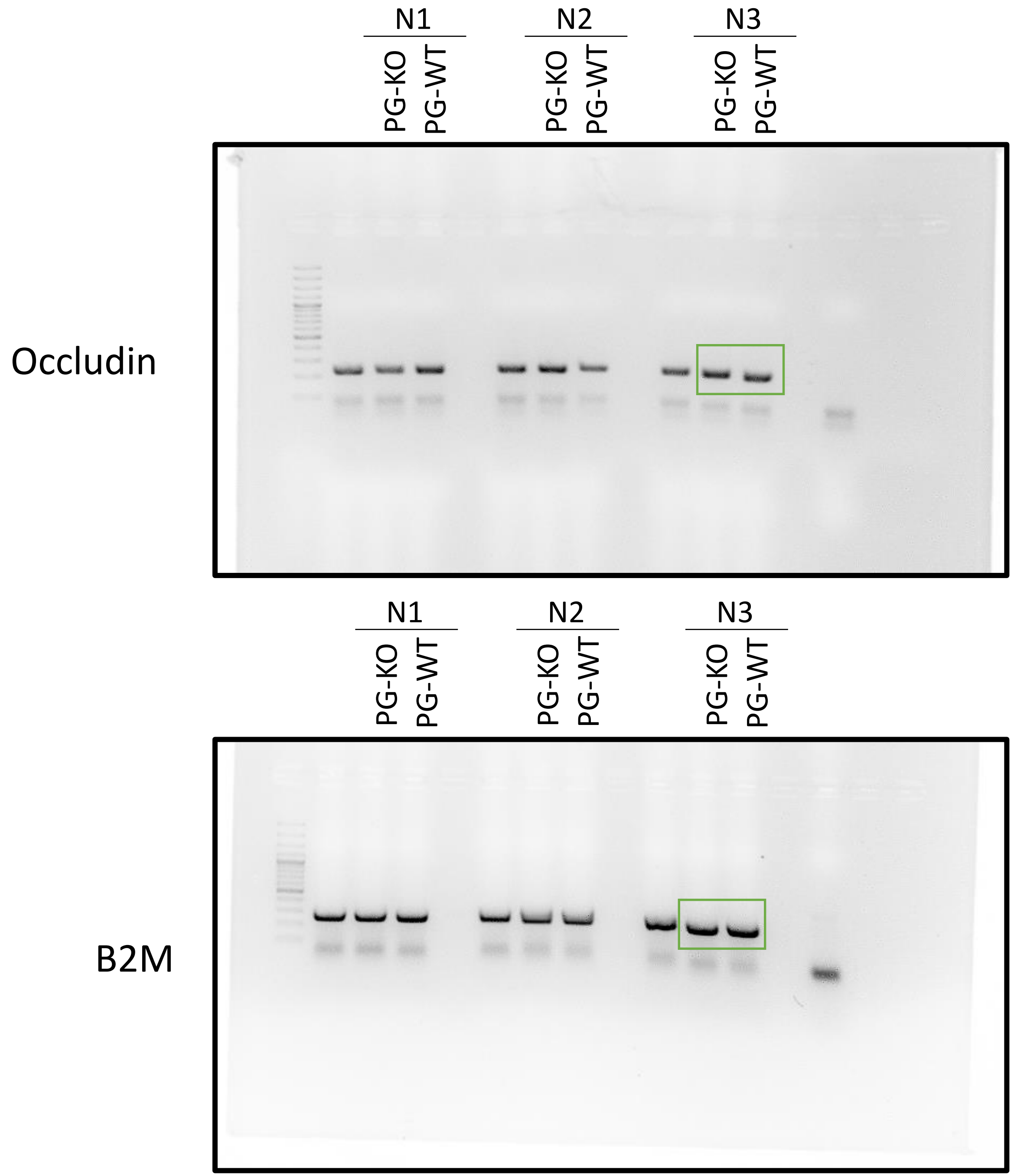
N1
PG-KO
PG-WT
N2
PG-KO
PG-WT
N3
PG-KO
PG-WT
Occludin
N1
PG-KO
PG-WT
N2
PG-KO
PG-WT
N3
PG-KO
PG-WT
B2M

Figure S5 f)

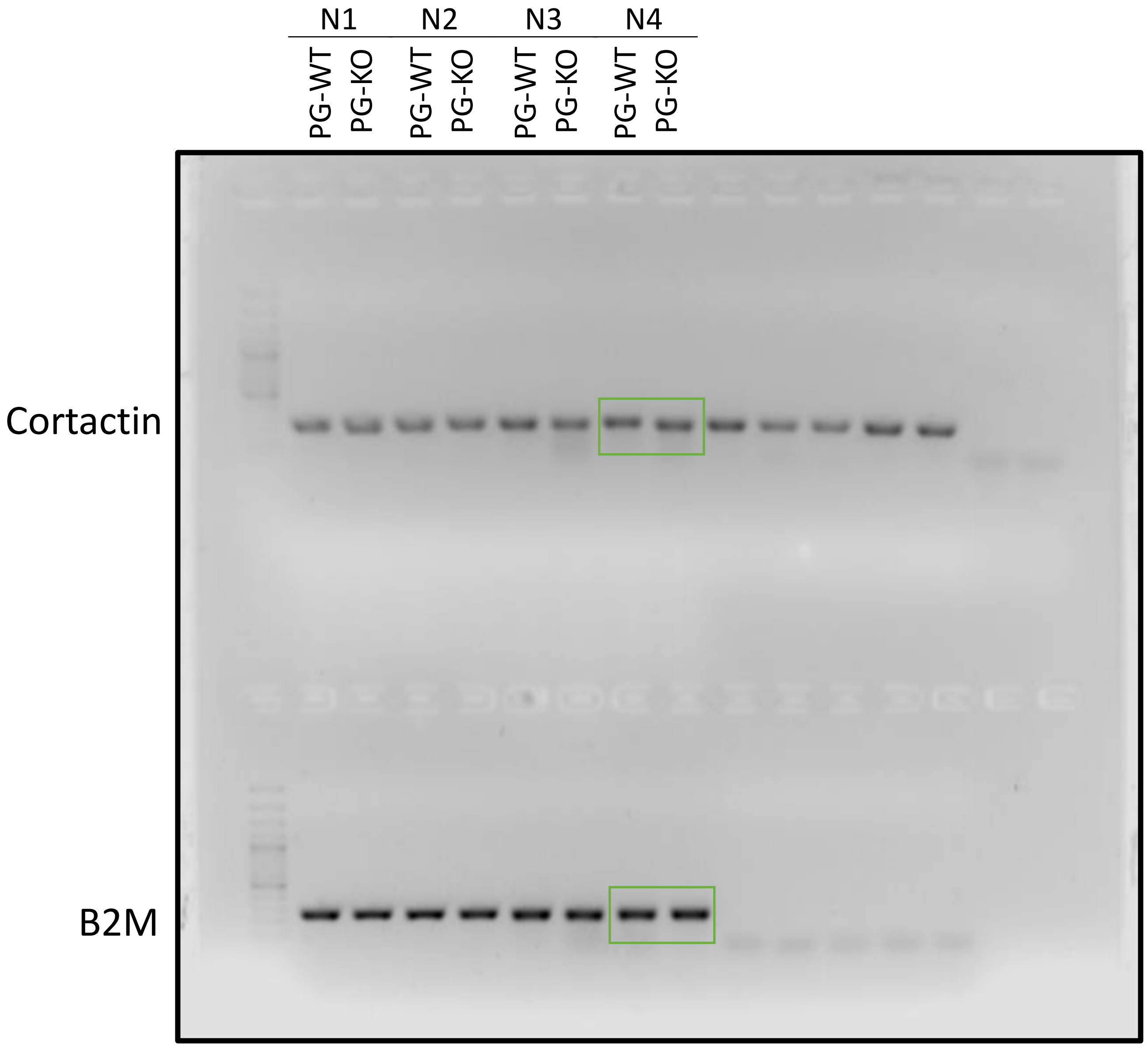

Figure S5, a-f) Original gel images for PCR. The green square determines the selected blot used in the figure 2.

Figure S5 g)

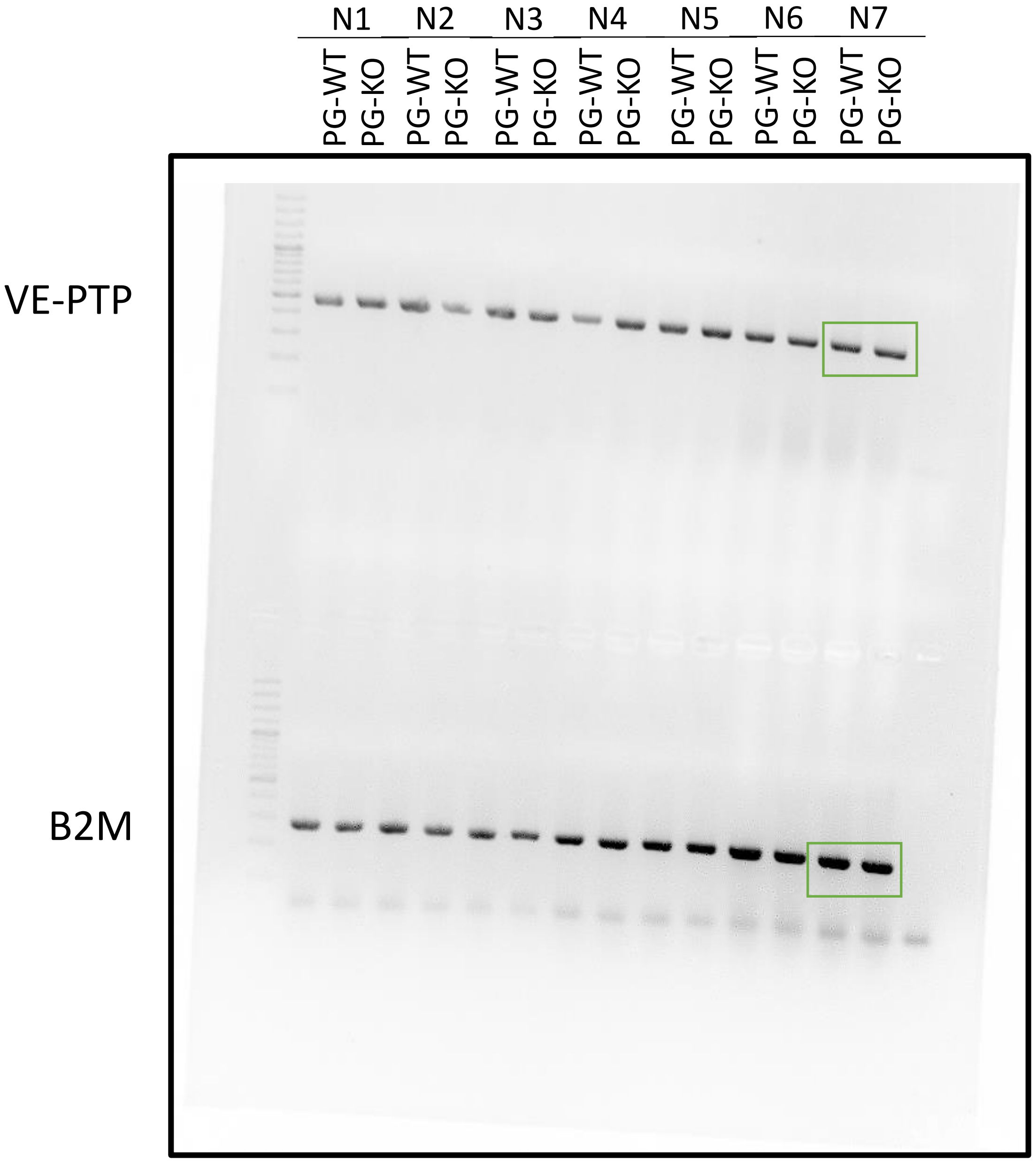

Figure S5, g) Original gel images for PCR. The green square determines the selected blot used in the figure 3.

Figure S5 h)

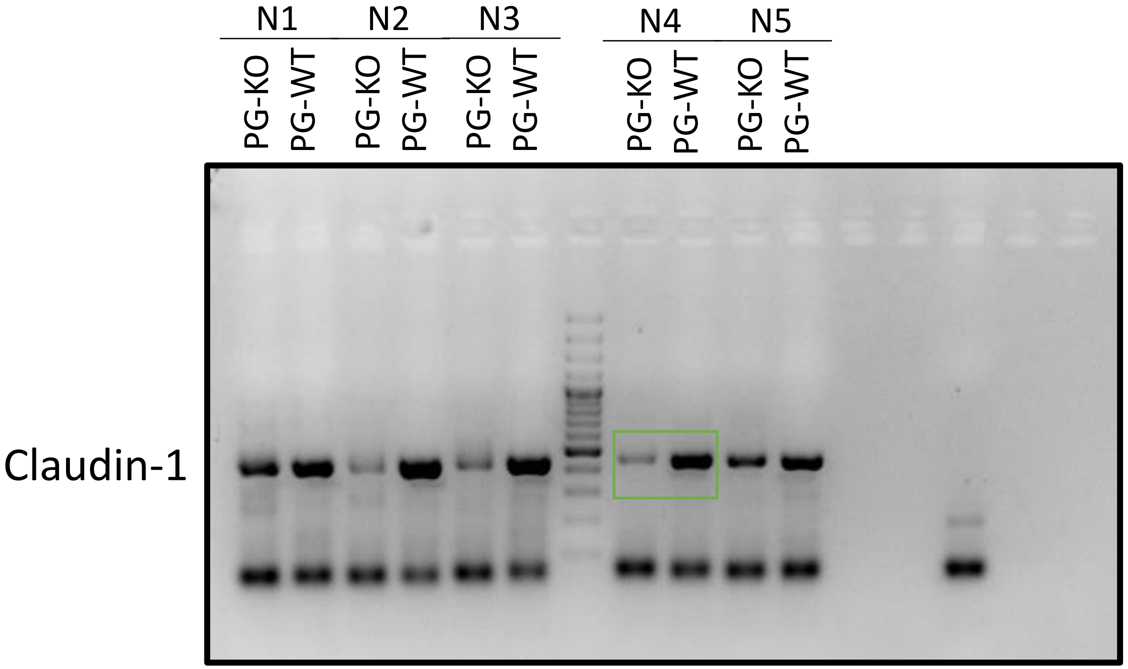
N1 N2 N3 N4 N5
PG-KO PG-WT PG-KO PG-WT PG-KO PG-WT PG-KO PG-WT PG-KO PG-WT
Claudin-1

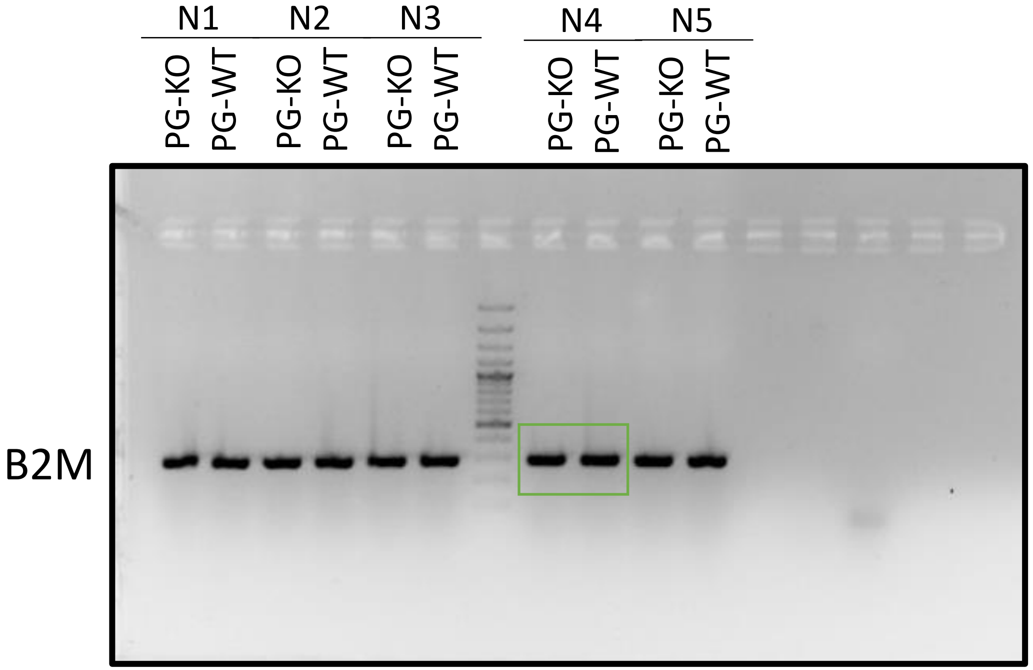
N1 N2 N3 N4 N5
PG-KO PG-WT PG-KO PG-WT PG-KO PG-WT PG-KO PG-WT PG-KO PG-WT
B2M

Figure S5 i)

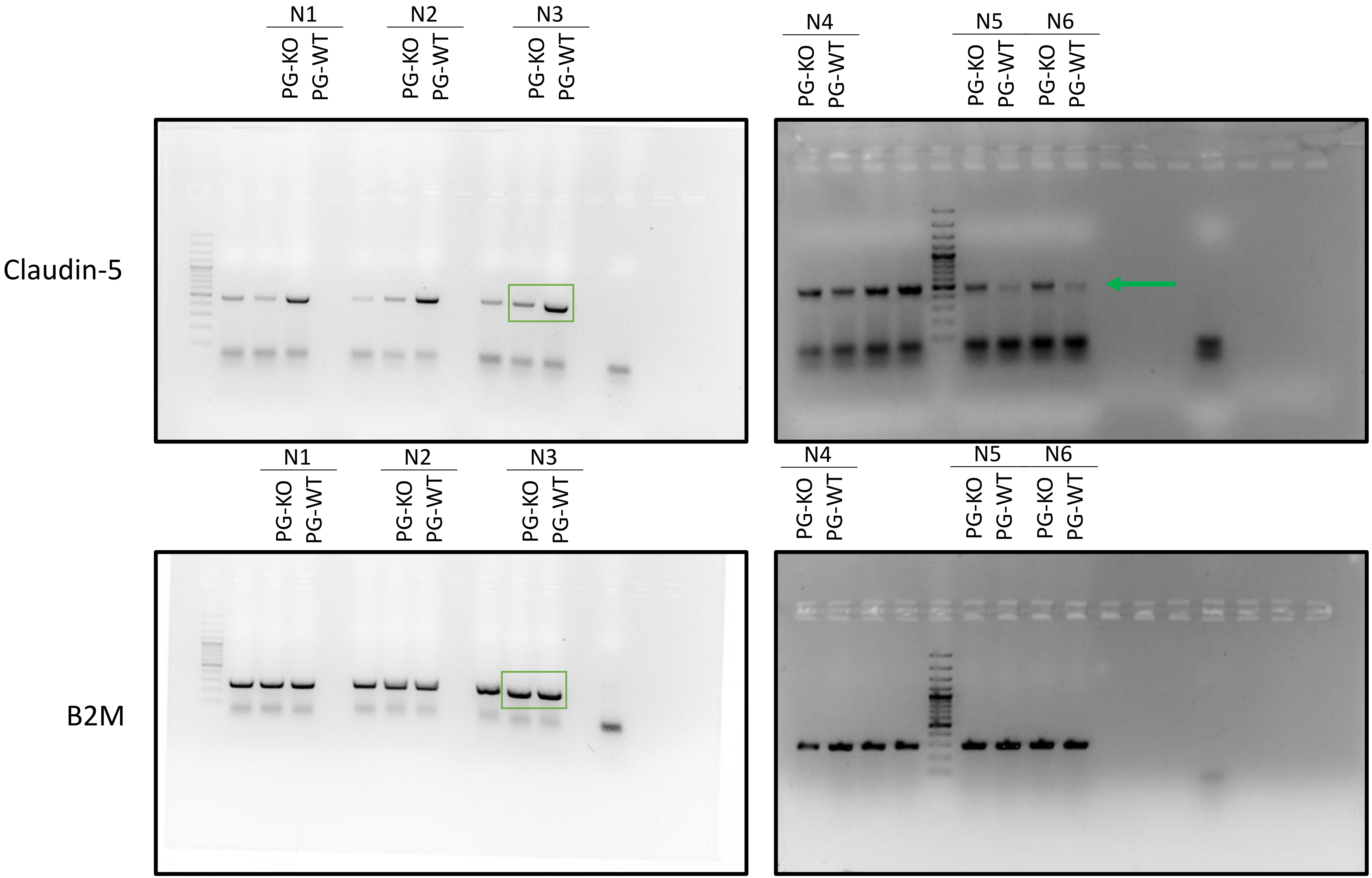

Figure S5 j)

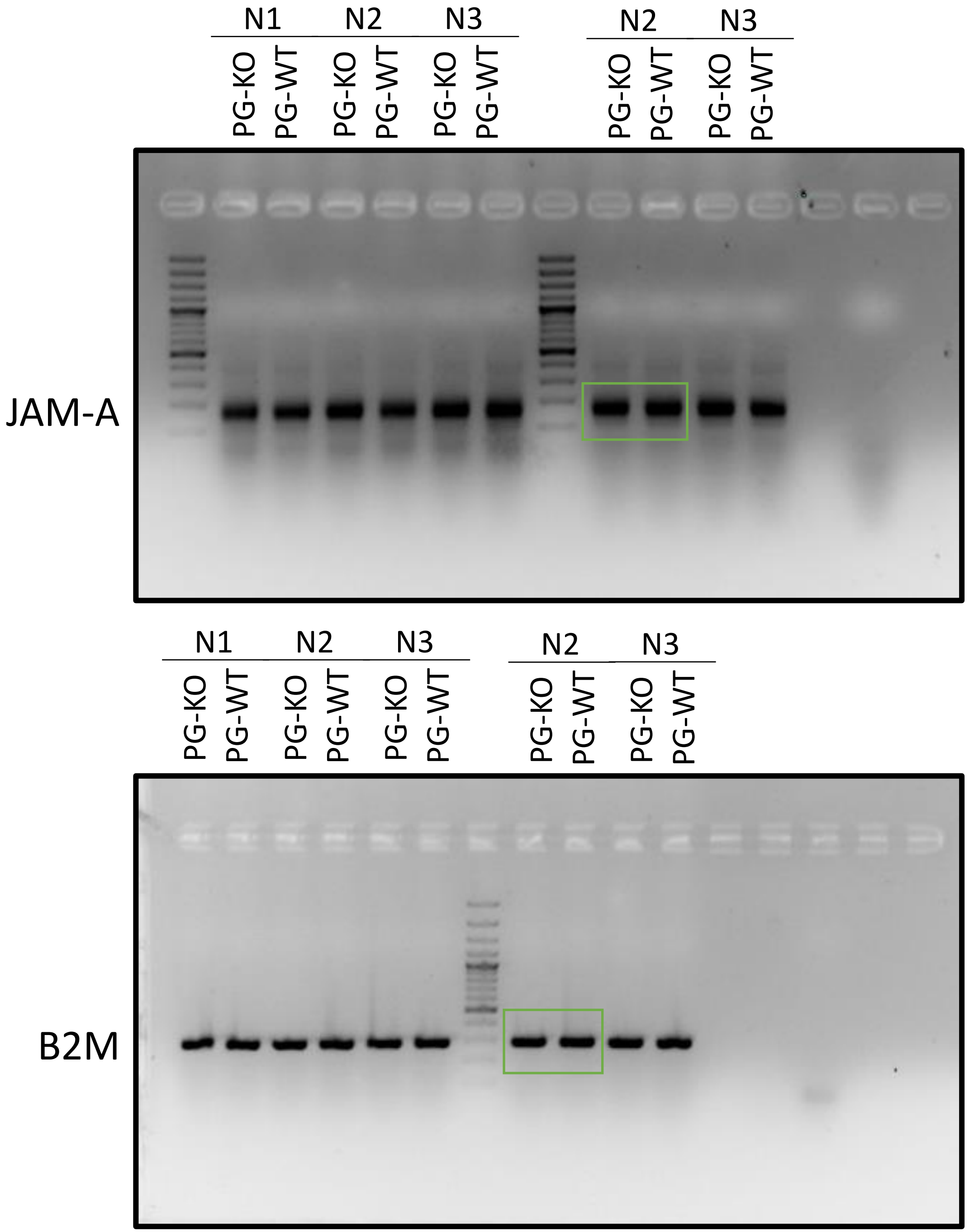
N1
N2
N3
N2
N3
PG-KO
PG-WT
PG-KO
PG-WT
PG-KO
PG-WT
PG-KO
PG-WT
PG-KO
PG-WT
JAM-A
N1
N2
N3
N2
N3
PG-KO
PG-WT
PG-KO
PG-WT
PG-KO
PG-WT
PG-KO
PG-WT
PG-KO
PG-WT
B2M

Figure S5 k)

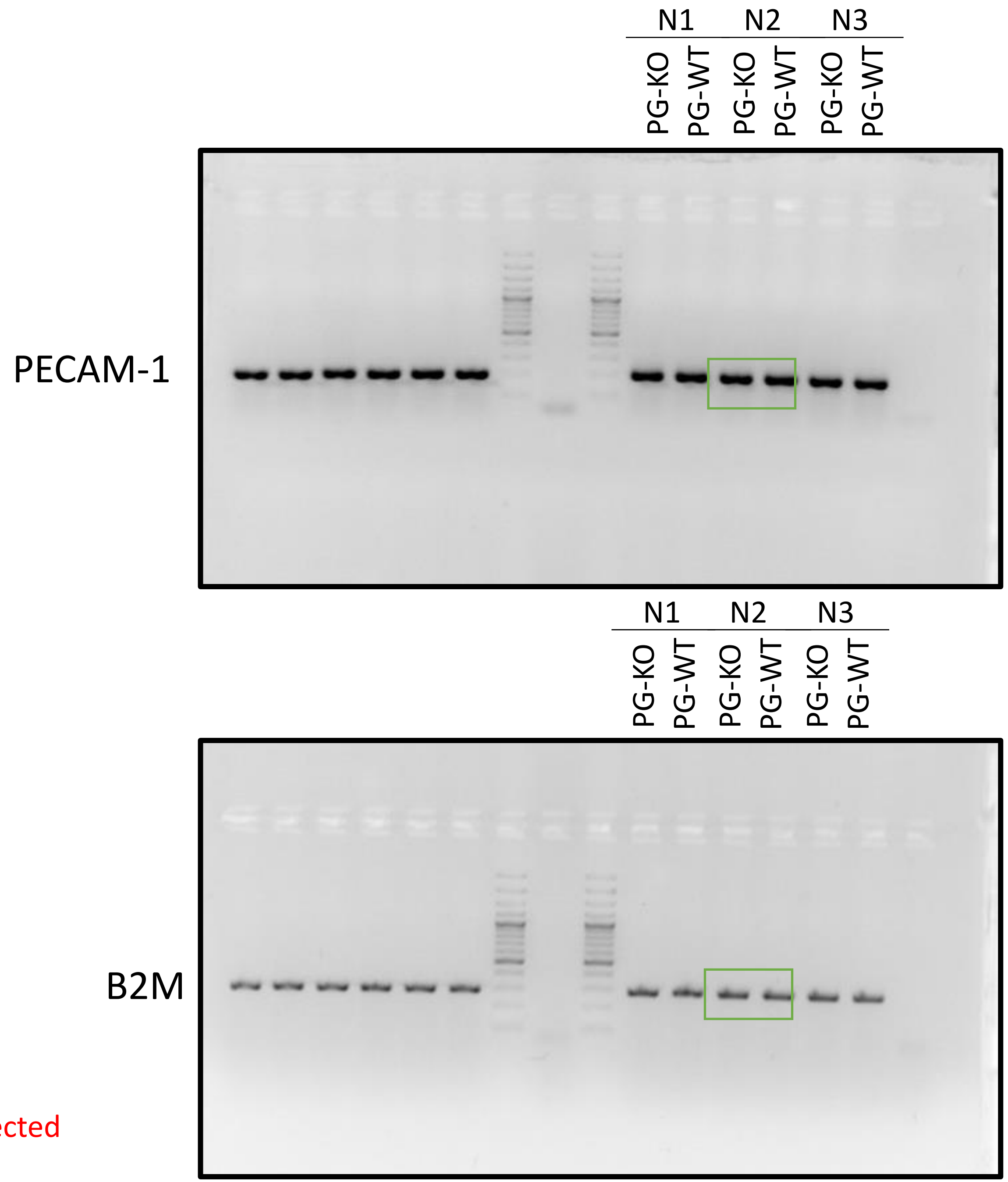

Figure S5, h-k) Original gel images for PCR. The green square determines the selected blot used in the figure S3.

Original western blot gel images for vehicle or F/R treatment

Figure S6 a)

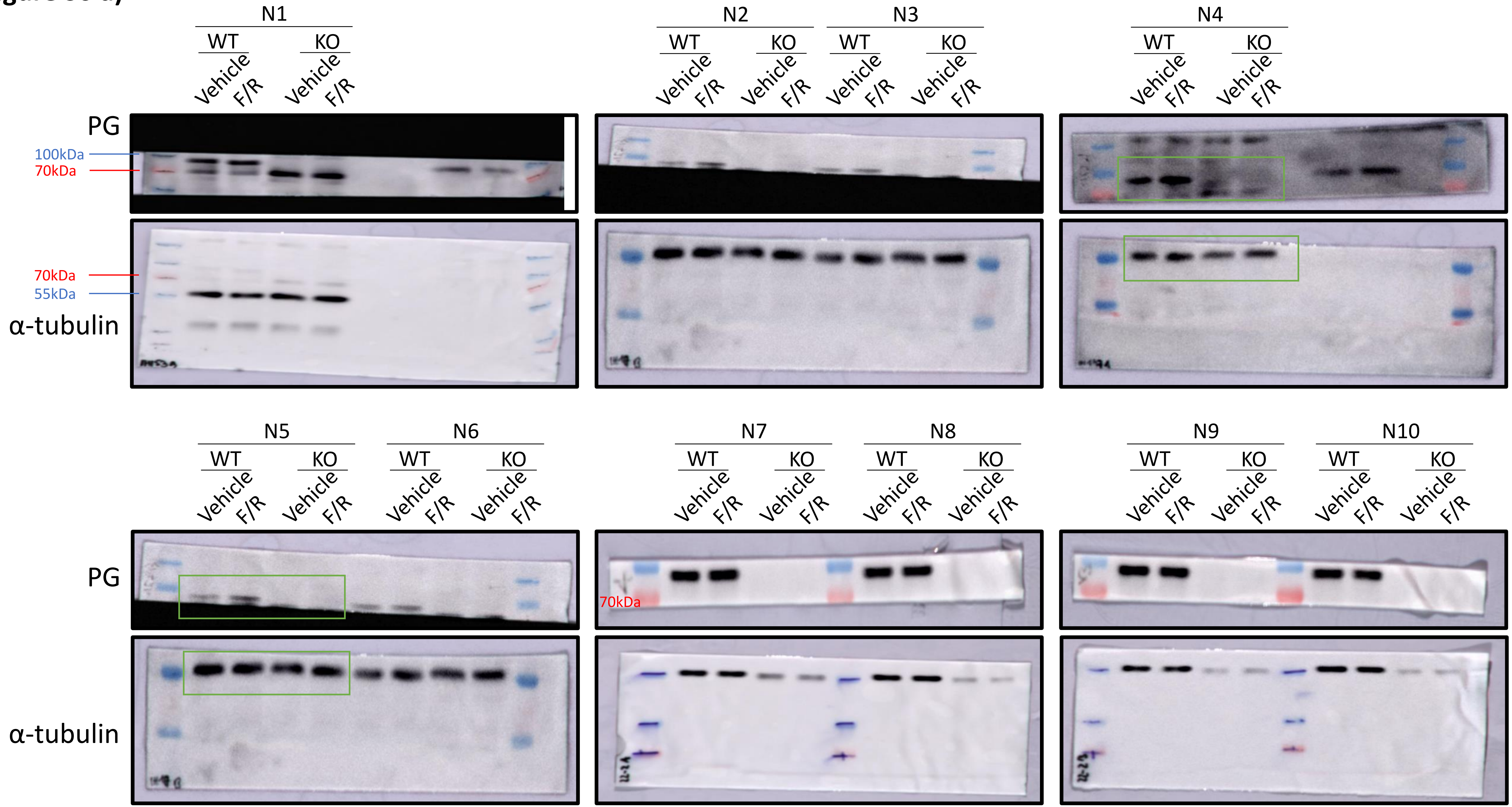
N1
N2
N3
N4
WT
KO
Vehicle
F/R
PG
100kDa
70kDa
55kDa
α-tubulin
N5
N6
N7
N8
N9
N10

Figure S6 b)

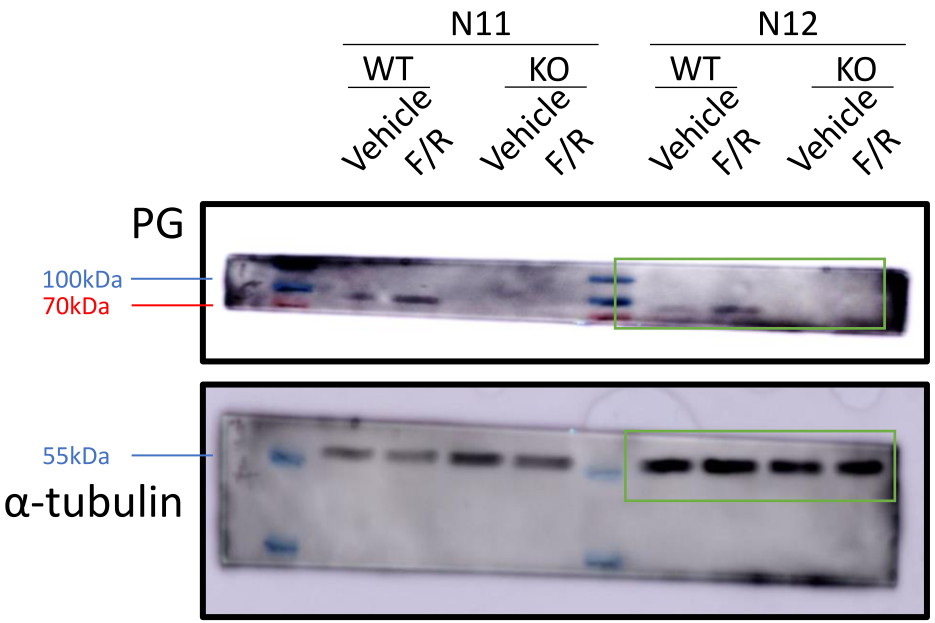

N11
N12
WT
KO
WT
KO
Vehicle
F/R
Vehicle
F/R
Vehicle
F/R
Vehicle
F/R
PG
100kDa
70kDa
55kDa
α-tubulin

Figure S6 c)

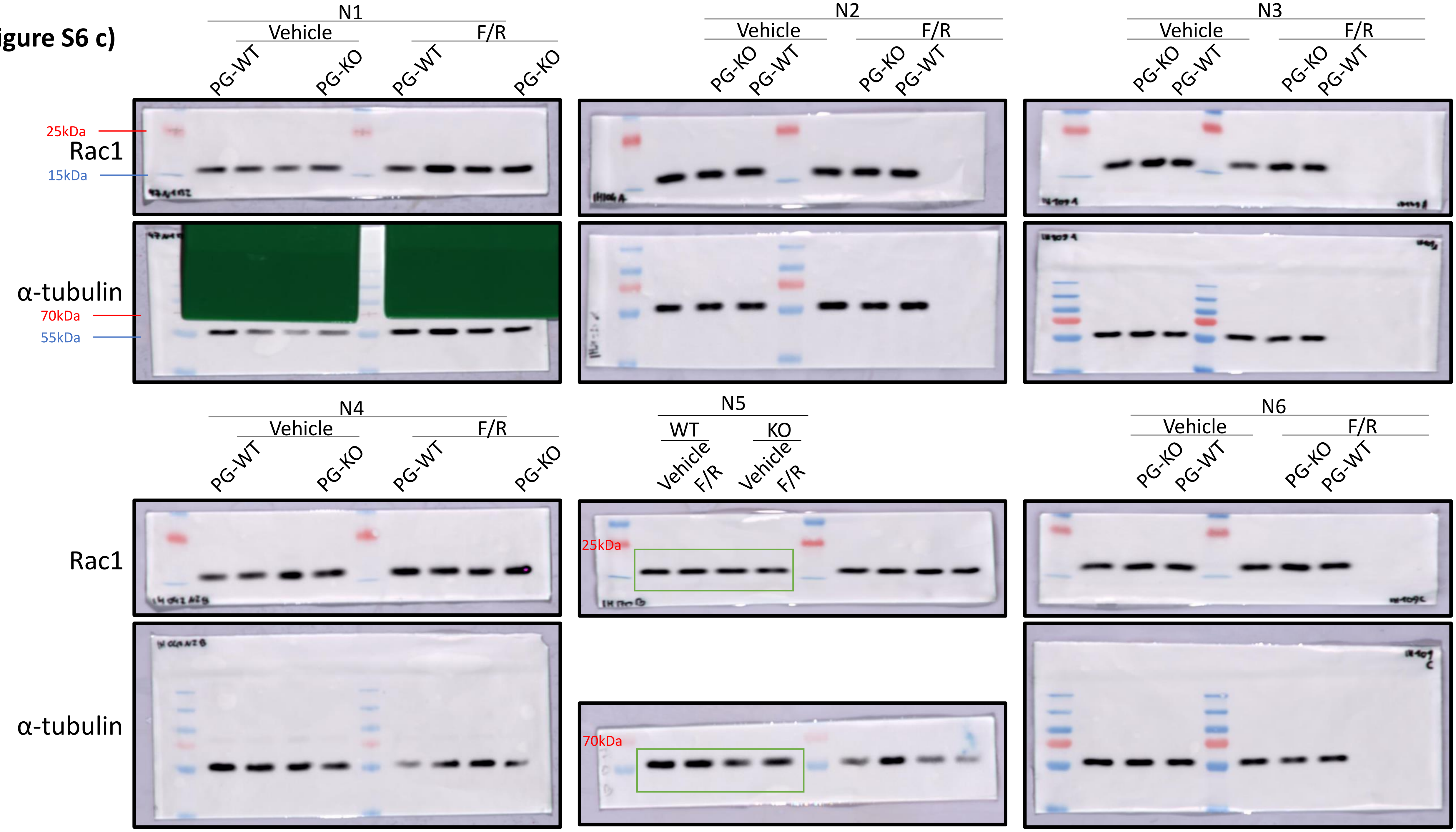

N1
Vehicle
F/R
PG-WT
PG-KO
PG-WT
PG-KO
N2
Vehicle
F/R
PG-KO
PG-WT
PG-KO
PG-WT
N3
Vehicle
F/R
PG-KO
PG-WT
PG-KO
PG-WT
25kDa
Rac1
15kDa
α-tubulin
70kDa
55kDa
N4
Vehicle
F/R
PG-WT
PG-KO
PG-WT
PG-KO
N5
WT
KO
Vehicle
F/R
Vehicle
F/R
N6
Vehicle
F/R
PG-KO
PG-WT
PG-KO
PG-WT
Rac1
25kDa
α-tubulin
70kDa

Figure S6 d)

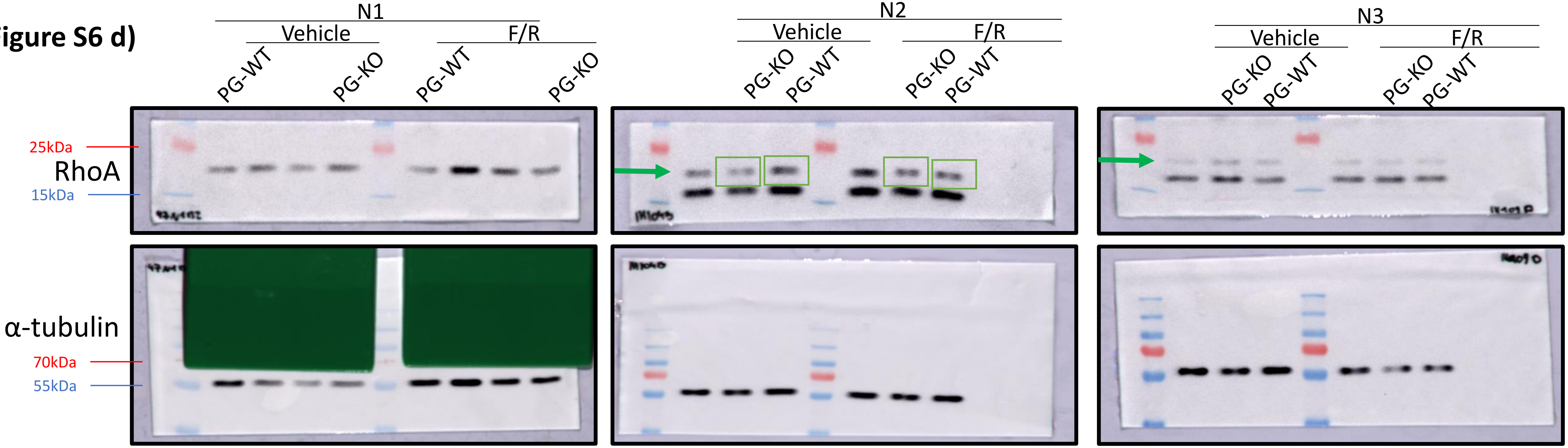
N1
Vehicle
F/R
PG-WT
PG-KO
PG-WT
PG-KO
N2
Vehicle
F/R
PG-KO
PG-WT
PG-KO
PG-WT
N3
Vehicle
F/R
PG-KO
PG-WT
PG-KO
PG-WT
25kDa
RhoA
15kDa
α-tubulin
70kDa
55kDa

Figure S6 e)

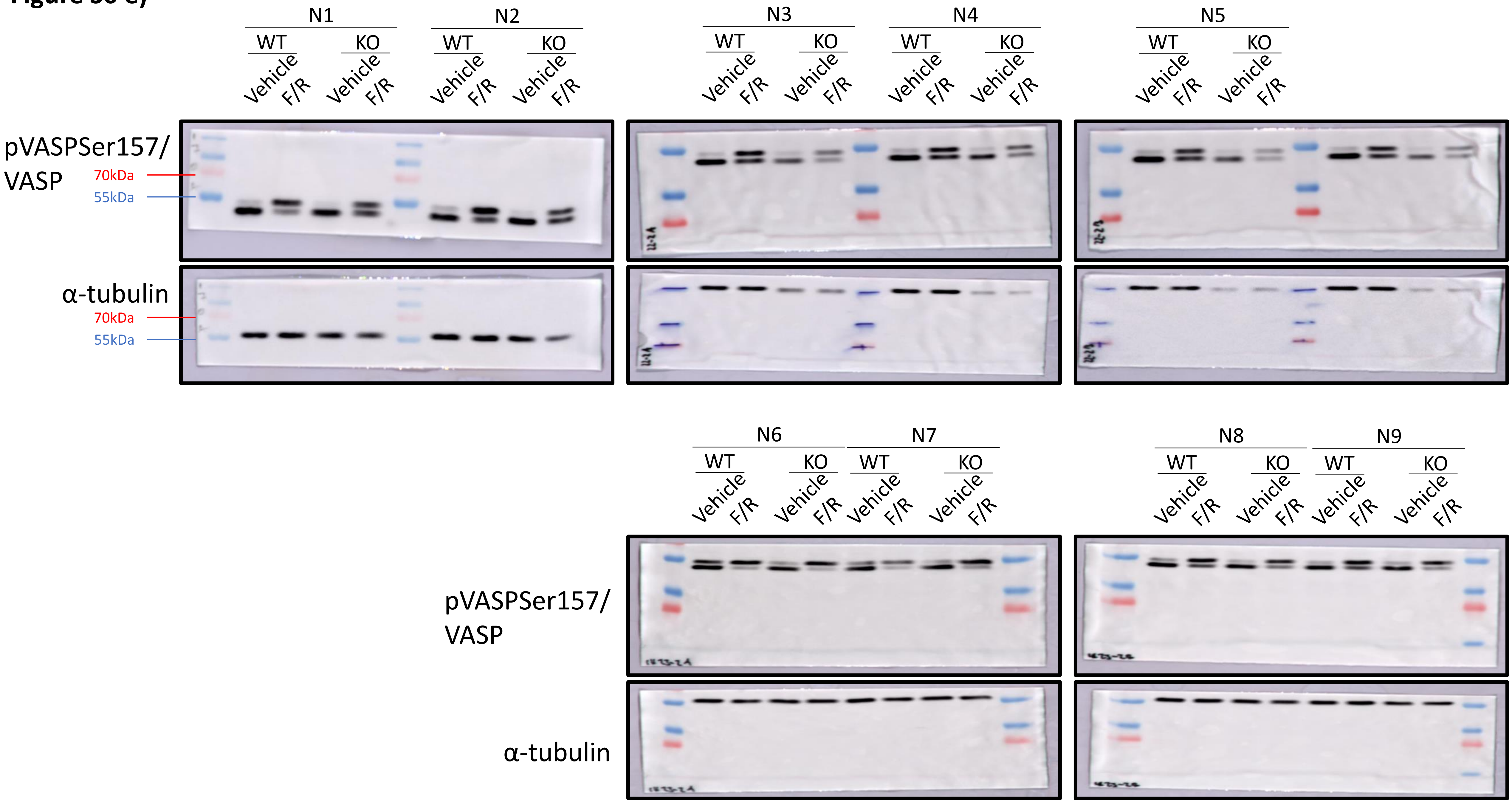
N1
N2
N3
N4
N5
N6
N7
N8
N9
WT
KO
Vehicle
F/R
pVASPSer157/
VASP
70kDa
55kDa
α-tubulin

Figure S6 f)

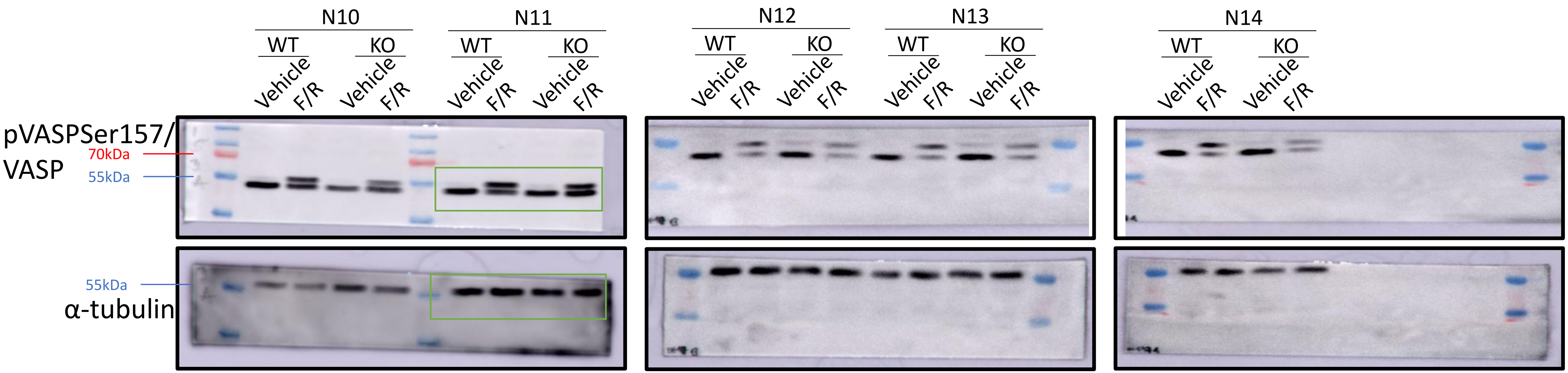

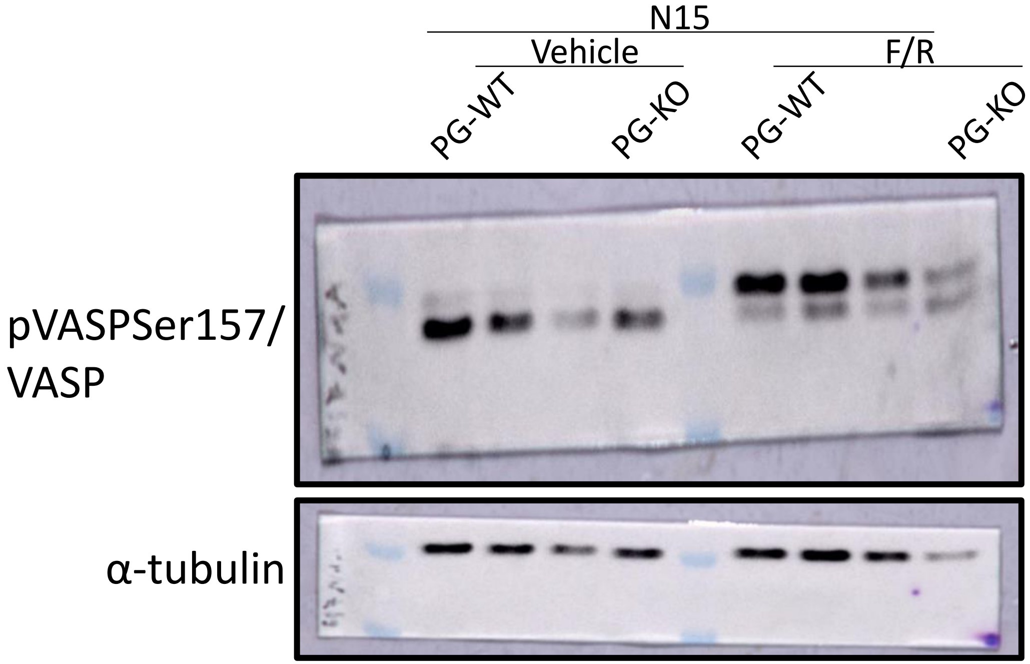

Figure S6, a-f) Original Western Blot gel images for vehicle or F/R treatment. To prevent over exposure, the black or green cover was used. The green square determines the selected blot used in the figure 4.

Figure S6 g)

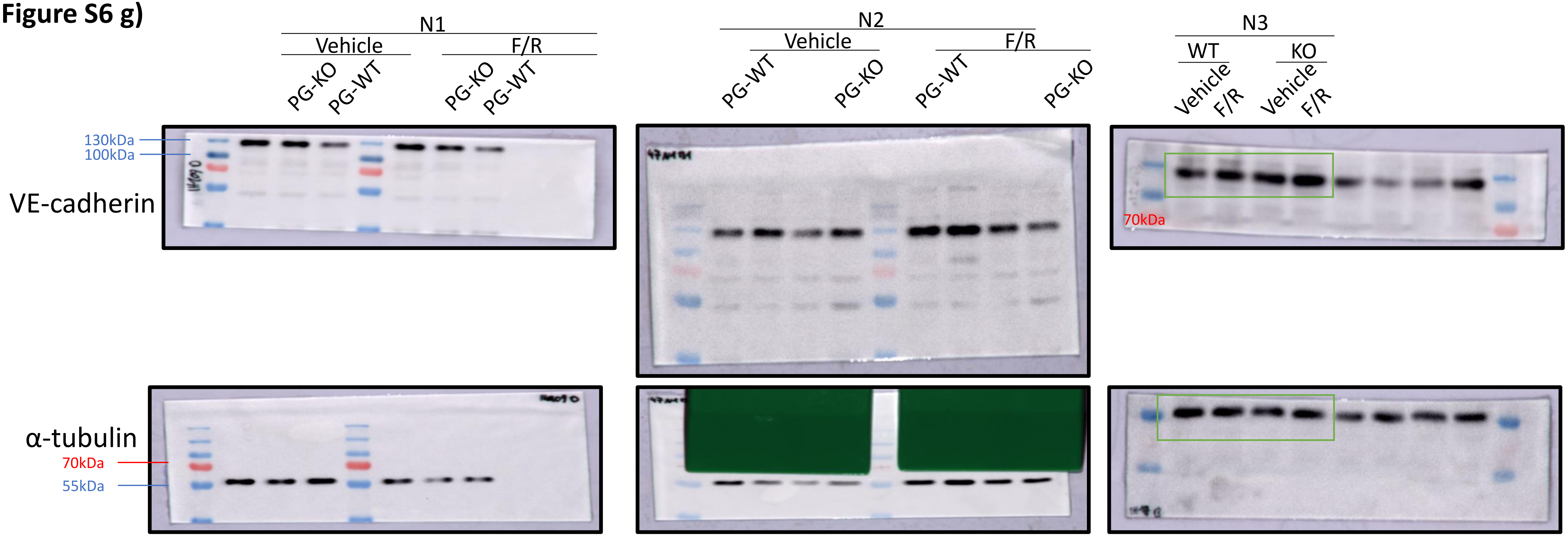
N1
Vehicle
F/R
PG-KO
PG-WT
PG-KO
PG-WT
N2
Vehicle
F/R
PG-WT
PG-KO
PG-WT
PG-KO
N3
WT
KO
Vehicle
F/R
Vehicle
F/R
130kDa
100kDa
VE-cadherin
70kDa
α-tubulin
70kDa
55kDa

Figure S6 h)

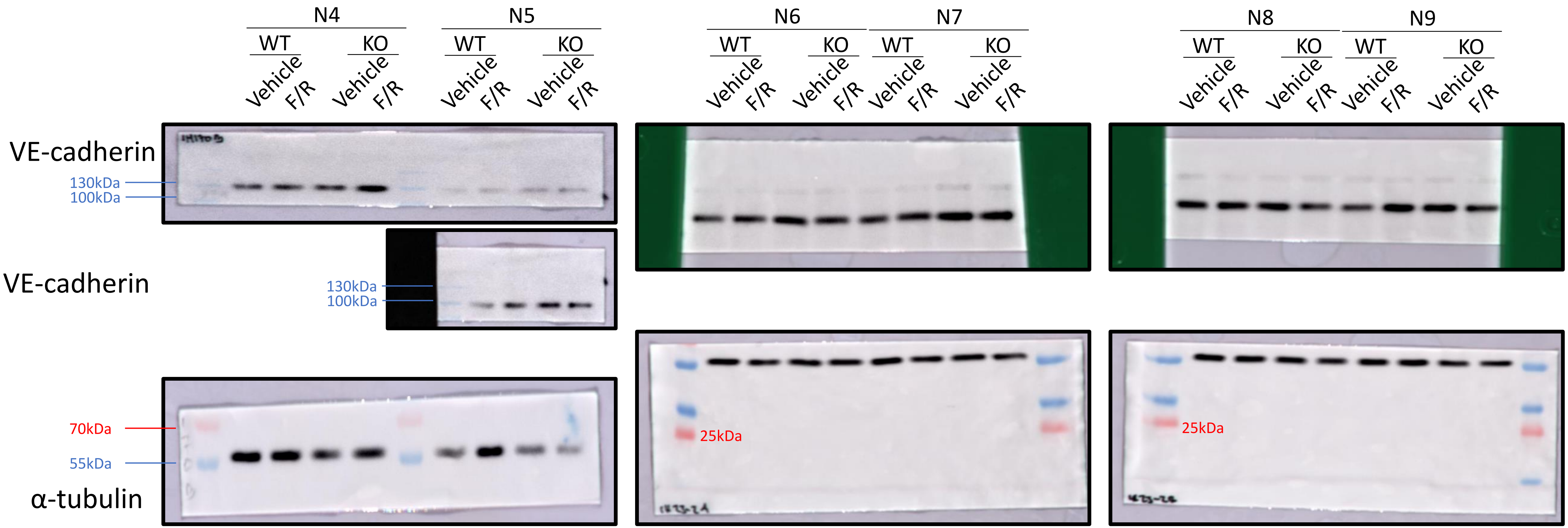
N4
N5
N6
N7
N8
N9
WT
KO
Vehicle
F/R
VE-cadherin
130kDa
100kDa
VE-cadherin
130kDa
100kDa
70kDa
55kDa
α-tubulin
25kDa
25kDa

Figure S6 i)

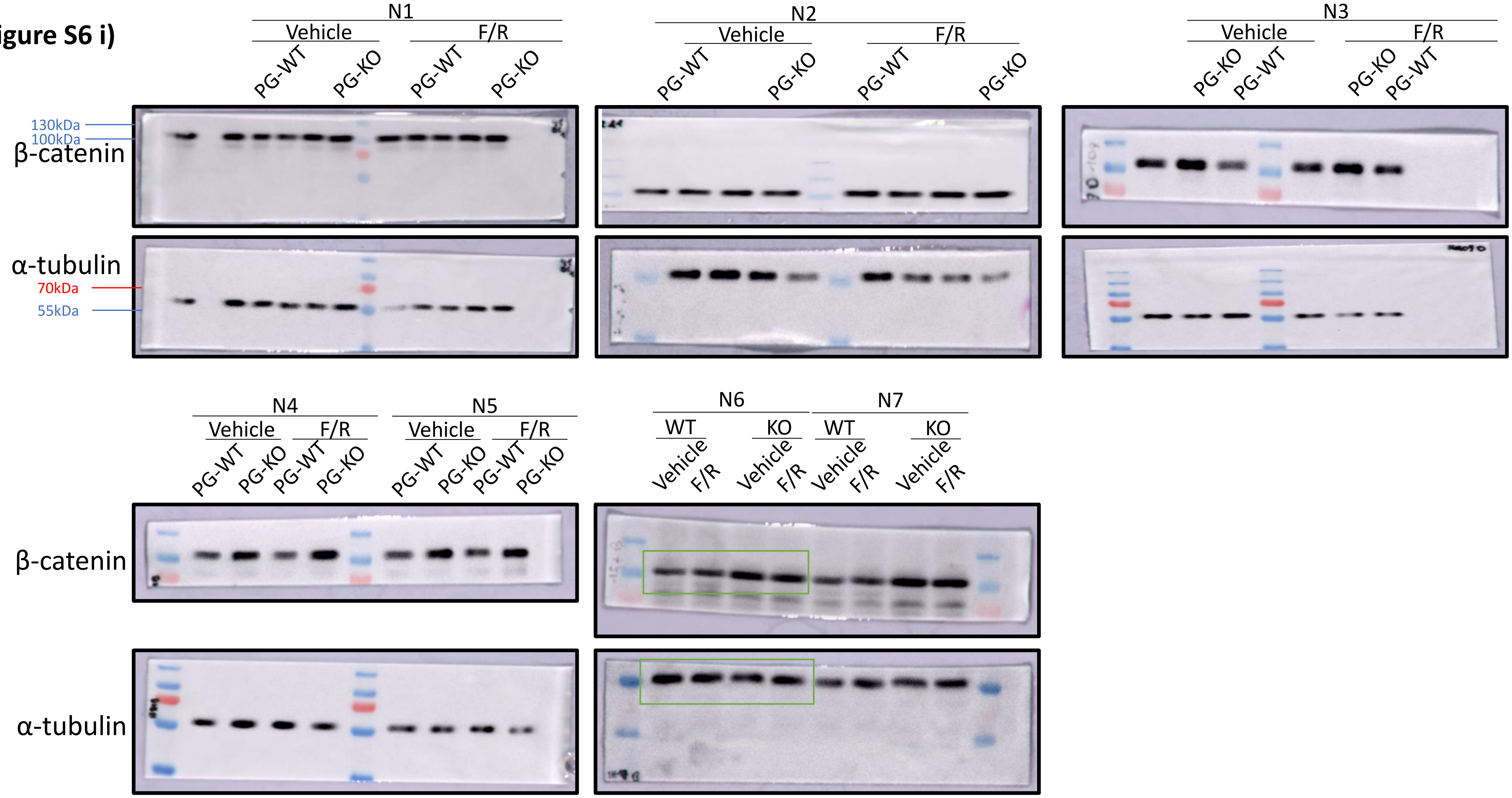
N1
Vehicle
F/R
PG-WT
PG-KO
PG-WT
PG-KO
N2
Vehicle
F/R
PG-WT
PG-KO
PG-WT
PG-KO
N3
Vehicle
F/R
PG-KO
PG-WT
PG-KO
PG-WT
130kDa
100kDa
β-catenin
α-tubulin
70kDa
55kDa
N4
Vehicle
F/R
PG-WT
PG-KO
PG-WT
PG-KO
N5
Vehicle
F/R
PG-WT
PG-KO
PG-WT
PG-KO
N6
WT
KO
Vehicle
F/R
Vehicle
F/R
N7
WT
KO
Vehicle
F/R
Vehicle
F/R
β-catenin
α-tubulin

Figure S6 j)

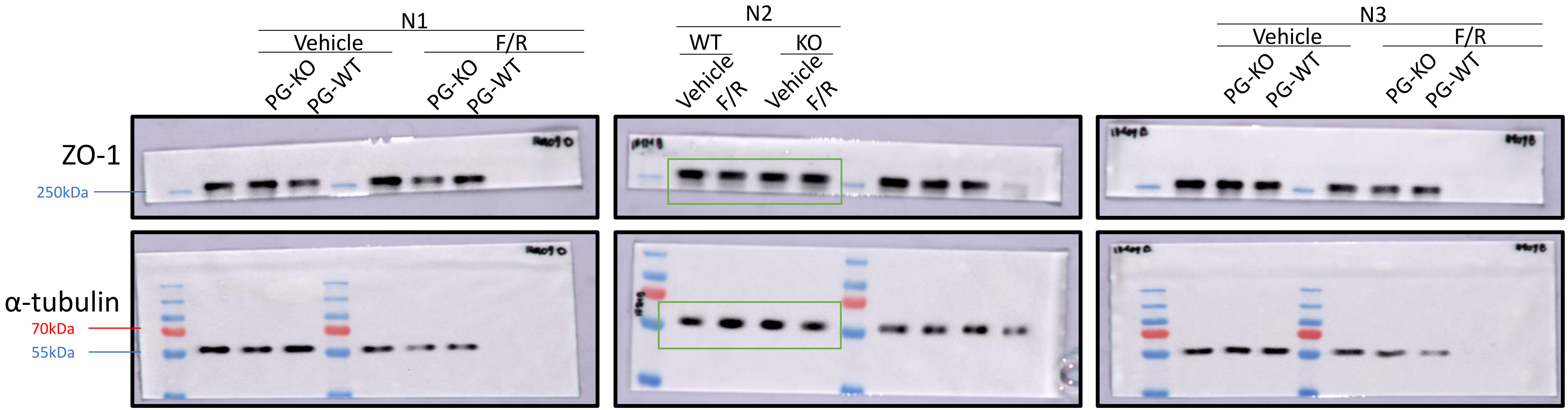
N1
Vehicle
F/R
PG-KO
PG-WT
PG-KO
PG-WT
N2
WT
KO
Vehicle
F/R
Vehicle
F/R
N3
Vehicle
F/R
PG-KO
PG-WT
PG-KO
PG-WT
ZO-1
250kDa
α-tubulin
70kDa
55kDa

Figure S6 k)

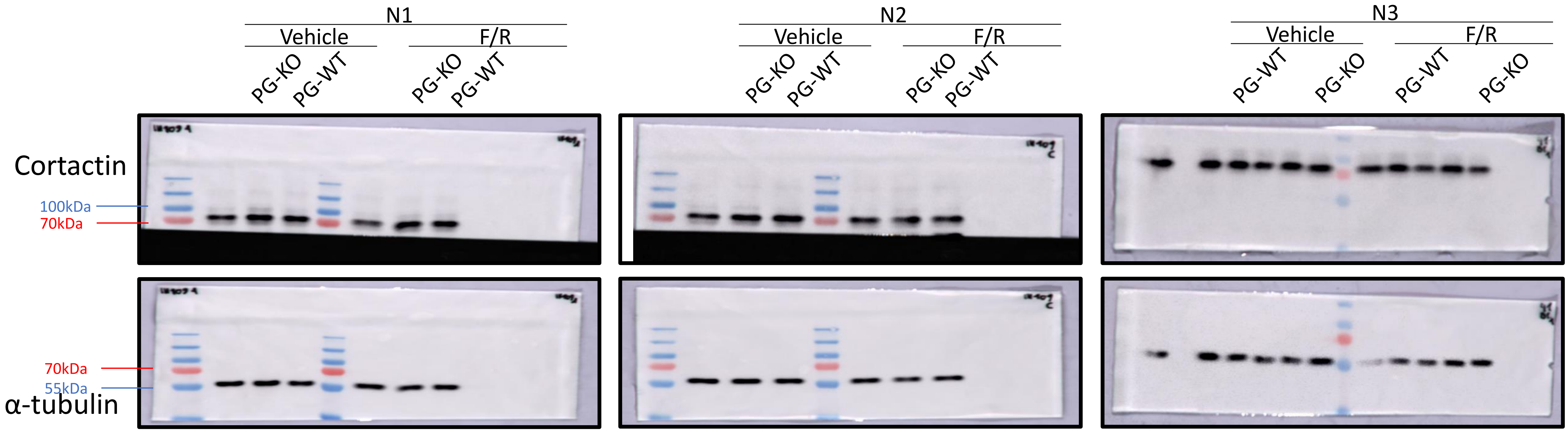

N1
Vehicle
F/R
PG-KO
PG-WT
PG-KO
PG-WT
N2
Vehicle
F/R
PG-KO
PG-WT
PG-KO
PG-WT
N3
Vehicle
F/R
PG-WT
PG-KO
PG-WT
PG-KO
Cortactin
100kDa
70kDa
70kDa
55kDa
α-tubulin

Figure S6 l)

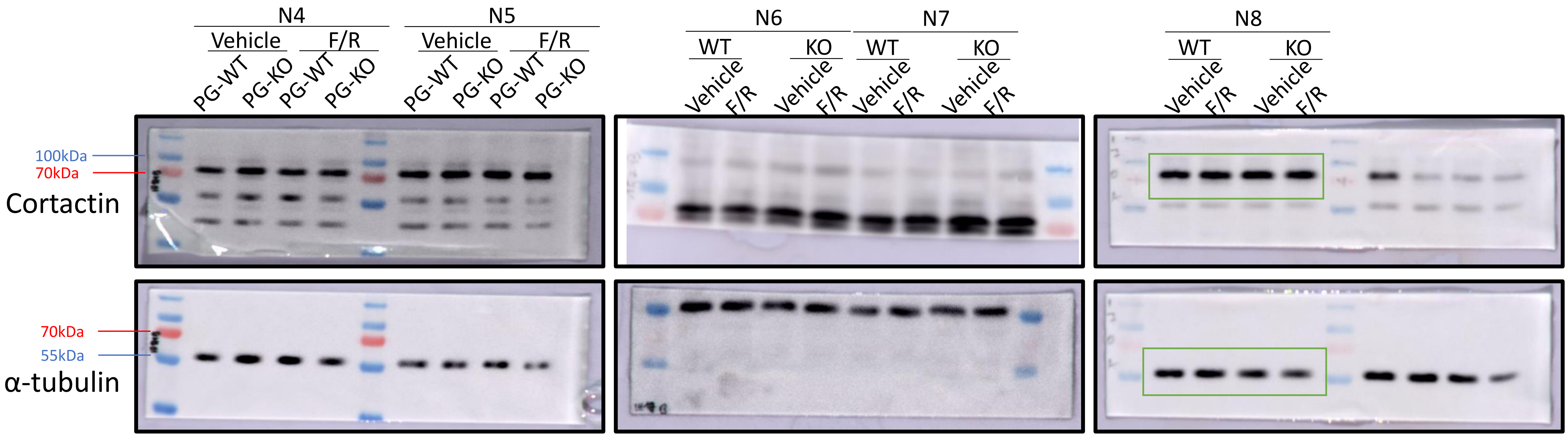

Figure S6, g-l) Original Western Blot gel images for vehicle or F/R treatment. To prevent over exposure, the black or green cover was used. The green square determines the selected blot used in the figure 6.

Figure S7 m)

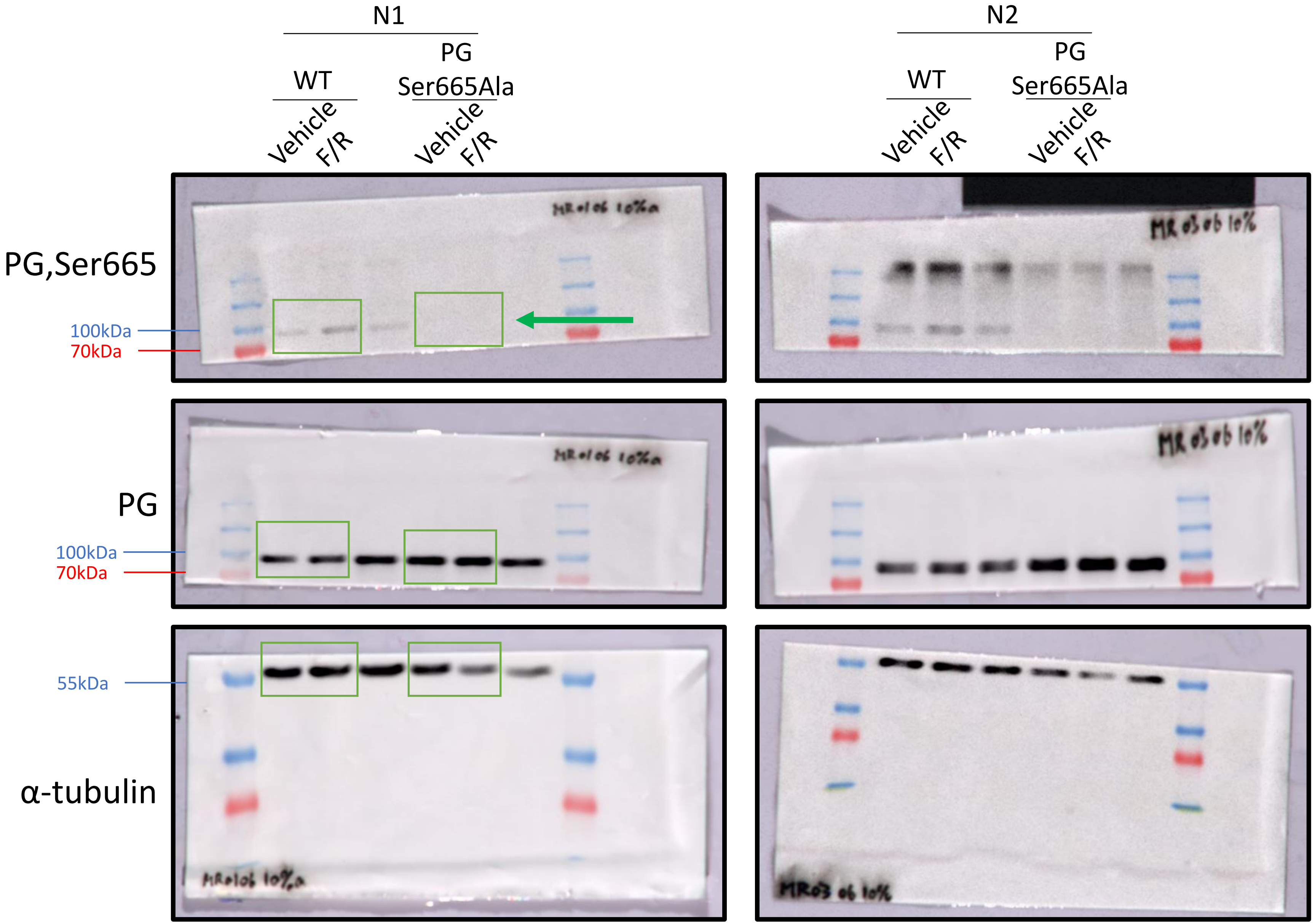

N1
WT
PG Ser665Ala
Vehicle
F/R
Vehicle
F/R
N2
WT
PG Ser665Ala
Vehicle
F/R
Vehicle
F/R
PG,Ser665
100kDa
70kDa
PG
100kDa
70kDa
55kDa
α-tubulin

Figure S7 n)

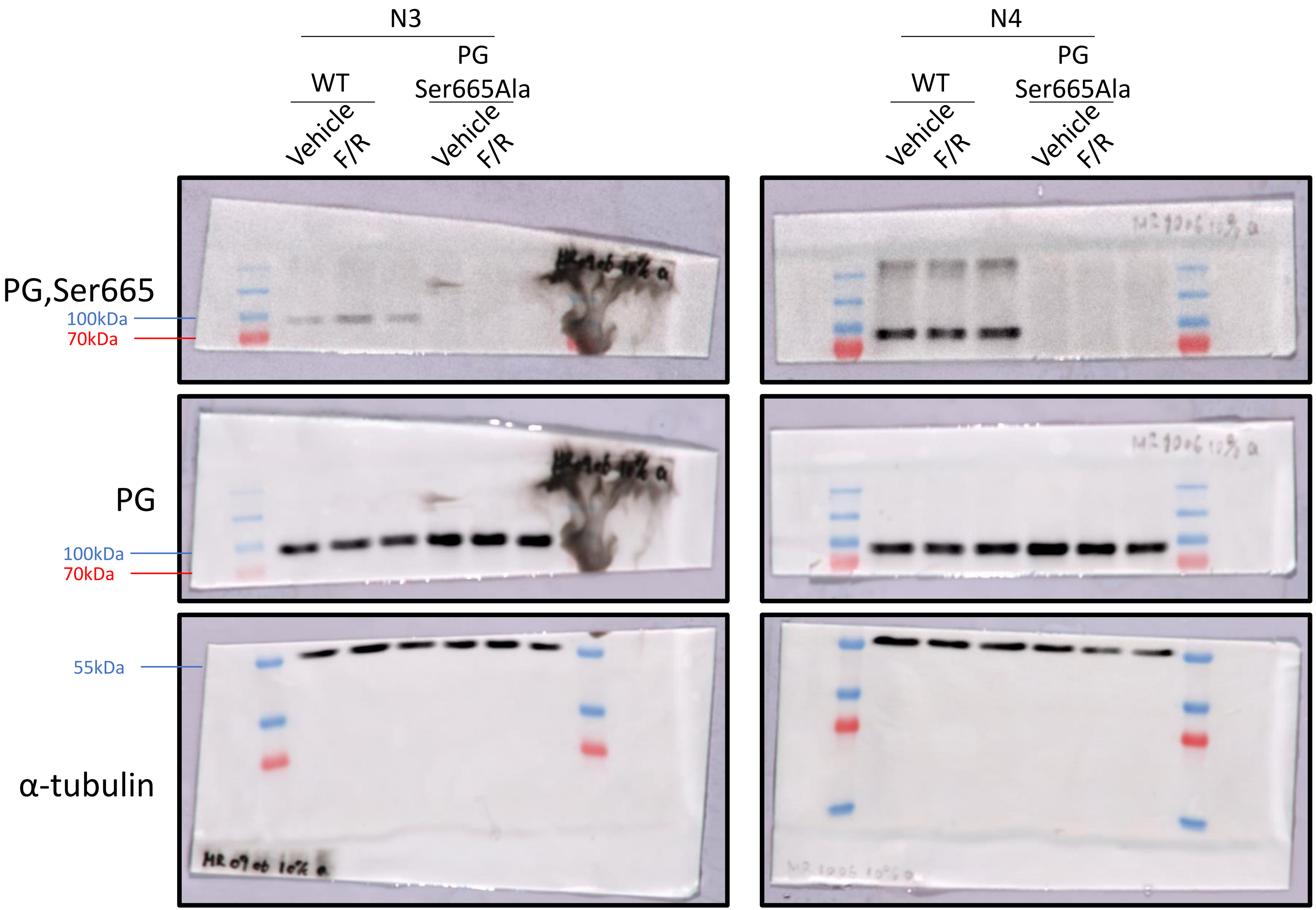

Figure S7, m-n) Original Western Blot gel images for vehicle or F/R treatment. To prevent over exposure, the black or green cover was used. The green square determines the selected blot used in the figure 7.

Original western blot gel images for VE-cadherin IPs

Figure S8 a)

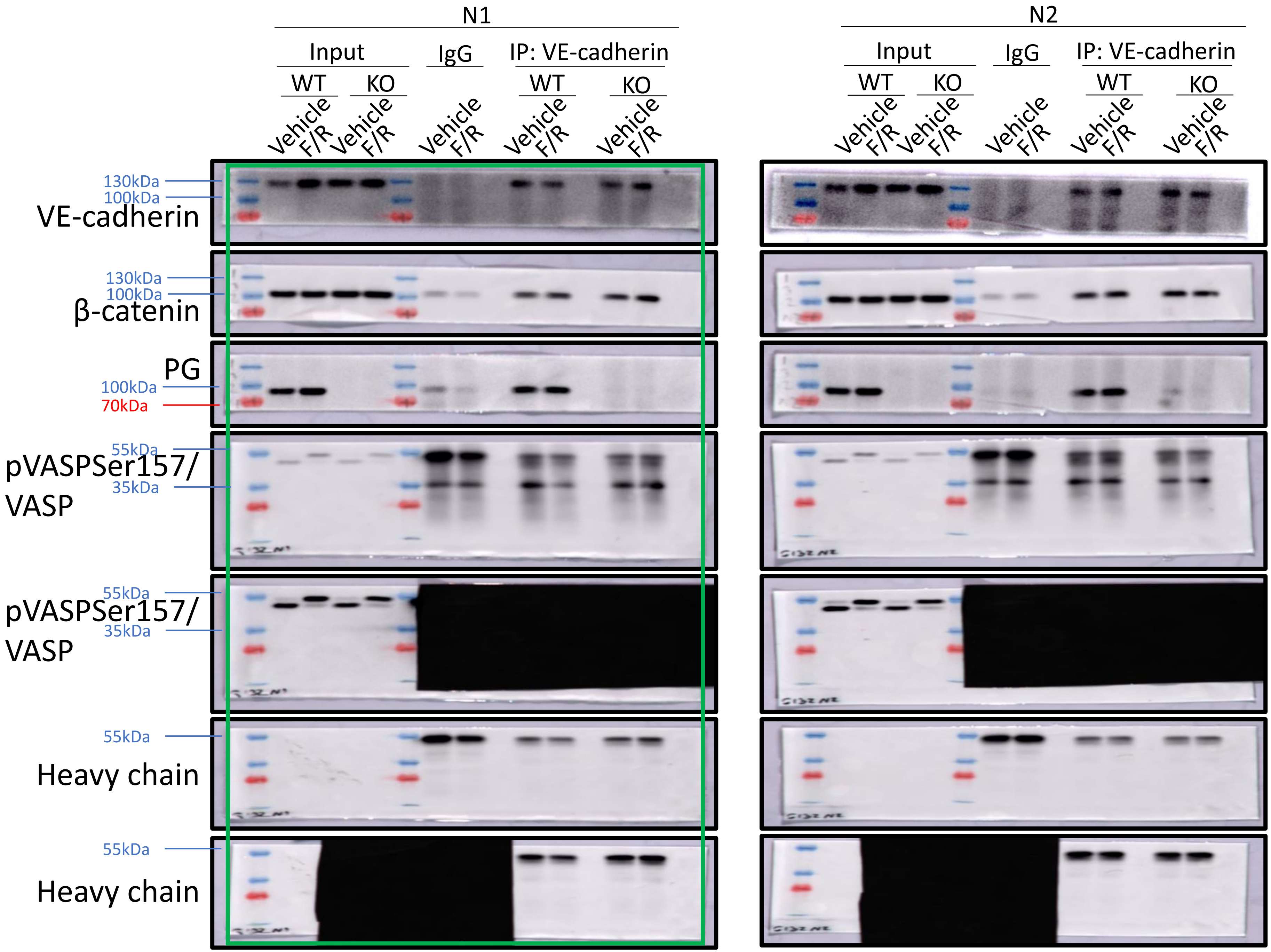
N1
N2
Input
IgG
IP: VE-cadherin
WT
KO
Vehicle
F/R
130kDa
100kDa
70kDa
55kDa
35kDa
VE-cadherin
β-catenin
PG
pVASPSer157/
VASP
Heavy chain

Figure S8 b)

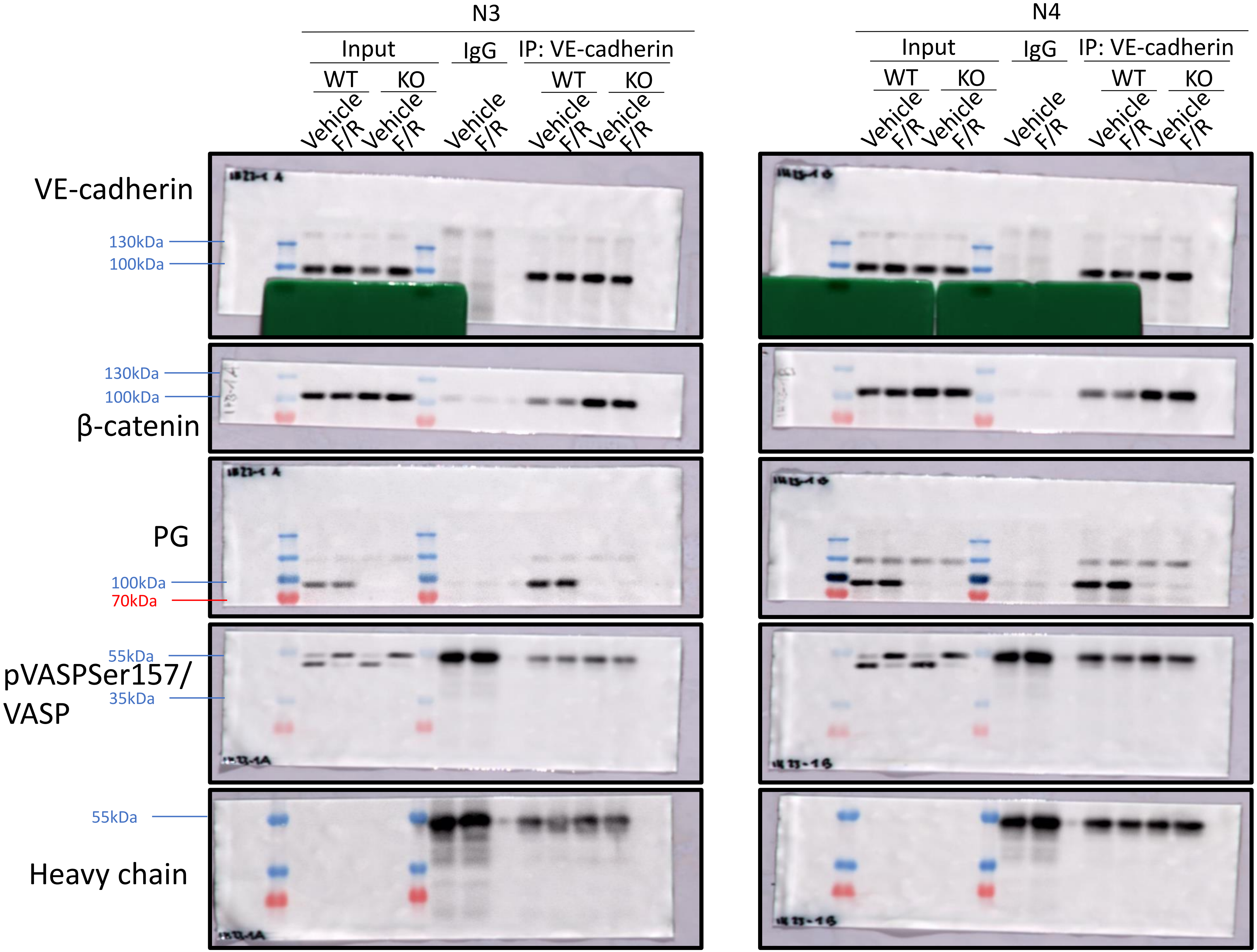

N3
Input
IgG
IP: VE-cadherin
WT
KO
WT
KO
Vehicle
F/R
N4
VE-cadherin
130kDa
100kDa
β-catenin
PG
70kDa
pVASPSer157/
VASP
55kDa
35kDa
Heavy chain

Figure S8 c)

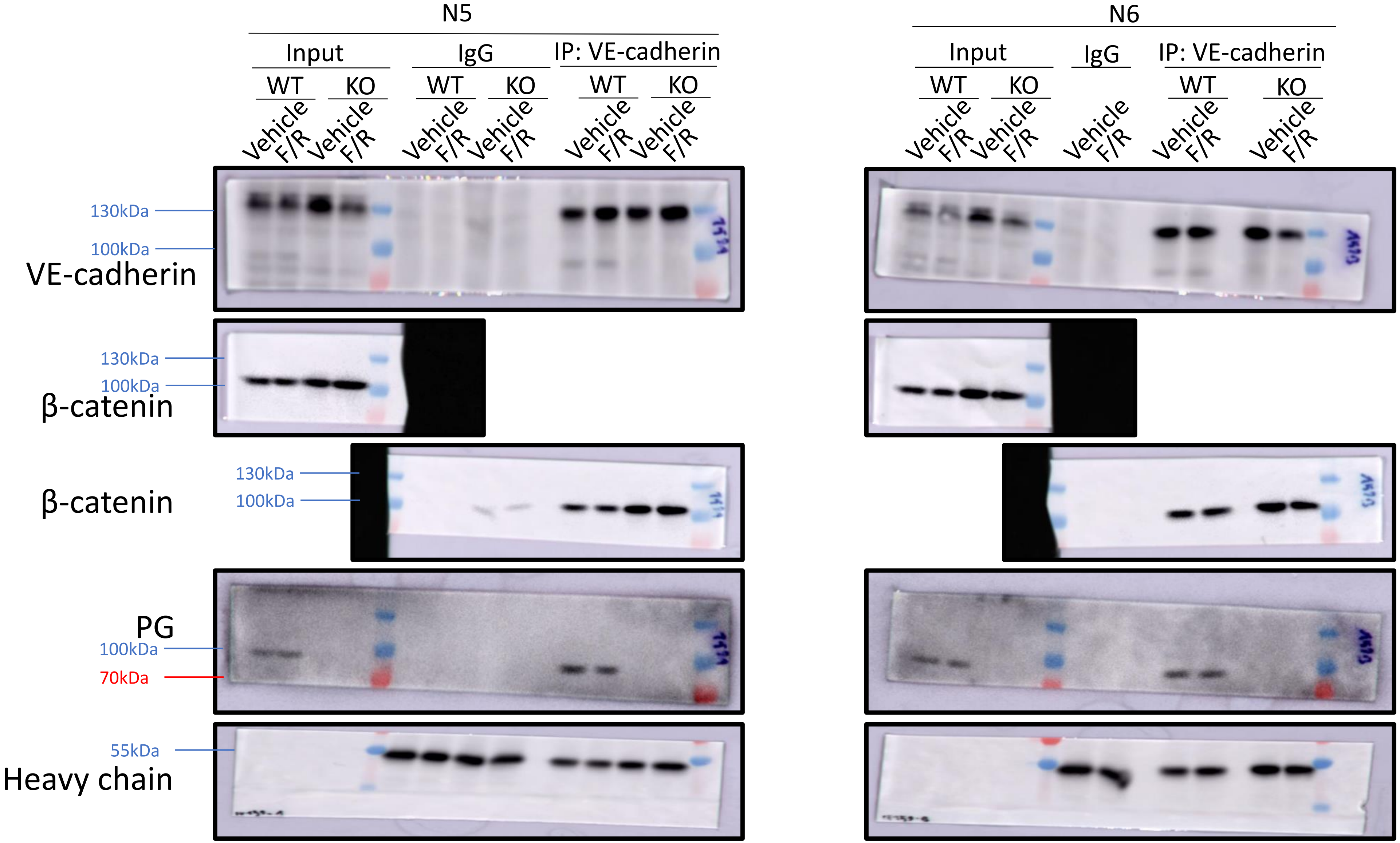

Figure S8, a-c) Original Western Blot gel images for VE-cadherin IPs. To prevent over exposure, the black or green cover was used. The green square determines the selected blot used in the figure 6.
